# Mammalian UPF3A and UPF3B can activate nonsense‐mediated mRNA decay independently of their exon junction complex binding

**DOI:** 10.15252/embj.2021109202

**Published:** 2022-04-22

**Authors:** Zhongxia Yi, René M Arvola, Sean Myers, Corinne N Dilsavor, Rabab Abu Alhasan, Bayley N Carter, Robert D Patton, Ralf Bundschuh, Guramrit Singh

**Affiliations:** ^1^ Center for RNA Biology The Ohio State University Columbus OH USA; ^2^ Department of Molecular Genetics The Ohio State University Columbus OH USA; ^3^ Department of Physics The Ohio State University Columbus OH USA; ^4^ Department of Chemistry and Biochemistry The Ohio State University Columbus OH USA; ^5^ Division of Hematology Department of Internal Medicine The Ohio State University Columbus OH USA

**Keywords:** exon junction complex, mRNA degradation, nonsense mutations, nonsense‐mediated mRNA decay, translation termination, Chromatin, Transcription & Genomics, RNA Biology

## Abstract

Nonsense‐mediated mRNA decay (NMD) is governed by the three conserved factors—UPF1, UPF2, and UPF3. While all three are required for NMD in yeast, UPF3B is dispensable for NMD in mammals, and its paralog UPF3A is suggested to only weakly activate or even repress NMD due to its weaker binding to the exon junction complex (EJC). Here, we characterize the UPF3A/B‐dependence of NMD in human cell lines deleted of one or both UPF3 paralogs. We show that in human colorectal cancer HCT116 cells, NMD can operate in a UPF3B‐dependent and ‐independent manner. While UPF3A is almost dispensable for NMD in wild‐type cells, it strongly activates NMD in cells lacking UPF3B. Notably, NMD remains partially active in cells lacking both UPF3 paralogs. Complementation studies in these cells show that EJC‐binding domain of UPF3 paralogs is dispensable for NMD. Instead, the conserved “mid” domain of UPF3 paralogs is consequential for their NMD activity. Altogether, our results demonstrate that the mammalian UPF3 proteins play a more active role in NMD than simply bridging the EJC and the UPF complex.

## Graphical abstract

Deletion of the NMD‐factor UPF3A and/or UPF3B in human cell lines reveals UPF3B‐dependent and ‐independent NMD.

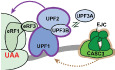

## Introduction

Nonsense mutations present a challenging obstacle for organisms as they result in premature termination of protein translation to produce truncated proteins that can be toxic for the cell. All eukaryotes deploy a conserved mechanism called nonsense‐mediated mRNA decay (NMD) to rapidly degrade mRNAs containing premature termination codons (PTCs) to limit the production of potentially toxic polypeptides. NMD has gained additional importance in more complex organisms as normal mutation‐free mRNAs take advantage of the NMD machinery to regulate their expression (reviewed in He & Jacobson, 2015; Karousis & Mühlemann, 2019; Kishor *et al*, 2019; Kurosaki *et al*, 2019). For example, in mammalian cells, ~10% of transcriptomes can be regulated by NMD (Mendell *et al*, 2004; Wittmann *et al*, 2006). The key task for the NMD machinery is to differentiate premature translation termination from normal translation termination on both nonsense mutation‐bearing and natural mRNAs that are degraded by this pathway. How the NMD machinery makes such a discrimination remains to be completely understood.

NMD depends on a set of core factors—UPF1, UPF2, and UPF3 that are conserved throughout eukaryotes. When translation terminates prematurely and much upstream of the normal 3′‐untranslated region (3′UTR) and poly(A)‐tail, UPF factors can recognize such termination events as premature via mechanisms that have been conceptualized into two possible (non‐mutually exclusive) models. One model suggests that termination in an altered 3′UTR context can compromise normal termination promoting interaction between release factors eRF3/eRF1 and the poly(A)‐tail binding protein (PABP) (Amrani *et al*, 2004; Behm‐Ansmant *et al*, 2007; Eberle *et al*, 2008; Ivanov *et al*, 2008; Singh *et al*, 2008; Peixeiro *et al*, 2012). Instead, UPF1 can engage with eRFs and initiate NMD (Kashima *et al*, 2006). According to the other model, longer 3′UTRs of NMD‐targeted mRNAs may serve as a distinction between normal and premature termination. By recruiting more UPF1, the central NMD activator that can non‐specifically bind RNA in a length‐dependent manner, longer 3′UTRs increase the likelihood of UPF1 engagement with terminating ribosome (Hogg & Goff, 2010). While the majority of available evidence points to a more direct role for UPF1 in engaging with release factors and the terminating ribosome (Kashima *et al*, 2006; Ivanov *et al*, 2008; Singh *et al*, 2008), a recent study shows that UPF3B, the dominant NMD factor among the two UPF3 paralogs, has a direct involvement in the termination reaction in human cell extracts (Neu‐Yilik *et al*, 2017). Nevertheless, the precise order of events and the mechanistic details of UPF functions at individual steps during premature termination remain poorly understood.

In mammalian cells, the NMD pathway has gained more complexity as it is tightly linked to pre‐mRNA splicing via the exon junction complex (EJC), which is of significant importance for NMD activation. The EJC is deposited on the mRNA exon‐exon junctions during splicing and is exported along with the mRNAs to the cytoplasm where it is stripped‐off from mRNAs by the first translating ribosomes (reviewed in Boehm & Gehring, 2016; Hir *et al*, 2016; Woodward *et al*, 2017). However, when PTCs lead to early translation termination, one or more EJCs that remain bound downstream of a terminated ribosome can stimulate NMD. As UPF3 paralogs directly interact with the EJC, the presence of EJC‐bound UPF3B in 3′UTRs is thought to promote UPF1 activation and premature termination via either of the two NMD models. Notably, in these models, UPF3B is mainly viewed as a bridge between the UPF and the EJC proteins (Chamieh *et al*, 2008; Melero *et al*, 2012). However, the functional relevance of such a bridging function, or if UPF3B‐EJC interaction serves another role, remains to be seen.

While all three UPF proteins are essential for NMD in yeast (He *et al*, 1997; Celik *et al*, 2017), UPF3 appears to have become less important for the overall operation of the NMD pathway in more complex organisms and some NMD can proceed even in its absence (reviewed in Yi *et al*, 2021). Unlike UPF1 or UPF2, a complete loss of UPF3 in *Drosophila* does not affect viability and has only a modest effect on NMD (Avery *et al*, 2011). In mammals, the available evidence suggest that UPF3B provides the main UPF3 activity due to its better EJC binding ability (Kunz *et al*, 2006). UPF3A, the other UPF3 paralog, can function as a weak NMD activator and can help to compensate for UPF3B function (Chan *et al*, 2009). A recent study has suggested that UPF3A may predominantly function as an NMD repressor, potentially by sequestering UPF2 away from NMD complexes via its strong UPF2 binding but weaker EJC binding (Shum *et al*, 2016). Surprisingly, despite being the dominant NMD activating UPF3 paralog with important biological roles, UPF3B knockout mice exhibit neurological abnormalities but no gross morphological defects (Huang *et al*, 2011, 2018). Similarly, UPF3B inactivating mutations in humans are non‐lethal but cause intellectual disability (Tarpey *et al*, 2007; Laumonnier *et al*, 2010) and are associated with neurodevelopmental disorders such as autism spectrum disorders and schizophrenia (Addington *et al*, 2011; Lynch *et al*, 2012; Xu *et al*, 2013). These observations suggest that while UPF3B is important for key biological processes, its contributions to the NMD pathway are likely to be more restricted as a total loss of NMD is lethal in vertebrates (Medghalchi *et al*, 2001; Weischenfeldt *et al*, 2008).

Consistent with an important but a more limited role for UPF3B in NMD, search for its mRNA targets in cell lines and primary cells from human patients and mouse models has uncovered transcripts that depend on UPF3B for their NMD (Chan *et al*, 2007; Huang *et al*, 2011, 2018; Nguyen *et al*, 2012; Karam *et al*, 2015; Domingo *et al*, 2020; Tan *et al*, 2020) and also those that do not require it for their efficient NMD (Chan *et al*, 2007; Huang *et al*, 2011). These observations have led to a proposal that the NMD pathway is bifurcated into a UPF3B‐dependent branch and a UPF3B‐independent branch (Chan *et al*, 2007; Huang *et al*, 2011; Yi *et al*, 2021). Several questions about such a branched nature of UPF3B function in NMD remain: Do these branches regulate unique or overlapping sets of mRNAs? How can NMD function in the absence of UPF3B? What is the contribution of UPF3A to such a dichotomy in UPF3 activity?

UPF3B function in NMD might be further affected by specific EJC compositions. Our previous work has demonstrated that EJC composition is heterogenous and, during different phases of the mRNA lifecycle, the EJC associates with a distinct set of peripheral factors (Mabin *et al*, 2018). The EJC co‐factor RNPS1 does not co‐exist in the same complex with another key EJC factor CASC3. Mass spectrometry of RNPS1 and CASC3‐containing EJCs showed that CASC3 but not RNPS1 preferentially associates with UPF3B (Mabin *et al*, 2018). Consistent with this observation, a recent report found a much‐reduced EJC‐UPF3B association in CASC3 knockout HEK293 cells (Gerbracht *et al*, 2020). Together, these observations suggest a link between EJC composition and UPF3B‐mediated NMD. The contribution of such a link to NMD and its underlying molecular basis remains to be fully understood.

Here, we created UPF3B knockout human cell lines with CRISPR‐Cas9 to study NMD in the presence and absence of UPF3B and to understand the relative flux through the UPF3B‐dependent and UPF3B‐independent branches of NMD. We find that NMD‐targeted transcripts including those with 3′UTR EJCs can undergo NMD in both UPF3B‐dependent and ‐independent manner. In the absence of UPF3B, and only under such conditions, UPF3A becomes responsible for a significant portion of UPF3B‐independent NMD. Surprisingly, our comparative analysis of UPF3A and UPF3B functions in NMD suggest that both UPF3 paralogs remain potent NMD activators even without their ability to bind EJC, hinting that another function may form the primary basis of their NMD activation. We also find that EJC binding of the UPF3 paralogs is assisted by CASC3 and may play a more secondary role in NMD by recruiting UPF3 proteins to mRNAs.

## Results

### UPF3B loss of function only partially inhibits the NMD pathway

**Figure 1:**
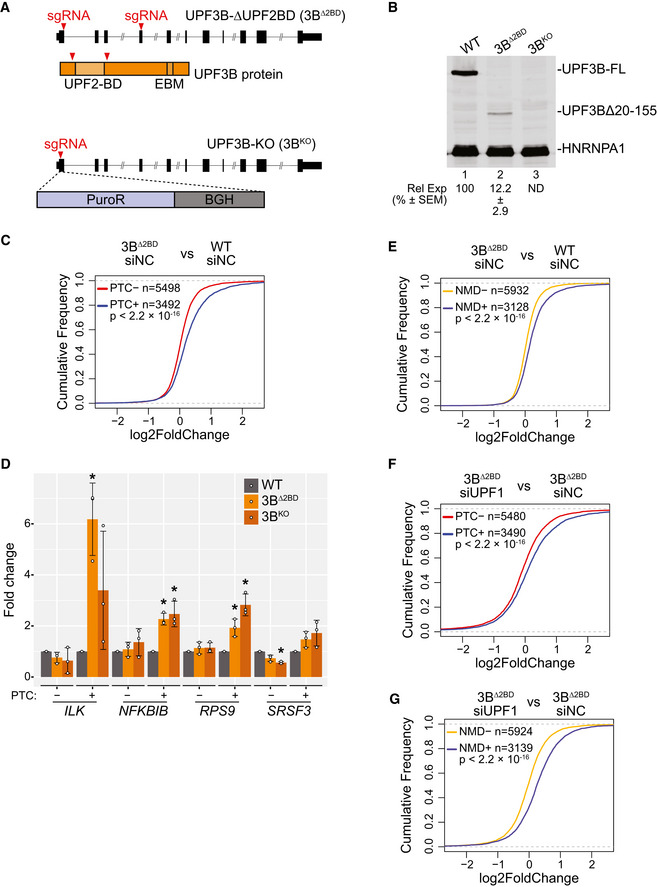
Loss of *UPF3B* in human cells affects EJC‐dependent NMD Schematic of *UPF3B* mutagenesis strategies using CRISPR‐Cas9. *UPF3B* locus is in black where rectangles represent exons and horizontal line denotes introns; coding region is shown as wider rectangles. Red arrowheads represent guide RNA targeting sites. Top: two guide RNAs delete the UPF2 binding domain (2BD) of UPF3B protein coding region as shown to create 3B^Δ2BD^ cells. Bottom: a donor template is used to insert puromycin resistant gene (PuroR) and bovine growth hormone (BGH) polyadenylation signal at the cut site to generate UPF3B knockout (3B^KO^) cells.Immunoblot of parental wild‐type (WT) and UPF3B mutant cell lines showing levels of proteins on the right. In 3B^Δ2BD^, a smaller UPF3B protein with deletion of amino acids 20‐155 (UPF3BΔ20‐155) is expressed. Relative Expression (Rel Exp) of this deletion protein as compared to the full‐length UPF3B along with standard error of mean (SEM) are indicated below lane 2. UPF3B antibody recognizes antigen outside the deleted region in 3B^Δ2BD^. HNRNPA1 is used as a loading control. ND = UPF3B levels not determined.Cumulative Distribution Function (CDF) plots of PTC^+^ isoforms and PTC^−^ isoforms from the same set of genes. X‐axis represents fold change in 3B^Δ2BD^ versus WT cells each with control knockdown (siNC). Number of transcripts in each set (*n*) and *P*‐value from Kolmogorov–Smirnov (KS) test comparing the two distributions are shown.Bar plots from isoform specific RT‐qPCR analysis showing average fold change (*y*‐axis) of PTC^+^ and PTC^−^ isoforms from genes indicated on the bottom in WT and the two UPF3B mutant cells identified in the legend on the top right. For each isoform, levels in mutant cells are compared to the levels in WT cells (set to 1). Relative levels from each replicate are shown by white circles. Error bars indicate standard errors of means. The asterisk (*) represents *P* < 0.05 in *t*‐test with null hypothesis of true mean being 1 (*n* = 3 biological replicates).Cumulative Distribution Function (CDF) plots of NMD^+^ isoforms and NMD^−^ isoforms. X‐axis represents fold change in 3B^Δ2BD^ versus WT cells each with control knockdown (siNC). Number of transcripts in each set (*n*) and *P*‐value from Kolmogorov–Smirnov (KS) test comparing the two distributions are shown.Cumulative Distribution Function (CDF) plots of PTC^+^ isoforms and PTC^−^ isoforms from same set of genes. X‐axis represents fold change in UPF1 knockdown (siUPF1) versus control knockdown (siNC) in 3B^Δ2BD^ cells. Number of transcripts in each set (*n*) and *P*‐value from Kolmogorov–Smirnov (KS) test comparing the two distributions are shown.Cumulative Distribution Function (CDF) plots of NMD^+^ isoforms and NMD^−^ isoforms. X‐axis represents fold‐change in UPF1 knockdown (siUPF1) versus control knockdown (siNC) in 3B^Δ2BD^ cells. Number of transcripts in each set (*n*) and *P*‐value from Kolmogorov–Smirnov (KS) test comparing the two distributions are shown. Source data are available online for this figure.

**Figure EV1:**
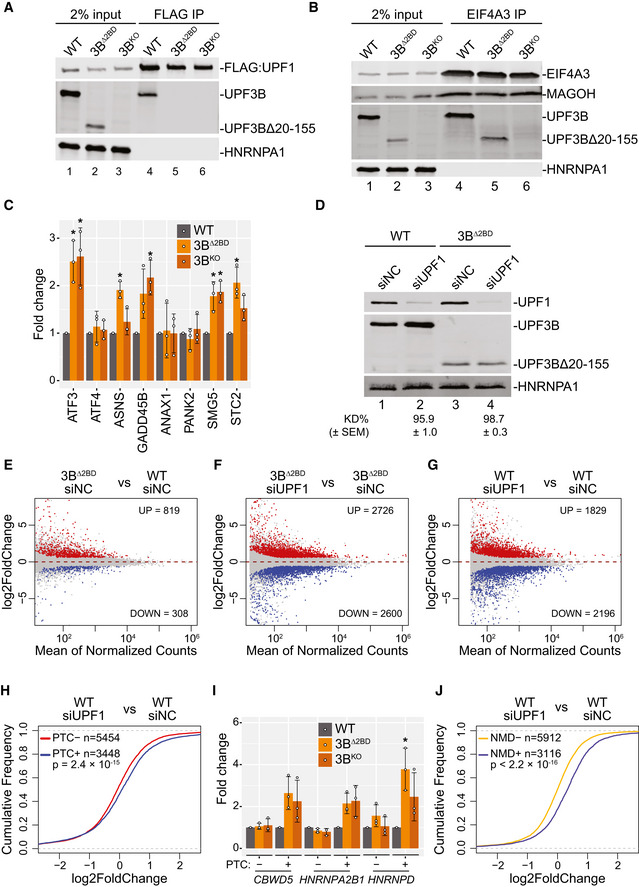
Changes in gene expression and NMD upon *UPF3B* loss in HCT116 cells AImmunoblots showing levels of proteins on the right in input or FLAG immunoprecipitates (IP) from WT and UPF3B mutant cells (indicated above each lane) expressing FLAG‐tagged UPF1 protein.BImmunoblots showing levels of proteins on the right in input or EIF4A3 IP from WT and UPF3B mutant cells as indicated above each lane.CAlteration in expression levels of known NMD‐regulated genes in the two UPF3B mutant cell lines. RT‐qPCR‐based quantification of expression levels of previously characterized NMD‐sensitive genes (x‐axis) in the two UPF3B mutant HCT116 cell lines as compared to their levels in WT cells (set to 1). Relative levels from each replicate are shown by white circles. Error bars indicate standard error of means. The asterisk (*) represents *P* < 0.05 in *t*‐test with null hypothesis of true mean being 1 (*n* = 3 biological replicates).DImmunoblot showing levels of UPF1 and UPF3B proteins in WT and 3B^Δ2BD^ cells (indicated on top) that were transfected with negative control (siNC) or UPF1‐targeting (siUPF1) siRNAs. HNRNPA1 is a loading control. Knockdown percentage (KD%) of UPF1 in siUPF1‐transfected cells as compared to siNC‐transfected cells is shown with standard error of means (SEM).E–GMA plots showing differential transcript expression in RNA‐Seq samples from (E) 3B^Δ2BD^ cells (siNC) versus WT cells (siNC), (F) UPF1‐KD (siUPF1) versus control knockdown (siNC) in 3B^Δ2BD^ cells, and (G) UPF1‐KD (siUPF1) versus control knockdown (siNC) in WT cells. Each dot represents one transcript isoform with average read counts on the x axis and log_2_ fold change on the y axis. Transcripts that are significantly (adjusted *P*‐value < 0.05) up (red)‐ or down (blue)‐ regulated > 1.5‐fold and their counts are indicated.HCumulative Distribution Function (CDF) plots of PTC^+^ isoforms and PTC^−^ isoforms from same set of genes. X‐axis represents fold change in UPF1‐KD (siUPF1) versus control knockdown (siNC) in WT cells. Number of transcripts in each set (*n*) and *P*‐value from Kolmogorov‐Smirnov (KS) test comparing the two distributions are shown.IIsoform specific RT‐qPCR measuring changes in levels of PTC^+^ and PTC^−^ isoforms expressed from the indicated genes in WT and UPF3B mutant cells. Fold changes are with respect to the levels of PTC^−^ isoforms in WT cells. Relative levels from each replicate are shown by white circles. Error bars indicate standard errors of means. The asterisk (*) represents *P* < 0.05 in *t*‐test with null hypothesis of true mean being 1 (*n* = 3 biological replicates).JCumulative Distribution Function (CDF) plots of NMD^+^ isoforms and NMD^−^ isoforms. X‐axis represents fold change in UPF1‐KD (siUPF1) versus control knockdown (siNC) in WT cells. Number of transcripts in each set (*n*) and *P*‐value from Kolmogorov‐Smirnov (KS) test comparing the two distributions are shown. Source data are available online for this figure.

To study UPF3B‐independent NMD, we used CRISPR‐Cas9‐based gene editing to generate two independent *UPF3B* loss of function alleles in human colorectal carcinoma HCT116 cells, a near diploid cell line with only one copy of *UPF3B*. In the first approach, we deleted ~8 kilobase genomic region of the *UPF3B* locus that spans exons 1–4 and encodes the UPF2‐binding RNA recognition motif (RRM)‐like domain of UPF3B (3B^Δ2BD^; Fig 1A). We reason that the loss of this key functional domain will lead to a complete loss of UPF3B function during NMD. As expected, in 3B^Δ2BD^ cell line, a smaller protein lacking amino acids 20–155 is expressed at ~12% levels of the full‐length protein observed in the “parental wild‐type” (WT) HCT116 cells (Fig 1B). This truncated UPF3B lacks interaction with UPF1 (Fig EV1A) but can still interact with the EJC (Fig EV1B). For the second allele, we used homology‐directed repair to insert immediately downstream of the *UPF3B* start codon a puromycin resistance marker followed by a polyadenylation signal that is expected to generate a truncated transcript lacking most *UPF3B* exons (Fig 1A). The resulting puromycin resistant cells completely lack UPF3B (3B^KO^) (Fig 1B). A qPCR survey in the two mutant cell lines revealed that mRNA levels of several previously characterized NMD‐regulated genes are similarly upregulated, whereas a subset of these genes remain largely unchanged in the two cell lines (Fig EV1C). Thus, the HCT116 UPF3B mutant cells represent an appropriate model to study UPF3B‐dependent and ‐independent NMD.

To identify the transcriptome‐wide effect of UPF3B loss on NMD, we performed RNA‐Seq from WT and 3B^Δ2BD^ HCT116 cells transfected with either negative control siRNA (siNC) or UPF1 targeting siRNA (siUPF1) (Fig EV1D). The mRNAs upregulated in UPF3B mutant cells as compared with WT cells can be considered UPF3B‐dependent NMD targets. As UPF1 is required for all NMD, mRNAs upregulated only after UPF1 knockdown (KD) in 3B^Δ2BD^ cell line can be considered as UPF3B‐independent NMD targets. Gene expression quantification at mRNA isoform level followed by differential expression analyses showed that, as expected upon disruption of an mRNA repressive factor such as UPF3B, the number of upregulated transcripts in 3B^Δ2BD^ cells as compared with WT cells (819 transcripts) is much higher as compared with those downregulated (308 transcripts) (Fig EV1E). However, the effects on the transcriptome after UPF3B loss of function are smaller as compared with the UPF1 knockdown in either 3B^Δ2BD^ cells (2,726 upregulated and 2,600 downregulated transcripts; Fig EV1F) or WT cells (1,829 upregulated and 2,196 downregulated transcripts; Fig EV1G). These observations support a more restricted role of UPF3B than UPF1 in gene regulation.

We next examined the effect of UPF3B loss of function on NMD using two approaches. First, we focused on a specific class of genes that produce two types of transcript isoforms, one with an exon‐exon junction ≥50 nucleotides downstream of a stop codon (PTC^+^ transcripts) and one that lacks this well‐known NMD‐inducing feature (PTC^−^ transcripts). Any change in NMD upon UPF3B loss of function is expected to alter only the PTC^+^ isoforms whereas any indirect effects of such a manipulation on gene expression (Tani *et al*, 2012) are expected to similarly impact both the PTC^+^ and the PTC^−^ isoforms. A comparison of transcript isoform levels in 3B^Δ2BD^ versus WT cells shows a significant upregulation of PTC^+^ isoforms over PTC^−^ isoforms (Fig 1C; a rightward shift of the PTC^+^ distribution as compared with the PTC^−^ distribution signifies the upregulation of PTC^+^ group of transcripts), similar to the trend observed in UPF1‐KD versus control cells (Fig EV1H). We confirmed the specific and significant (in most cases) upregulation of PTC^+^ isoforms predicted (but not tested) to undergo EJC‐dependent NMD as compared with the PTC^−^ isoforms of several genes in UPF3B^Δ2BD^ and 3B^KO^ cell lines via a qPCR assay (Figs 1D and EV1I). These results suggest that UPF3B is required for the efficient downregulation of EJC‐dependent NMD targets in HCT116 cells. Next, to test for the UPF3B requirement beyond the EJC‐dependent NMD branch, we focused on transcripts that have been experimentally determined as NMD targets in human cells. Using RNA‐Seq datasets from UPF1, SMG6, and SMG7 knockdown HeLa cells (Colombo *et al*, 2017), we defined significantly upregulated (> 1.2‐fold) transcripts upon depletion of at least two of the three proteins as NMD targets (NMD^+^). As control, transcripts that are minimally affected (less than 1.2‐fold change in either direction) in two of the three conditions were considered as NMD insensitive (NMD‐) group. Expectedly, the NMD^+^ group of transcripts show a robust upregulation as compared with the NMD^−^ group upon UPF1 knockdown in HCT116 cells (Fig EV1J). A comparison of transcript fold changes in 3B^Δ2BD^ versus WT cells shows that the NMD^+^ group of transcripts are significantly more upregulated than the NMD^−^ transcripts (Fig 1E) suggesting that UPF3B loss of function significantly impacts the overall NMD pathway in HCT116 cells.

We next examined the extent to which the NMD pathway remains active in cells lacking functional UPF3B. If NMD can still occur under such conditions, we expect additional upregulation of NMD‐sensitive (PTC^+^ and NMD^+^) transcripts as compared with NMD insensitive (PTC^−^ and NMD^−^) transcripts when the NMD pathway is further compromised by UPF1 depletion in UPF3B mutant cells. In line with this prediction, after UPF1 knockdown in 3B^Δ2BD^ cells, we observe a significant global upregulation of both PTC^+^ and NMD^+^ transcripts as compared with their respective control groups (Fig 1F and G). Thus, EJC‐dependent NMD and even the general NMD pathway can continue to operate partially even in the absence of functional UPF3B.

### UPF3A replaces UPF3B in EJC‐UPF complexes in UPF3B‐deficient cells

**Figure EV2:**
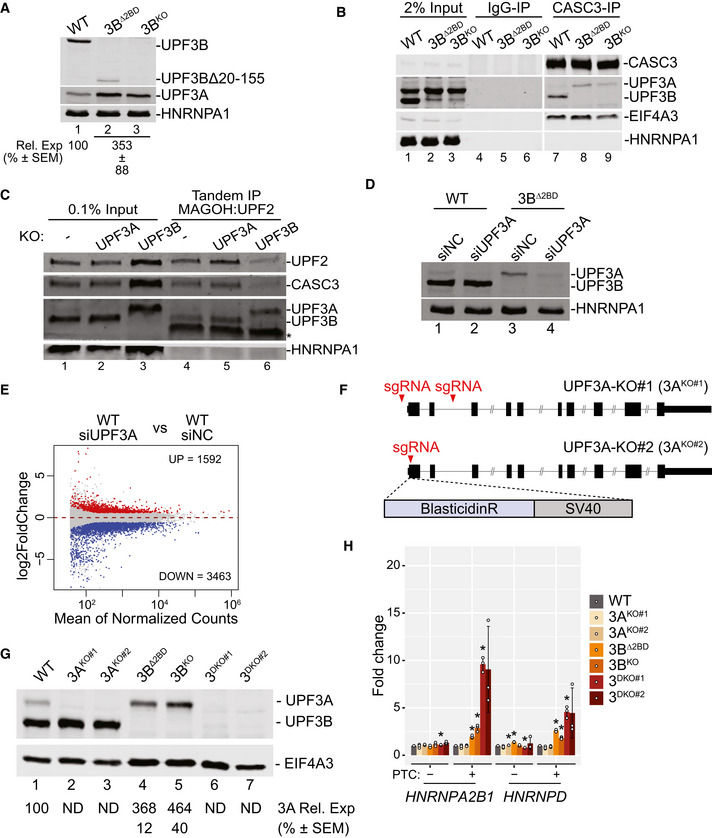
UPF3A activates NMD in the absence of UPF3B Immunoblots showing levels of proteins on the right in cells indicated above each lane. At the bottom are relative UPF3A levels after normalization to HNRNPA1 levels.Western blots showing levels of EJC/UPF proteins or HNRNPA1 in input, normal rabbit IgG‐IP or CASC3‐IP fractions from WT and UPF3B mutant cells indicated above each lane.Western blots showing levels of EJC/UPF proteins or HNRNPA1 in input or FLAG‐MAGOH followed by MYC‐UPF2 tandem‐IP fractions from WT, 3A^KO^, and 3B^KO^ cells. Samples were RNase A treated during the FLAG IP. The asterisk (*) represents the mouse heavy chain of the MYC‐tag antibody.Immunoblot showing levels of UPF3A and UPF3B proteins in WT and 3B^Δ2BD^ cells (indicated on top) that were transfected with negative control (siNC) or UPF3A‐targeting (siUPF3A) siRNAs. HNRNPA1 is a loading control.MA plot showing differential transcript expression in RNA‐Seq samples from UPF3A‐KD (siUPF3A) versus control knockdown (siNC) in WT HCT116 cells. Each dot represents one transcript isoform with average read counts on the x‐axis and log_2_ fold change on the y‐axis. Red and blue dots represent > 1.5‐fold up‐ or down‐ regulated transcripts, respectively, that are significantly changed (adjusted *P*‐value < 0.05).Schematic of *UPF3A* knockout (UPF3A‐KO) strategies using CRISPR‐Cas9. *UPF3A* locus is in black where rectangles represent exons and horizontal line denotes introns; coding region is shown as wider rectangles. Red arrowheads represent guide RNA targeting sites. In 3A^KO#1^ (top), two guide RNAs delete first and the second exons of UPF3A protein coding region. In 3A^KO#2^ (bottom), a donor template is used to insert blasticidin resistant gene (BlasticidinR) and Simian Virus 40 (SV40) polyadenylation signal at the cut site.Immunoblot of UPF3A and UPF3B proteins in WT, 3A^KO^, 3B^KO^, and 3^DKO^ cells. EIF4A3 is used as a loading control. Relative expression of UPF3A (3A Rel. Exp) in 3B^Δ2BD^ and 3B^KO^ as compared to WT cells is shown along with standard error of means (SEM) below lanes (ND = not determined).Isoform specific RT‐qPCR of PTC^+^ and PTC^−^ isoforms from the indicated genes in WT, 3A^KO^, 3B^Δ2BD^, 3B^KO^, and 3^DKO^ cells. Relative levels from each replicate are shown by white circles. Error bars indicate standard errors of means. The asterisk (*) represents *P* < 0.05 in *t*‐test with null hypothesis of true mean being 1 (*n* = 3 biological replicates).

**Figure 2:**
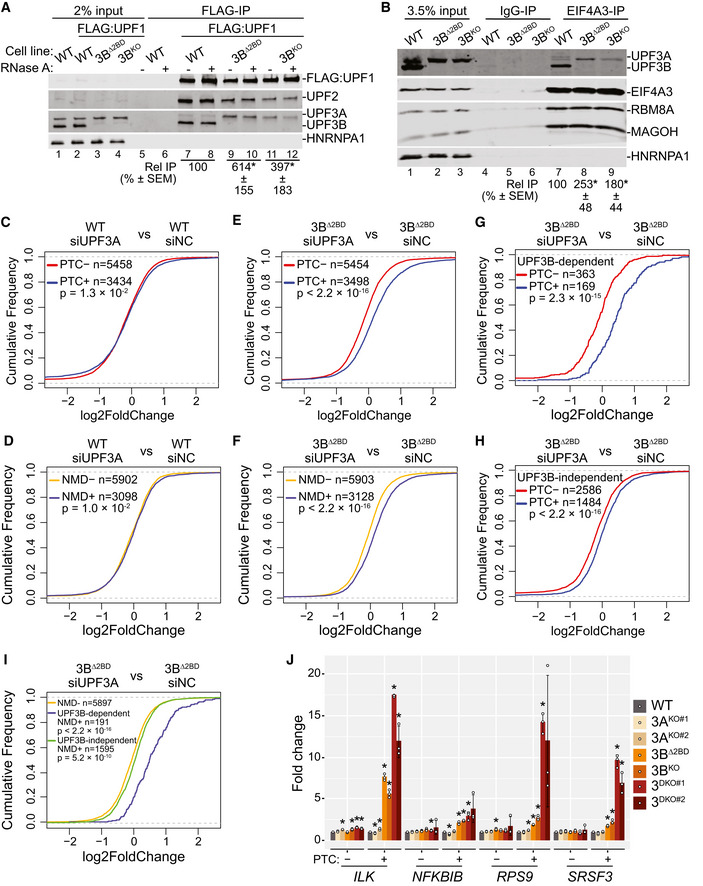
UPF3A activates NMD in the absence of UPF3B AImmunoblots showing levels of proteins on the right in input or FLAG immunoprecipitates (IP) from WT and UPF3B mutant cells expressing endogenously FLAG‐tagged UPF1 protein as indicated above each lane. The presence of RNase A during FLAG‐IP is indicated above each lane. The asterisk (*) represents *P* < 0.05 in *t*‐test with null hypothesis of true mean being 1.BImmunoblots showing levels of proteins (right) in input and IP with normal rabbit IgG (IgG‐IP) or antibody targeting EIF4A3 (EIF4A3‐IP) from WT and UPF3B mutant cells. The asterisk (*) represents *P* < 0.05 in *t*‐test with null hypothesis of true mean being 1.C, DCDF plots of (C) PTC^+^ and PTC‐isoforms, (D) NMD^+^ and NMD^−^ isoforms. X‐axis represents fold‐change upon UPF3A knockdown (siUPF3A) versus negative control knockdown (siNC) in WT cells. Number of transcripts in each set (*n*) and *P*‐value from KS test comparing the two distributions are shown on each plot.E, FCDF plots of (E) PTC^+^ and PTC‐isoforms, (F) NMD^+^ and NMD^−^ isoforms. X‐axis represents fold change upon UPF3A knockdown (siUPF3A) versus negative control knockdown (siNC) in 3B^Δ2BD^ cells. Number of transcripts in each set (*n*) and *P*‐value from KS test comparing the two distributions are shown on each plot.G, HCDF plots of UPF3B‐dependent (G) and ‐independent (H) PTC^+^ isoforms and their respective PTC^−^ isoforms. X‐axis represents fold‐change upon UPF3A knockdown (siUPF3A) versus negative control knockdown (siNC) in 3B^Δ2BD^ cells. Number of transcripts in each set (*n*) and *P*‐value from KS test comparing the two distributions are shown on each plot.ICDF plots of UPF3B‐dependent and UPF3B‐independent NMD^+^ isoforms as compared with NMD^−^ isoforms. X‐axis represents fold‐change upon UPF3A knockdown (siUPF3A) versus negative control knockdown (siNC) in 3B^Δ2BD^ cells. Number of transcripts in each set (*n*) and *P*‐value from KS test comparing the two distributions are shown on each plot.JBar plot showing average fold change as measured by isoform specific RT‐qPCR of PTC^+^ and PTC^−^ isoform from genes indicated on the bottom in WT and two independent clones of 3A^KO^, 3B^Δ2BD^, 3B^KO^, and 3^DKO^ cells. Relative levels from each replicate are shown by white circles. Error bars indicate standard errors of means. The asterisk (*) represents *P* < 0.05 in *t*‐test with null hypothesis of true mean being 1 (*n* = 3 biological replicates). Source data are available online for this figure.

We next sought to address how can EJC‐dependent NMD operate in human cells in the absence of UPF3B, which is widely believed to act as a bridge between the UPF proteins and the downstream EJC. One possibility is that UPF3A, which can associate with both UPF2 and EJC (albeit weakly) (Kunz *et al*, 2006; Chan *et al*, 2009), may serve such a function. We noted that as previously observed (Tarpey *et al*, 2007; Chan *et al*, 2009), UPF3A is upregulated ~3.5‐fold in UPF3B mutant cells (Fig EV2A; not readily evident in Fig 2A where UPF3A was probed with a different antibody that yields a co‐migrating cross‐reacting band). Notably, in RNA‐Seq data from 3B^Δ2BD^ cells, *UPF3A* mRNA shows a 1.8‐fold increase. Thus, the overall increase of UPF3A in UPF3B mutant cells likely occurs both at the mRNA and at the protein level. To investigate UPF3A association with NMD complexes in the presence or absence of fully functional UPF3B, we performed immunoprecipitation (IP) of UPF1 that was FLAG affinity tagged at its endogenous locus in WT, 3B^Δ2BD^ and 3B^KO^ HCT116 cell lines. While in the WT cells, UPF1 mainly associates with UPF3B and only minimally with its paralog UPF3A, in UPF3B mutant cell lines, UPF1‐UPF3A association is enhanced ~4–6‐fold (Fig 2A). Importantly, the enhanced UPF1‐UPF3A association is independent of RNA. Similarly, UPF3A also shows increased co‐IP with core EJC factor EIF4A3 (~2‐fold increase; Fig 2B) and peripheral protein CASC3 (Fig EV2B) in UPF3B mutant cell lines.

To validate that UPF3A is indeed incorporated into the EJC‐UPF complex, we generated UPF3A or UPF3B knockout HCT116 cell lines where a FLAG affinity tag is inserted into the *MAGOH* locus and a MYC affinity tag into the *UPF2* locus. From these cells, a tandem IP of FLAG‐MAGOH followed by MYC‐UPF2 can isolate the EJC‐UPF complex from WT, UPF3A knockout or UPF3B knockout cells. In the WT and UPF3A knockout cells, UPF3B is the major paralog incorporated into the EJC‐UPF complex as indicated by co‐IP of UPF3B and CASC3 (Fig EV2C). In comparison, in the UPF3B knockout cells, UPF3A is incorporated into the complex at a much higher level (Fig EV2C). We also notice that there is an overall decrease in the abundance of the UPF2‐EJC complex in UPF3B knockout cells as compared with WT or UPF3A knockout cells (Fig EV2C). Together, these data suggest that UPF3A is capable of simultaneously engaging with the UPF and EJC proteins, predominantly in the absence of UPF3B.

### UPF3A compensates for UPF3B function in NMD

Previous evidence from human cells suggests that UPF3A can act as a weak NMD activator particularly in cells with reduced UPF3B levels (Kunz *et al*, 2006; Chan *et al*, 2009). Our results above also suggest that UPF3A may provide UPF3 function during NMD in the absence of UPF3B. To evaluate contribution of UPF3A to NMD in wild‐type and UPF3B mutant HCT116 cells, we knocked‐down UPF3A in WT and 3B^Δ2BD^ cells (Fig EV2D) and performed RNA‐Seq to quantify global transcript levels as above. We observe that even though UPF3A knockdown in WT cells leads to widespread changes in the transcriptome (Fig EV2E), only small changes in transcript levels are observed between PTC^+^ and PTC^−^ isoform groups (Fig 2C) and NMD^+^ and NMD^−^ transcripts (Fig 2D). In contrast, we observed a specific and significant upregulation of the PTC^+^ and NMD^+^ transcript groups as compared with their respective control groups in cells depleted of both UPF3A and UPF3B as compared with cells lacking only functional UPF3B (Fig 2E and F). Thus, UPF3A possesses the ability to activate NMD that becomes prominent only in the absence of UPF3B. In wild‐type cells, UPF3A may potentially have a function outside of NMD given its impact on gene expression (Fig EV2E) but not on NMD (Fig 2C and D).

If UPF3A acts as an NMD activator that is redundant to UPF3B, a prediction will be that the NMD‐targeted transcripts will exhibit similar sensitivity to the paralogs. To test this idea, we defined UPF3B‐dependent targets as PTC^+^ transcripts that show significant and ≥ 1.5‐fold upregulation in 3B^Δ2BD^ cells as compared with WT cells, and compared their change upon additional UPF3A knockdown versus control knockdown in 3B^Δ2BD^ cells. As control, we compared change in the corresponding PTC^−^ group under the same conditions. We find that the UPF3B‐dependent PTC^+^ group shows a strong upregulation after UPF3A knockdown in 3B^Δ2BD^ cells (Fig 2G). At the same time, the UPF3B‐independent transcripts, which change ≤ 1.2‐fold in 3B^Δ2BD^ cells as compared with WT cells, are also similarly affected by UPF3A knockdown in 3B^Δ2BD^ cells, albeit to a lesser extent (Fig 2H). We observe a similarly stronger UPF3A sensitivity of the UPF3B‐dependent NMD^+^ transcripts as compared to the UPF3B‐independent NMD^+^ transcripts (Fig 2I). Thus, in the absence of UPF3B, UPF3A acts on a similar set of mRNAs as UPF3B, and NMD targets that are insensitive to UPF3B are only weakly affected by UPF3A.

To further investigate UPF3A function in NMD, we created two independent UPF3A knockout cell lines (3A^KO^) (Fig EV2F and G) and two unique UPF3A+UPF3B double knockout (3^DKO^) cell lines (Fig EV2G). qPCR‐based quantification of PTC^+^ transcripts that are upregulated upon UPF3B loss (Figs 1D and EV1I) and their corresponding PTC^−^ isoforms shows that the loss of UPF3A has minimal or no effect on the abundance of any of the PTC^+^ isoforms (Figs 2J and EV2H) confirming that UPF3A is generally dispensable for NMD of this subset of transcripts in the presence of UPF3B. In comparison, all the PTC^+^ transcripts show the highest upregulation in the 3^DKO^ cell lines (Figs 2J and EV2H) reflecting the additive effects of loss of UPF3A and UPF3B on NMD of these transcripts (except for *NFKBIB*, which shows a minor additive effect). These results confirm that UPF3A activates EJC‐dependent NMD but only in the absence of UPF3B.

**Figure EV3:**
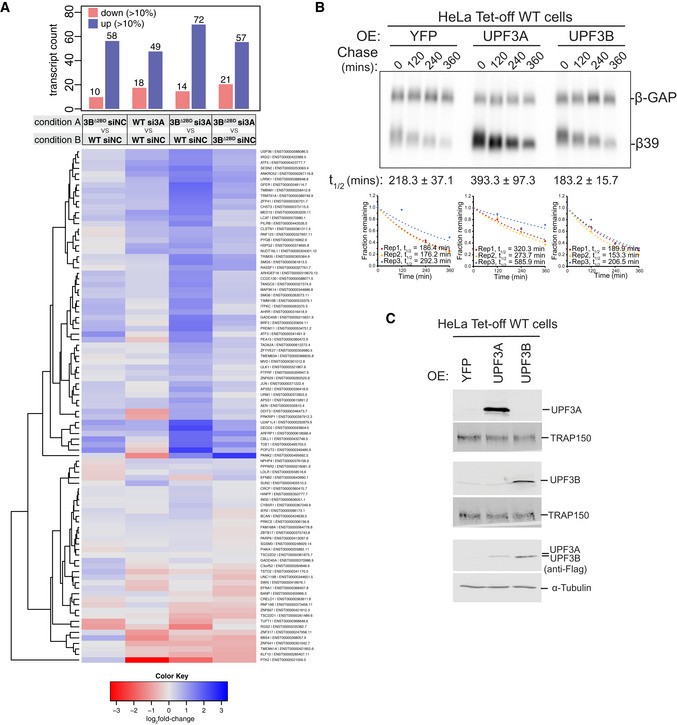
A transcript‐level view of NMD activity of UPF3A Top: Bar plot showing the number of transcripts up‐ or down‐regulated > 10% in conditions labeled on x‐axis. Bottom: Heatmap of “stringent” NMD transcripts and their fold change in different comparisons. A color key for log_2_ fold change values is below the heatmap.Northern blots showing levels of β‐globin reporter mRNAs in wild‐type HeLa Tet‐off cells. β39 is a tetracycline (Tet)‐inducible reporter with a PTC at codon 39 whose levels are shown at different timepoints after transcriptional shut‐off (chase) as indicated above each lane. β‐GAP is a stable, constitutively‐expressed, longer β‐globin mRNA used as transfection control. Proteins overexpressed (OE) in each condition are indicated on top and reporter mRNA half‐lives (t_1/2_) along with standard errors of means are on the bottom of each blot. Below each blot are graphs where fraction of β39 mRNA remaining at various time points in three biological replicates is plotted and fitted to an exponential trendline with y‐intercept set to zero; mRNA half‐lives (t_1/2_) for each replicate obtained from the fit are shown.Protein immunoblots from HeLa Tet‐off cells overexpressing (OE) YFP or human UPF3 proteins as indicated above each lane. TRAP150 and α‐tubulin are used as loading controls.

Previous work has suggested that in mouse and human cells UPF3A can also act as an NMD repressor and its depletion can cause a further downregulation of some NMD targets (Shum *et al*, 2016). However, such an activity of UPF3A is not apparent in HCT116 cells at a global scale when we consider the PTC^+^ and NMD^+^ groups of transcripts (Fig 2C and D). To further evaluate UPF3A’s NMD activator versus repressor activities in our RNA‐Seq datasets, we closely examined a smaller group of NMD^+^ transcripts expressed from genes that can be considered “stringent” NMD regulated targets based on UPF1 sensitivity and phospho‐UPF1 enrichment of their mRNAs in multiple studies (Kurosaki *et al*, 2014; Imamachi *et al*, 2016). Notably, this set contains NMD factor genes, which are known to be autoregulated by NMD (Huang *et al*, 2011; Yepiskoposyan *et al*, 2011). Consistent with our results above with the broader PTC^+^ and NMD^+^ groups, this stringent set of NMD^+^ transcripts also show only modest changes in expression upon UPF3A knockdown (Fig EV3A). While UPF3A knockdown leads to a moderate upregulation of a larger fraction of these transcripts, a smaller set does show a reduction in expression and could include possible targets of NMD repressive activity of UPF3A. However, it is noteworthy that many of these transcripts become upregulated upon UPF3A knockdown in the context of UPF3B inactivation (Fig EV3A). These results are also consistent with our qPCR analyses of several PTC^+^ transcripts that show no reproducible downregulation upon UPF3A loss, either by itself or in combination with UPF3B loss (Figs 2J and EV2H). Thus, while our results do not completely exclude the possibility that UPF3A can act as an NMD repressor for certain NMD targets in HCT116 cells, such an activity in these cells is likely to be an exception rather than a rule. One scenario where we did observe UPF3A to reproducibly repress NMD in human cells is in the case of β‐globin mRNA reporter with a PTC at codon 39 (β39), which is stabilized upon UPF3A overexpression in wild‐type HeLa cells (Fig EV3B and C). In comparison, UPF3B overexpression shows little effect on the reporter mRNA half‐life as compared with the control. Thus, we conclude that while UPF3A generally acts as a weak NMD activator in HCT116 cells, its NMD repressor function can manifest in certain cell‐types and/or conditions such as when the UPF3A:UPF3B ratio is high.

### EJC binding is dispensable for NMD activation by UPF3A and UPF3B

**Figure 3:**
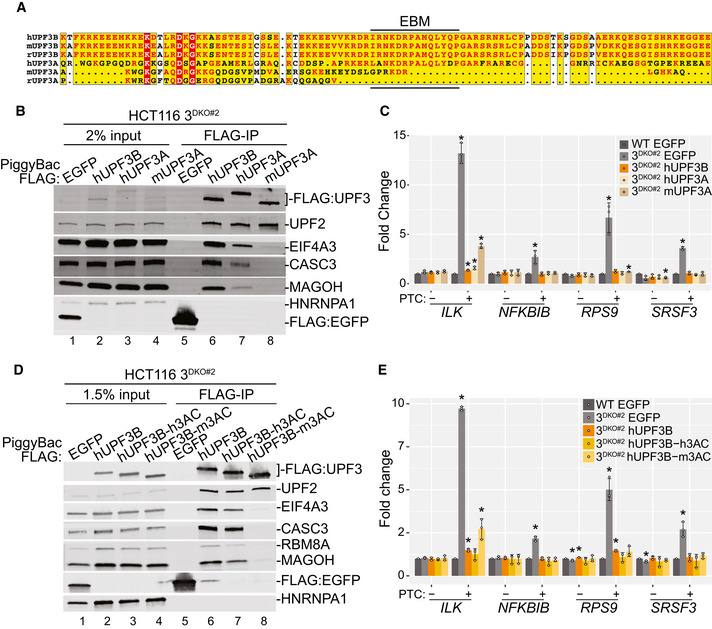
EJC binding is dispensable for NMD activity of UPF3 paralogs Protein sequence alignment of C‐terminal regions of UPF3 proteins from different mammalian species (labeled on the left).Immunoblot showing levels of EJC and UPF proteins (on the right) in input or FLAG‐IP samples from 3^DKO#2^ cells stably expressing different FLAG‐tagged proteins indicated above each lane.Bar plots showing isoform specific RT‐qPCR‐based measurement of relative levels of PTC^+^ and PTC^−^ isoforms of genes indicated below from wild‐type (WT) or 3^DKO#2^ cells stably expressing the specified proteins. Relative levels from each replicate are shown by white circles. Error bars indicate standard errors of means. The asterisk (*) represents statistically significant difference (*P* < 0.05, *t*‐test) of indicated samples from EGFP‐transfected control cells, which has a value of 1 (*n* = 3 biological replicates).Immunoblot showing EJC/UPF proteins (on the right) in input or FLAG‐IP samples from 3^DKO#2^ cells stably expressing FLAG‐tagged proteins given above each lane (hUPF3B = human UPF3B; hUPF3B‐h3AC = human UPF3B protein with C‐terminal domain from human UPF3A; hUPF3B‐m3AC = human UPF3B protein with C‐terminal domain from mouse UPF3A).Bar plots of isoform specific RT‐qPCR‐based analysis of PTC^+^ and PTC^−^ transcript levels from wild‐type (WT) or 3^DKO#2^ cells stably expressing the proteins specified in the legend. Relative levels from each replicate are shown by white circles. Error bars indicate standard errors of means. The asterisk (*) represents statistically significant difference (*P* < 0.05, *t*‐test) of indicated samples from EGFP‐transfected control cells, which has a value of 1 (*n* = 3 biological replicates).

**Figure EV4:**
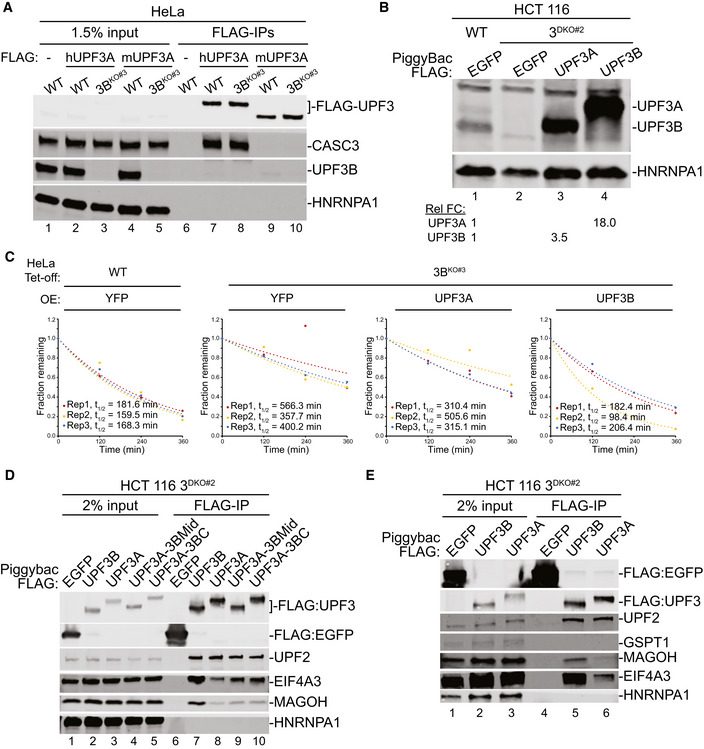
Interactions of UPF3 paralogs and their chimeras with EJC and UPF factors AProtein immunoblots of input and FLAG‐IP from WT and 3B^KO#3^ HeLa cells transiently expressing FLAG‐tagged human or mouse UPF3A as indicated above the lanes. HNRNPA1 is a loading and RNase A digestion control.BProtein immunoblots showing expression levels of FLAG‐tagged UPF3 proteins in WT and 3^DKO#2^ HCT116 cells. HNRNPA1 is used as loading control. Relative fold change (Rel FC) indicates levels of UPF3B (lane 3) and UPF3A (lane 4) expression as compared to levels of their endogenous counterparts in lane 1.CGraphs showing quantification of mRNA decay kinetics for three biological replicates of the assays shown in Fig 4A. Cell lines and overexpressed (OE) protein are indicated on the top. Fraction β39 mRNA remaining at various time points is plotted and fitted to an exponential trendline with y‐intercept set to zero; mRNA half‐lives (t_1/2_) for each replicate obtained from the fit are shown.D, EProtein immunoblots of FLAG‐IP from 3^DKO#2^ cells stably expressing different FLAG‐tagged human UPF3 proteins or their chimeras. HNRNPA1 is used as loading control and RNase A digestion control. FLAG‐EGFP is used as an IP control.

In our attempts to reconcile UPF3A’s (weak) NMD activation ability observed here with its previously reported NMD repressor activity, we discovered that while the carboxy (C)‐terminal domain is conserved among human, mouse, and rat UPF3B homologs, the rodent UPF3A proteins lack most or all residues required for EJC‐binding, whereas human UPF3A retains most of the EJC‐binding residues (Fig 3A). Consistently, IP of FLAG‐tagged mouse UPF3A (mUPF3A) from 3^DKO^ cells shows a near complete absence of EJC factors in the immunoprecipitates (Fig 3B; as compared with human UPF3B, EIF4A3 co‐IP is > 100‐fold reduced with mouse UPF3A). In comparison, human UPF3A can still associate with EJC proteins, albeit weakly as compared with human UPF3B (Fig 3B; 9‐fold reduced EIF4A3 co‐IP with human UPF3A), perhaps due to an arginine‐to‐alanine change at position 423 in the EJC binding motif (EBM) within its C‐terminal domain (Kunz *et al*, 2006). Importantly, all three proteins show a comparable association with UPF2. Furthermore, mUPF3A transiently expressed in HeLa cells also fails to co‐IP any detectable levels of EJC proteins (Fig EV4A). Thus, the UPF3A proteins in mouse, and most likely in rat as well, may have lost their EJC binding ability over the course of evolution. We hypothesized that due to its loss of EJC binding activity, mouse UPF3A will not be able to compensate for *UPF3B* loss in human cells, which might explain the difference between our observations and the previous study that was mainly carried out in mouse cells (Shum *et al*, 2016). Surprisingly, however, when we expressed mouse or human UPF3A proteins, or human UPF3B as a control, in 3^DKO^ cell lines, all three UPF3 proteins fully rescue the NMD of all but one PTC^+^ isoforms we examined (Fig 3C). Mouse UPF3A expression in 3^DKO^ cells leads to partial rescue only in the case of the *ILK* PTC^+^ isoform, which shows the strongest UPF3B dependence (Figs 1D, 2J and 3C) and full rescue by either human UPF3A or UPF3B (Fig 3C). Furthermore, we found that a chimeric human UPF3B, where its C‐terminal domain is replaced by the weaker EJC binding domain of human UPF3A or even the EJC‐interaction deficient C‐terminal domain of mouse UPF3A (Fig 3D), can still retain almost full NMD activity (Fig 3E). These data further confirm that in human cells, the two UPF3 paralogs share NMD activation function, which surprisingly is not dependent on their EJC‐binding ability. Instead, EJC‐UPF3A/3B interaction may play a more secondary role in EJC‐dependent NMD (see below), which can be bypassed at least when UPF3 proteins are expressed in ~3.5‐fold excess in our rescue conditions (Fig EV4B).

### Difference in UPF3A and UPF3B NMD activity is dictated by their mid domains

**Figure 4:**
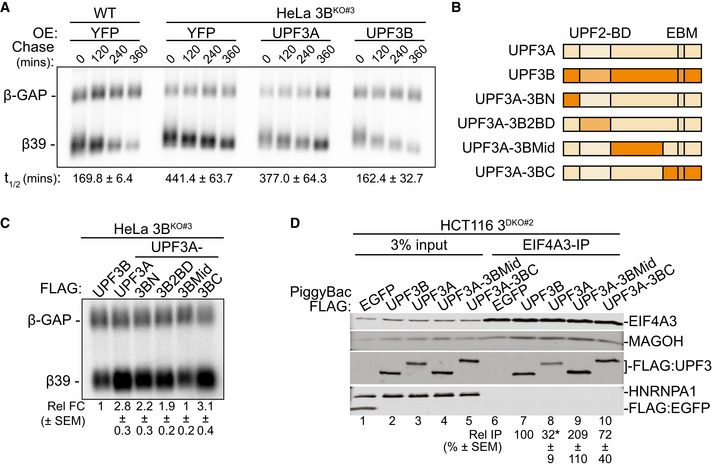
Human UPF3 paralogs differ in NMD activity Northern blots showing levels of β‐globin reporter mRNAs in wild‐type HeLa Tet‐off cells and UPF3B knockout HeLa Tet‐off cells. β39 is a tetracycline (Tet)‐inducible reporter with a PTC at codon 39 whose levels are shown at different timepoints after transcriptional shut‐off (chase) as indicated above each lane. β‐GAP is a stable, constitutively‐expressed, longer β‐globin mRNA used as transfection control. Proteins overexpressed (OE) in each condition are indicated on top and reporter mRNA half‐lives (t_1/2_) along with standard errors of means are on the bottom.Schematic of human UPF3A, UPF3B, and the UPF3A chimeric proteins where UPF3A domains are replaced by the corresponding domains from UPF3B (see material and methods for detailed domain definition). Previously characterized UPF2 binding domain (UPF2‐BD) and EJC‐binding motif (EBM) are shown.Northern blot showing steady‐state levels of β39 NMD reporter and β‐GAP control in HeLa Tet‐off UPF3B knockout cells upon transient overexpression of wild‐type UPF3 proteins or different UPF3A chimeric proteins indicated above each lane. Below each lane, relative fold change (Rel FC) indicates β39 reporter levels (normalized to β‐GAP control) as compared to the normalized β39 reporter levels in UPF3B expressing cells.Immunoblot showing levels of EJC proteins or HNRNPA1 in input or EIF4A3‐IP from 3^DKO#2^ cells stably expressing different UPF3 proteins or EGFP as a control as indicated above each lane. Relative IP of FLAG‐tagged proteins are quantified against EIF4A3.

While our results thus far suggest that UPF3A largely compensates for UPF3B function in NMD, UPF3A’s ability to inhibit NMD when overexpressed in HeLa cells (Fig EV3B) suggested to us that the two paralogs likely have notable differences in their NMD activity. Such a difference is further underscored by only a partial rescue of β39 reporter mRNA NMD in UPF3B knockout HeLa cells upon exogenous UPF3A overexpression (Figs 4A and EV4C) as compared to the full rescue of the reporter mRNA decay by similar UPF3B expression. Our results above (Fig 3) suggest that the weaker EJC binding by UPF3A is unlikely to explain such a difference. So, we sought to identify the molecular basis of the differences in the NMD activity of the human UPF3 paralogs. We created a series of domain swap mutants where each UPF3A domain is replaced by the corresponding sequence from UPF3B (Fig 4B) with the goal to identify the UPF3B domain that will confer UPF3A with full UPF3B‐like NMD activity. As expected, based on our results above, the UPF3B knockout cells overexpressing a UPF3A chimera with the stronger EJC binding UPF3B C‐terminal domain shows similar steady‐state β39 mRNA levels as the cells transfected with wild‐type UPF3A, which is ~3‐fold higher than the cells expressing UPF3B (Fig 4C). Surprisingly, the UPF3A mutant that carries the UPF3B mid‐domain (region between the UPF2 binding domain and the C‐terminal domain) lowers the β39 mRNA to the levels observed with UPF3B rescue (Fig 4C). Thus, the UPF3B mid‐domain, and not its EBM‐containing C‐terminal domain, might underlie the difference between UPF3A and UPF3B NMD activity.

We compared the EJC and UPF binding ability of the UPF3A swap mutants to the wild‐type UPF3 paralogs by stably expressing FLAG‐tagged UPF3 proteins and the UPF3A swap mutants in the UPF3 double knockout background. From these cells, UPF3A shows ~3‐fold weaker co‐IP with EIF4A3 as compared with UPF3B (Fig 4D, compare lanes 7 and 8). Interestingly, both UPF3A‐3BMid and UPF3A‐3BC mutants show an increased association with the EJC (Figs 4D and EV4D) even though only the UPF3A‐3BMid mutant can rescue the decay of NMD reporter mRNA (Fig 4C). It has been previously reported that UPF3B can interact with eRF3 (GSPT1) in cell extracts or *in vitro* even in the absence of the other two UPF factors, and that the UPF3B mid‐domain (smaller than as defined here) can associate directly with eRF3 (Neu‐Yilik *et al*, 2017). However, unlike the previous findings, FLAG‐UPF3A or ‐UPF3B fails to co‐IP detectable eRF3 (Fig EV4E). Thus, we conclude that while the UPF3B mid and C‐terminal domains can independently enhance EJC association, possibly via distinct mechanisms, the difference in NMD activity of UPF3A and UPF3B likely stems from their mid‐domains via a mechanism that remains to be fully understood.

### Some NMD can occur independently of both UPF3 paralogs

**Figure EV5:**
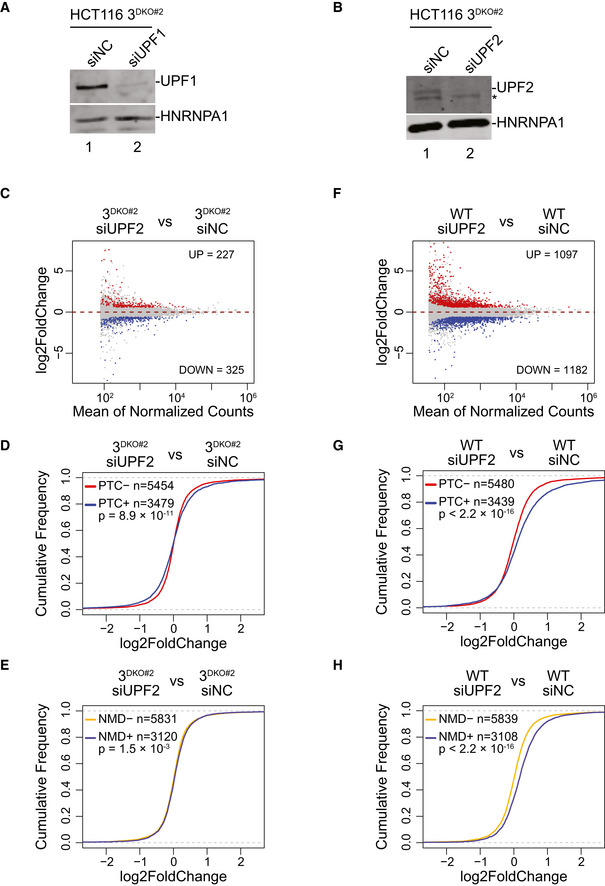
UPF1/UPF2 sensitivity of UPF3A/3B‐dependent NMD A, BImmunoblots showing levels of (A) UPF1, and (B) UPF2 proteins in 3^DKO#2^ cells that were transfected with negative control (siNC), (A) UPF1‐targeting (siUPF1), or (B) UPF2‐targeting (siUPF2) siRNAs. HNRNPA1 is a loading control.CMA plots showing transcript‐level changes upon UPF2 (siUPF2) knockdown as compared to control knockdown (siNC) in 3^DKO#2^ cells. Each dot represents one transcript with average read counts on the x‐axis and log_2_ fold change on the y‐axis. Red and blue dots represent transcripts up‐ or down‐ regulated more than 1.5‐fold with an adjusted *P*‐value < 0.05; counts are shown on each plot.DCumulative Distribution Function (CDF) plots of PTC^+^ isoforms and PTC^−^ isoforms from same set of genes. X‐axis represents fold change in UPF2‐KD (siUPF2) versus control knockdown (siNC) in 3^DKO#2^ cells. Number of transcripts in each set (*n*) and *P*‐value from Kolmogorov‐Smirnov (KS) test comparing the two distributions are shown.ECDF plots of NMD^+^ isoforms and NMD^−^ isoforms. X‐axis represents fold change in UPF2‐KD (siUPF2) versus control knockdown (siNC) in 3^DKO#2^ cells. Number of transcripts in each set (*n*) and *P*‐value from Kolmogorov‐Smirnov (KS) test comparing the two distributions are shown.FMA plots showing transcript‐level changes upon UPF2 (siUPF2) knockdown as compared to control knockdown (siNC) in WT cells. Each dot represents one transcript with average read counts on the x‐axis and log_2_ fold change on the y‐axis. Red and blue dots represent transcripts up‐ or down‐ regulated more than 1.5‐fold with an adjusted *P*‐value < 0.05; counts are shown on each plot.GCDF plots of PTC^+^ isoforms and PTC^−^ isoforms from same set of genes. X‐axis represents fold change in UPF2‐KD (siUPF2) versus control knockdown (siNC) in WT cells. Number of transcripts in each set (*n*) and *P*‐value from Kolmogorov‐Smirnov (KS) test comparing the two distributions are shown.HCDF plots of NMD^+^ isoforms and NMD^−^ isoforms. X‐axis represents fold change in UPF2‐KD (siUPF2) versus control knockdown (siNC) in WT cells. Number of transcripts in each set (*n*) and *P*‐value from Kolmogorov–Smirnov (KS) test comparing the two distributions are shown. Source data are available online for this figure.

**Figure 5:**
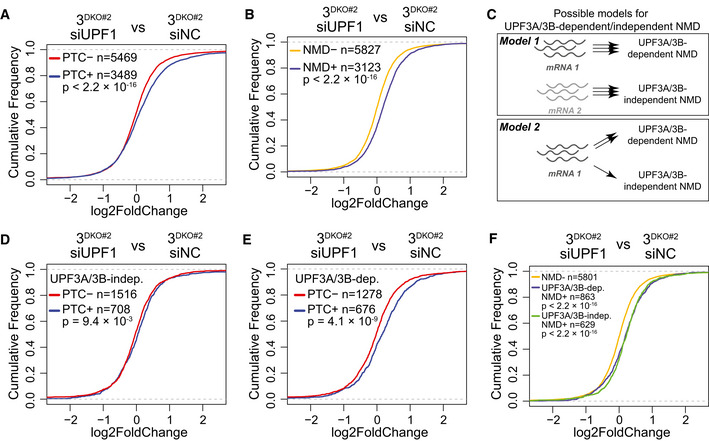
NMD activity in human cells in the absence of both UPF3 paralogs A, BCDF plots of (A) PTC^+^ and PTC^−^ isoforms, (B) NMD^+^ and NMD^−^ isoforms. X‐axis represents fold change upon UPF1 knockdown (siUPF1) versus negative control knockdown (siNC) in 3^DKO#2^ cells. Number of transcripts in each set (*n*) and *P*‐value from KS test comparing the two distributions are shown on each plot.CTwo possible models for UPF3A/3B‐dependent and ‐independent NMD. Model 1 predicts that mRNAs (represented by wavy lines) from one set of genes (mRNA 1) undergoes UPF3A/3B‐dependent NMD and those from another set of genes (mRNA 2) are targeted by UPF3A/3B‐independent NMD. Model 2 posits that same mRNAs are targeted to both UPF3A/3B‐dependent and ‐independent NMD. The number of arrows signify the probability/rate of NMD commitment via each branch. In Model 2, commitment to NMD is higher in the presence of UPF3 paralogs and lower in their absence.D, ECDF plots of UPF3A/3B‐independent (UPF3A/3B‐indep., D) and ‐dependent (UPF3A/3B‐dep., E) PTC^+^ isoforms and their respective PTC^−^ isoforms. X‐axis represents fold change upon UPF1 knockdown (siUPF1) versus negative control knockdown (siNC) in 3^DKO#2^ cells. Number of transcripts in each set (*n*) and *P*‐value from KS test comparing the two distributions are shown on each plot.FCDF plots of UPF3A/3B‐dependent (UPF3A/3B‐dep.), UPF3A/3B‐independent (UPF3A/3B‐indep.) NMD^+^ isoforms and NMD^−^ isoforms. X‐axis represents fold change upon UPF1 knockdown (siUPF1) versus negative control knockdown (siNC) in 3^DKO#2^ cells. Number of transcripts in each set (*n*) and *P*‐value from KS test comparing the two distributions are shown on each plot. Source data are available online for this figure.

Like our surprising finding that EJC‐UPF3A/3B interaction is dispensable for NMD, other previous observations argue that UPF3 proteins are not necessary to bridge the UPF complex to EJC during NMD: TCR‐β reporter mRNA and a handful of endogenous NMD substrates can undergo NMD independently of UPF3 proteins in human cells (Chan *et al*, 2007; Karam *et al*, 2015). However, in these experiments, both UPF3 paralogs were depleted using RNA interference thus leaving open a possibility that some residual UPF3A/3B proteins may still be able to provide the bridging function to sustain NMD. Using 3^DKO^ cells, we tested the existence of any NMD that can still occur in complete absence of the UPF3 paralogs. Like in wild‐type cells (Fig EV1H), UPF1 knockdown in 3^DKO^ cells (Fig EV5A) leads to further upregulation of PTC^+^ isoforms compared with PTC^−^ isoforms (Fig 5A) and NMD^+^ transcripts as compared with NMD^−^ transcripts (Fig 5B). Thus, some NMD can still function in the complete absence of both UPF3 proteins and hence their UPF‐EJC bridging function.

Our data in Fig 5A and B provide strong support for the existence of a UPF3A/3B‐independent NMD branch. We next evaluated the target specificity of the UPF3A/3B‐dependent and ‐independent branches. Two possible models of UPF3A/3B dependence and independence of NMD‐targeted mRNAs are shown in Fig 5C. In model 1 (Fig 5C, top), mRNAs from one set of genes need UPF3A/3B for their NMD and would constitute targets of UPF3A/3B‐dependent NMD. mRNAs from a different gene set can undergo NMD in absence of UPF3A/3B and would represent targets of UPF3A/3B‐independent NMD. In an alternative model 2 (Fig 5C, bottom), the same mRNAs can be targeted to UPF3A/3B‐dependent and UPF3A/3B‐independent NMD. In the presence of UPF3A/3B, these mRNAs are most efficiently suppressed by UPF3A/3B‐dependent NMD. In the absence of UPF3A/3B, these mRNAs can still undergo NMD albeit less efficiently via a UPF3A/3B‐independent mechanism. To test these models, we first separated PTC^+^ mRNAs into two groups (i) a “UPF3A/3B‐dependent” group that is significantly upregulated ≥ 1.5‐fold in 3^DKO^ cells, and (ii) a “UPF3A/3B‐independent” group that changes ≤ 1.2‐fold in either direction in 3^DKO^ cells. The UPF3A/3B‐independent group is expected to contain distinct mRNAs that are targeted only by the UPF3A/3B‐independent NMD branch and are still undergoing efficient NMD in 3^DKO^ cells. Such PTC^+^ mRNAs are expected to be upregulated upon UPF1 knockdown in 3^DKO^ cells. However, we observe that the UPF3A/3B‐independent PTC^+^ mRNA group shows only a very minor upregulation as compared with their PTC^−^ counterparts when 3^DKO^ cells are depleted of UPF1 (Fig 5D). In contrast, the UPF3A/3B‐dependent group of PTC^+^ mRNAs shows a prominent upregulation upon UPF1 knockdown in 3^DKO^ cells (Fig 5E). These data indicate that PTC^+^ mRNAs (i.e., potential EJC‐dependent NMD targets) that undergo UPF3A/3B‐dependent NMD can still be targeted by the NMD pathway in UPF3A/3B‐independent manner, a conclusion that fits model 2. Interestingly, a similar analysis where we split NMD^+^ transcripts into UPF3A/3B‐dependent and ‐independent groups shows that both groups are similarly and significantly upregulated upon additional UPF1 depletion in 3^DKO^ cells (Fig 5F). Thus, among NMD^+^ transcripts, the UPF3A/3B‐dependent group of transcripts can undergo NMD in both UPF3A/3B‐dependent and ‐independent manner (conformant to model 2), whereas the UPF3A/3B‐independent group is truly independent of UPF3 paralogs for their NMD (conformant to model 1). We conclude that in HCT116 cells, some NMD‐targeted mRNAs can completely bypass the need for UPF3 paralogs, whereas other mRNAs can undergo NMD at a variable efficiency depending on UPF3A/3B levels.

We also tested the UPF2 dependence of UPF3A/3B‐dependent and ‐independent transcripts. We find that unlike UPF1 depletion, UPF2 knockdown in 3^DKO^ cells (Fig EV5B) causes a negligible change in PTC^+^ or NMD^+^ transcripts as compared with their respective control groups (Fig EV5C–E). In contrast, both of these classes of NMD targets are robustly upregulated upon UPF2 knockdown in WT cells (Fig EV5F–H). Thus, in HCT116 cells, UPF3A/3B‐dependent NMD targets are also likely to be reliant on UPF2.

### CASC3‐containing EJC enhances NMD by recruiting UPF3 to pre‐translation mRNPs

**Figure 6:**
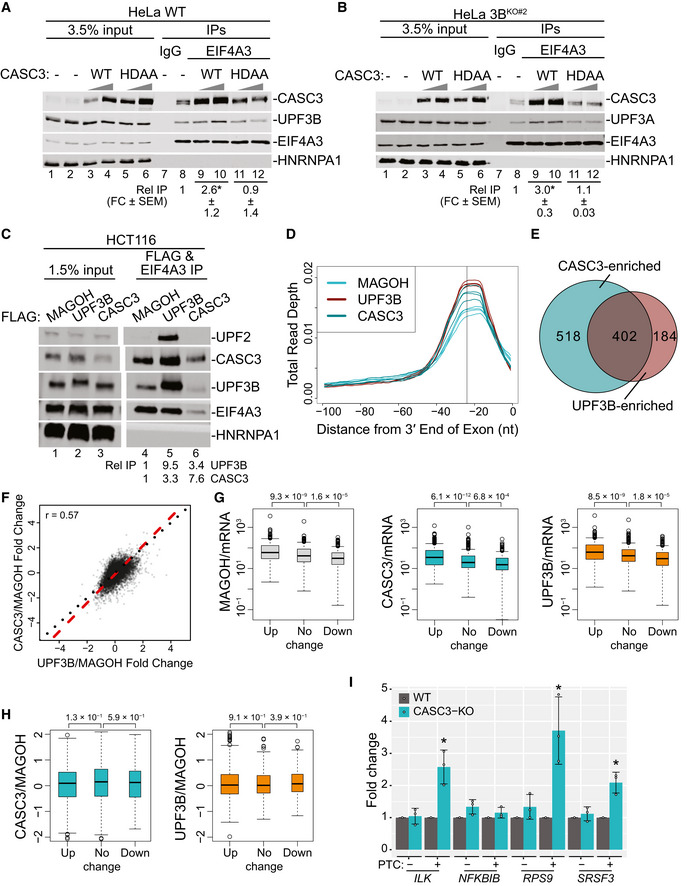
CASC3 promote EJC association of UPF3 paralogs to enhance NMD A, BWestern blots showing levels of EJC proteins or HNRNPA1 (control) in input, IgG IP or EIF4A3 IP following overexpression (OE) of CASC3 wild‐type (WT) and EJC binding‐deficient (HDAA) mutant proteins in (A) HeLa Tet‐off cells, and (B) 3B^KO#3^ HeLa Tet‐off cells. Ramps above lanes indicate expression levels of the CASC3 proteins. The asterisk (*) represents *P* < 0.05 in *t*‐test with null hypothesis of true mean less or equal than 1.CWestern blots showing levels of EJC/UPF proteins and HNRNPA1 in input and FLAG followed by EIF4A3 tandem IP from HCT116 cells expressing the FLAG‐tagged endogenous protein indicated above each lane. Quantifications of UPF3B and CASC3 protein enrichment from two replicates are shown at the bottom.DMeta‐exon plot showing read distributions within the 100 nucleotide (nt) window from the exon 3′ end in RIPiT‐Seq replicates of MAGOH:EIF4A3, UPF3B:EIF4A3, and CASC3:EIF4A3. The black vertical line indicates the −24 nt position.EVenn diagram showing the degree of overlap between genes significantly enriched in CASC3:EIF4A3 EJC and UPF3B:EIF4A3 EJC occupancy as compared to MAGOH:EIF4A3 EJC occupancy.FScatter plot comparing log_2_‐transformed fold change in occupancy of CASC3:EIF4A3 EJC as compared to MAGOH:EIF4A3 EJC (x‐axis) and UPF3B:EIF4A3 EJC compared to MAGOH:EIF4A3 EJC. Each dot represents a gene where gene‐level occupancy of each EJC composition was quantified at the canonical position for EJC footprints. Pearson’s correlation coefficient is shown on the top left. The black dotted line indicates the x = y diagonal and the red dashed line indicates the total least square regression fit for the data points.GBox plots comparing occupancy of a specific EJC composition on mRNA (indicated on y‐axis; normalized by gene expression from RNA‐Seq) between genes of different NMD efficiency. Up, no, and down stands for upregulation, no obvious change or downregulation of a representative PTC^+^ isoform for each gene (see material and methods) that is used to reflect overall NMD efficiency for each gene. In the box plots, central band represents the median, boxes represent the interquartile range (IQR), whiskers indicate maximum (top) and minimum (bottom) values (1.5‐times the highest or lowest IQR values, respectively), and circles represent the outliers. *P*‐values are from Wilcoxon signed‐rank test comparing the two indicated distributions.HBox plots as in (G) comparing specific EJC composition occupancy relative to EJC core occupancy (normalized by MAGOH:EIF4A3 RIPiT‐Seq) between genes of different NMD efficiency. Up, no, down, central band, boxes, and whiskers are as in (G).IBar plots showing fold changes measured by isoform specific RT‐qPCR of PTC^+^ and PTC^−^ isoform from genes indicated on the bottom in WT and CASC3‐KO HCT116 cells. Relative levels from each replicate are shown by white circles. Error bars indicate standard error of means. The asterisk (*) represents *P* < 0.05 in *t*‐test with null hypothesis of true mean being 1 (*n* = 3 biological replicates). Source data are available online for this figure.

Our results raise an important question regarding the function of the EJC‐UPF3 interaction, which is well‐conserved in multicellular eukaryotes and is consequential for human NMD (Gehring *et al*, 2003; Buchwald *et al*, 2010). We examined the possibility that interaction with the EJC may boost UPF3 function in NMD by recruiting these proteins to mRNA‐containing ribonucleoproteins (mRNPs). Note that EJC‐bound mRNAs represent pre‐translation mRNPs as the EJC is disassembled from RNA as the first ribosome transits through the mRNA (Dostie & Dreyfuss, 2002). We particularly focused on EJCs containing CASC3, the peripheral EJC factor that can promote the UPF3B‐EJC interaction (Gerbracht *et al*, 2020). Consistent with the previous work, we observe an enhanced EJC‐UPF3B association (2.6‐fold) in HeLa cells upon overexpression of wild‐type CASC3 but not the CASC3 HDAA mutant that is unable to associate with the EJC core (Ballut *et al*, 2005) (Fig 6A, compare lanes 9–10 with lanes 11–12). Furthermore, in UPF3B knockout HeLa cells (Appendix Fig S1A–C), a similar overexpression of wild‐type CASC3 but not the CASC3 HDAA mutant enhances UPF3A co‐IP with EIF4A3 by 3‐fold (Fig 6B, compare lanes 9–10 with lanes 11–12). Conversely, CASC3 knockdown reduces EJC‐UPF3B association in wild‐type HeLa cells and EJC‐UPF3A association in UPF3B knockout HeLa cells (Appendix Fig S1D). Together, these results suggest that CASC3 promotes EJC association of both UPF3 paralogs.

We hypothesized that preferential association between CASC3 and UPF3B will enrich the EJCs containing the two proteins on a similar set of transcripts. To test this possibility, we carried out RIPiT‐Seq (RNA IP in tandem followed by high‐throughput sequencing (Singh *et al*, 2012)) from HCT116 cells that express FLAG‐tagged fusions of UPF3B, CASC3, and MAGOH from their endogenous loci. From these cells, FLAG IPs followed by a second IP of the EJC core factor EIF4A3 purifies compositionally different EJCs. We find that CASC3 is ~3‐fold enriched in UPF3B‐containing EJCs as compared with EJCs purified via MAGOH and EIF4A3, which likely yields a mixture of different EJC compositions (Fig 6C, compare lanes 4 and 5). Furthermore, as expected, the RNA footprints of FLAG‐MAGOH:EIF4A3, FLAG‐UPF3B:EIF4A3, and FLAG‐CASC3:EIF4A3 complexes show a strong enrichment at the exon 3′ ends at the main EJC binding site (Fig 6D and Appendix Fig S1E). To test if UPF3B and CASC3 exhibit synergistic binding to transcripts, we first individually compared CASC3:EIF4A3 and UPF3B:EIF4A3 occupancy to the EJC core (MAGOH:EIF4A3) occupancy. Genes that are enriched in either UPF3B‐EJC or CASC3‐EJC as compared with the EJC core show a large and significant overlap (Fig 6E). In contrast, little overlap exists between genes that are UPF3B‐depleted and CASC3‐enriched, or vice versa (Appendix Fig S1F and G). We observe a strong positive correlation between transcriptome‐wide UPF3B and CASC3 binding relative to the EJC core (Fig 6F) suggesting that UPF3B and CASC3 preferentially bind to a similar set of transcripts.

We next asked if increased EJC and/or UPF3B binding to an mRNA leads to more efficient NMD. For this analysis, we selected genes that express at least one PTC^+^ isoform, and for NMD efficiency of each such gene, we used the highest fold change observed for any of its PTC^+^ isoforms in UPF3A/3B depleted cells (UPF3A knockdown in 3B^Δ2BD^ cells) as compared with control (WT cells with negative control knockdown). A comparison of NMD efficiency estimates for these genes to their expression‐normalized EJC occupancy reveals that genes that express PTC^+^ isoforms with highest UPF3A/3B sensitivity (> 1.5‐fold change) show the highest occupancy for EJC core, CASC3 and UPF3B (Fig 6G) as compared with the genes that express PTC^+^ isoforms that remain unchanged (≤ 1.2‐fold change in either direction) or are downregulated (< 1.5‐fold change) in these conditions. Such a difference is, however, not observed among the three groups of genes when CASC3 or UPF3B occupancy is normalized to EJC core occupancy (Fig 6H). These findings suggest that NMD sensitivity of a transcript is weakly but appreciably affected by the EJC core occupancy, which also possibly dictates CASC3 and UPF3A/3B occupancy within mRNPs.

To directly test if CASC3 can influence UPF3A/3B‐dependent NMD, we measured the levels of UPF3A/3B‐dependent PTC^+^ transcripts in HCT116 cells where CASC3 expression is completely knocked out by frameshifting indels around its start codon (Appendix Fig S1H). In these cells, *ILK*, *RPS9,* and *SRSF3* PTC^+^ transcripts show a moderate increase in abundance (Fig 6I). No such change is seen in the case of the *NFKBIB* PTC^+^ isoform (Fig 6I), an NMD target that is the least affected by the loss of UPF3A/3B (Fig 2J). Thus, normal CASC3 levels boost UPF3A/3B‐dependent NMD possibly through increased recruitment of UPF3 paralogs to EJC‐bound pre‐translation mRNPs.

## Discussion

**Figure 7:**
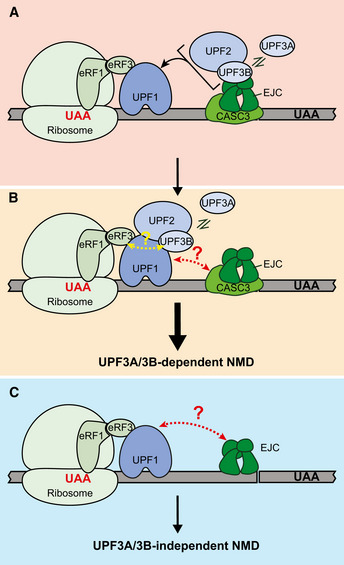
A proposed model for function of UPF3 paralogs in EJC‐enhanced NMD In UPF3 paralog‐dependent NMD, prior to UPF1 activation, CASC3‐EJC enhances the presence of UPF3B and UPF2 on exon‐exon junctions. UPF3A can replace UPF3B when UPF3B levels are insufficient. Black arrow indicates that such enhanced UPF3 paralog and UPF2 recruitment to mRNA via EJC can later facilitate their assembly with UPF1 to form the NMD complex.Within the UPF complex that senses aberrant translation termination and activates NMD, UPF3 paralogs perform a function that is independent of its EJC binding. Perhaps, such a function involves their interaction with eRF3 (indicated by a dotted yellow line; Neu‐Yilik *et al*, 2017). The EJC still plays a more direct role during this NMD activation step (indicated by a red dotted line with a question mark), such a function likely occurs independently of EJC‐UPF3A/3B interaction. These events culminate in NMD in UPF3A/3B‐dependent manner, which occurs at a higher probability than in absence of UPF3 paralogs (signified by a higher weight arrow).NMD can occur in UPF3A/3B‐independent manner. It remains possible that the EJC can still communicate with the premature termination complex in a UPF3A/3B‐, and possibly UPF2‐independent manner (indicated by a red dotted line with a question mark) to elicit NMD. mRNAs are targeted to NMD at a lower probability in the absence of UPF3 paralogs (signified by a lower weight arrow).

Of the three core NMD factors, UPF3 has evolved most rapidly in eukaryotes. In multicellular organisms, it appears to have lost its essentiality for NMD activity while gaining the ability to interact with the NMD‐stimulating EJC. Furthermore, in vertebrates, the *UPF3* gene has duplicated into paralogous *UPF3A* and *UPF3B*, which have diverged in their EJC binding ability. The emergence of these variations in *UPF3* raises several questions: What is UPF3’s primary function in NMD? If NMD can occur without the EJC in yeast, what is the role of EJC interaction in UPF3 function? How do the two paralogs contribute to UPF3 activity in the pathway? How can NMD function in the absence of UPF3B, or UPF3 activity altogether, and how prevalent is such NMD? Our work here using UPF3A and UPF3B loss‐of‐function human cell lines addresses these questions, leading to an updated model of UPF3A and UPF3B function in mammalian NMD (Fig 7), which is further elaborated below.

### UPF3A function in NMD

The early studies on UPF3A suggested that it acts as a weak NMD activator in human cells (Kunz *et al*, 2006; Chan *et al*, 2009). While confirming such a function, the recent work by Shum *et al* (2016) using mouse models and cell lines has suggested that the UPF3 gene duplication fueled neo‐functionalization of UPF3A that also makes it an NMD repressor. In a model proposed by Shum *et al* (2016), weak EJC binding by UPF3A sequesters UPF2 away from the NMD complex thereby leading to NMD inhibition. In our work here in human (HCT116) cells, we do not observe any widespread downregulation of NMD targets when UPF3A is depleted via RNA interference (Figs 2C and D, and EV3A) or when it is completely knocked out (Figs 2J and EV2H) in wild‐type cells. These data suggest that in these cells UPF3A does not interfere with UPF3B function during NMD, and hence does not act as an NMD repressor. On the contrary, several lines of evidence from our work suggests that UPF3A acts as an NMD activator (Fig 7A and B), particularly in the absence of UPF3B. In cells lacking UPF3B, UPF3A is upregulated (Fig EV2A) (Tarpey *et al*, 2007; Chan *et al*, 2009; Nguyen *et al*, 2012), and its incorporation in EJC‐UPF complexes is dramatically enhanced (Figs 2A and B, and EV2B and C) (Chan *et al*, 2009). Furthermore, a partial or complete UPF3A depletion in UPF3B lacking cells leads to robust upregulation of NMD targets at a global level and at an individual transcript level (Figs 2E, F, and J, EV2H, and EV3A). This evidence suggests that in the absence of UPF3B, UPF3A engages with the NMD machinery to sustain the EJC‐dependent and other NMD branches. Moreover, we find that UPF3A is comparable to UPF3B in its ability to rescue EJC‐dependent NMD of various endogenous PTC‐containing mRNAs (Fig 3C) although some notable differences between the two paralogs are observed (see below). Importantly, UPF3A’s impact on NMD shows a remarkable overlap with that of UPF3B (Fig 2G and I). A parallel study by Wallmeroth *et al* also shows that UPF3A functions as a NMD activator in HEK293 cells that lack UPF3B. The redundancy between UPF3A and UPF3B is more pronounced in these cells as NMD inhibition is observed only upon depletion of both proteins (Wallmeroth *et al*, 2022). Thus, in human patients with UPF3B inactivating mutations, UPF3A can likely fill in for UPF3B during NMD, which is critical for several physiological processes (e.g., hematopoiesis (Weischenfeldt *et al*, 2008)). However, it is clear that UPF3A cannot always compensate for UPF3B (e.g., during brain development) possibly due to differences among the paralogs in their function or gene expression patterns.

While our findings point to an NMD activating role for UPF3A, in certain contexts, UPF3A can act as NMD repressor. For example, when UPF3A is overexpressed in wild‐type HeLa cells, NMD of the β‐globin reporter mRNA slows down (Fig EV3B) (Chan *et al*, 2009). Such conditions do arise in certain cell types (e.g., mouse germ cells) and/or developmental stages (e.g., early mouse embryogenesis and spermatogenesis) where UPF3A expression is dramatically increased. An important context is male germ cells where UPF3A is likely the main source of UPF3 activity due to the presumed silencing of *UPF3B* due to meiotic X‐chromosome inactivation (Turner, 2007). In these cells, high UPF3A to UPF3B ratio inhibits NMD of select tested targets. (Shum *et al*, 2016). As NMD activity is known to vary by cell‐types (Huang *et al*, 2011), it is possible that UPF3A repressor activity may be more prominent in certain cell types (e.g., mouse P19 cells used in Shum *et al*). We also cannot completely rule out that UPF3A functions as a NMD repressor for a select set of NMD regulated transcripts in HCT116 cells that are downregulated upon UPF3A knockdown (Fig EV3A).

More work in the future will be necessary to resolve if the differences in NMD activating versus repressive abilities of UPF3A observed in various studies stem from the use of transformed cell lines (our work and (Kunz *et al*, 2006; Chan *et al*, 2009; Wallmeroth *et al*, 2022)) versus primary cells (Shum *et al*, 2016). It is important to note that UPF3A function as an NMD repressor in various primary cells (neural stem cells, embryonic fibroblasts, spermatocytes, olfactory sensory precursor cells and neurons) was described based on only a handful of NMD targets (Shum *et al*, 2016). The observed differences could also be due to human versus mouse specific differences in UPF3A proteins and their functions (Fig 3). Therefore, it will be important to assess UPF3A function in NMD at a global scale in multiple contexts (different organisms, cell types, tissues, developmental stages etc.) to determine whether NMD repression is its dominant function, or if it acts both as NMD activator and as repressor perhaps in a transcript specific manner as observed in P19 cells, or if it primarily acts as an NMD activator as in HCT116 cells. A better understanding of the extent to which UPF3A functions synergize or antagonize those of UPF3B is also necessary to understand the basis of UPF3B‐caused human neurological disorders. An exhaustive analysis of UPF3A will also provide a clearer picture of how its various functions—NMD activation, NMD repression, and/or functions outside NMD (Ma *et al*, 2019)—dictated its co‐existence with UPF3B after *UPF3* gene duplication during early vertebrate evolution ~500 million years ago.

### A revised model for UPF3A/3B function in NMD

Current NMD models suggest that in EJC‐dependent NMD, UPF3B acts as a bridging molecule between the UPF proteins and the downstream EJC (see Introduction). Surprisingly, our data show that mouse UPF3A, which is missing most of the EJC binding motif (Fig 3A) and hence lacks any detectable EJC binding (Fig 3B), and human UPF3B with the EJC binding‐deficient domain from mouse UPF3A (Fig 3D), are still capable of rescuing NMD of several PTC‐containing mRNAs (Fig 3C and E). Additionally, replacing the weaker EJC binding C‐terminal region of human UPF3A with the stronger EJC binding C‐terminal domain of human UPF3B does not improve the NMD function of the chimeric UPF3A protein (Fig 4C and D). Thus, UPF3A/3B can activate NMD without EJC binding, thereby challenging the decades‐old bridging model for function of UPF3 proteins in the pathway. Our conclusion is further bolstered by the findings of Wallmeroth *et al* where a complete deletion of UPF3B C‐terminal domain can still support functional NMD (Wallmeroth *et al*, 2022).

We propose that EJC binding by UPF3 proteins is not a primary activity of these proteins in the NMD pathway. EJC binding is perhaps important to recruit UPF3A/3B to mRNA exon‐exon junctions (Figs 6 and 7A) to increase the likelihood of NMD activation by yet another UPF3A/3B function when translation terminates at PTCs (Fig 7B). The recruitment of both human UPF3 paralogs to mRNA via the EJC can be enhanced by CASC3 (Figs 6, 7A, and Appendix Fig S1), which is a defining component of a compositionally distinct EJC (Mabin *et al*, 2018). It is possible that additional interactions between N‐ or C‐terminal domains of CASC3 on either side of its EJC binding SELOR domain (Buchwald *et al*, 2010; Melero *et al*, 2012) and UPF3 proteins (e.g., mid‐domain, which enhances EJC interaction (Fig 4D)) contribute to EJC association of UPF3 paralogs. Possibly, modulation of CASC3 levels, for example by miR128 in neuronal cells (Bruno *et al*, 2011), can regulate UPF3A/3B‐dependent NMD. How can the need for UPF3A/3B recruitment to mRNAs via the EJC be bypassed? It is possible that at higher UPF3A/3B expression levels, such as those achieved in the rescue experiments in Figs 3 and 4, this prior mRNA recruitment of UPF3 paralogs becomes dispensable. Interestingly, compromised recruitment of UPF3B to EJC in cells depleted of ICE1, which also aids in UPF3B‐EJC interaction, can be similarly overcome by UPF3B overexpression (Baird *et al*, 2018).

What is the primary role of UPF3 proteins in NMD activation? Early studies from yeast revealed that UPF proteins, including UPF3, can physically engage with termination factor eRF3 (Wang *et al*, 2001). These data suggested a role for UPF proteins in discrimination between normal and premature termination events, but details of such mechanisms have remained elusive. A recent investigation using an *in vitro* assay to monitor translation termination found that UPF3B, but not UPF1 or UPF2, can slow down the termination reaction and promote disassembly of the terminated ribosome (Neu‐Yilik *et al*, 2017). Interestingly, this report shows that UPF3B can directly interact with eRF3 and eRF1. Indeed, the direct *in vitro* UPF3B‐eRF3 interaction is mediated by a UPF3B region that falls within the segment that we define here as the mid‐domain (Fig 3C). How these translation termination‐linked UPF3B activities and interactions contribute to NMD was, however, unknown. Our work here shows the importance of the mid‐domain for efficient NMD (Fig 4C), even though we do not detect an association between UPF3B and eRF3 in HCT116 cells (Fig EV4E). It is possible that this interaction is transient in nature and is reliably detectable only when the two proteins are exogenously expressed at much higher levels as in the previous work (Neu‐Yilik *et al*, 2017). It also remains to be seen if differences in NMD activity of UPF3A and UPF3B are governed by the differences in their mid‐domains to engage with termination factors. Nonetheless, our data, the findings of Wallmeroth *et al* in the accompanying paper and the published work (Neu‐Yilik *et al*, 2017) indicate that the UPF3A/3B mid‐domain is an important determinant of NMD activity, possibly by engaging with and modulating the translation termination machinery (Fig 7B). The functional relevance of the poorly characterized mid‐domain of the UPF3 proteins is further underscored by several missense *UPF3B* mutations that fall within this domain in individuals with neurodevelopmental disorders (Alrahbeni *et al*, 2015).

It is likely that UPF3B continues to function in the NMD pathway even after termination is deemed to be premature possibly by promoting ribosome dissociation and/or recycling (Neu‐Yilik *et al*, 2017). It will be an important goal for future studies to precisely define the order and mechanism of steps that lead to NMD activation, and the contribution of UPF3 proteins to these steps.

### UPF3A/3B‐dependent and ‐independent NMD branches

Even though UPF3B plays a critical role in activating efficient NMD, multiple lines of evidence suggest that NMD in some organisms can proceed independently of UPF3 proteins (see Introduction, (Yi *et al*, 2021) and references therein). Using human cell lines that completely lack both UPF3A and UPF3B, our work confirms previous observations (Chan *et al*, 2007; Karam *et al*, 2015) and provides definitive evidence for the existence of UPF3A/3B‐independent NMD as large cohorts of NMD‐targeted mRNAs are still repressed by UPF1 in these cells (Fig 5A and B). However, PTC^+^ transcripts do not respond to UPF2 depletion in UPF3 double knockout cells (Fig EV5D and E) suggesting that UPF3 proteins and UPF2 regulate a similar set of transcripts in an inter‐dependent manner, and that EJC‐dependent NMD that is UPF3A/3B‐independent is also likely to be UPF2‐independent. How such NMD could proceed in the absence of UPF3 proteins (and UPF2) (Fig 7C) remains to be investigated.

Intriguingly, we observe that for EJC‐dependent NMD targets, UPF3A/3B‐dependent and ‐independent NMD branches are more likely to reflect fractions of the same mRNAs that commit to NMD, perhaps at variable rates depending on UPF3A/3B presence (Fig 5C–E). Curiously, beyond the EJC‐dependent NMD, there are transcripts that are not affected at all by the loss of UPF3 proteins and thus are true representatives of a UPF3A/3B‐independent NMD branch (Fig 5F). Thus, our data suggest that UPF3 proteins act as non‐essential enhancers of NMD that may function by altering the probability at which a particular mRNA is subjected to NMD. Future work will be necessary to dissect functions of UPF3A/3B in premature termination and NMD activation and why such functions can be expendable for certain transcripts. It is conceivable that transcript features that affect steps during or immediately after translation termination (e.g., ribosome readthrough, and recycling) and/or recruitment/activation of NMD factors such as UPF1 or UPF2 may dictate the degree of dependence of an mRNA on UPF3 paralogs. Interestingly, a genetic screen identified UPF3B as a key factor for NMD of a reporter with a long 3’UTR that uses a readthrough prone stop codon as the PTC but not for NMD of a similar reporter where the PTC is a stop codon that is used as normal termination codon in human mRNAs (Zinshteyn *et al*, 2021). Previous work also has suggested that UPF3A/3B dependence of a particular transcript can change in cell and/or tissue specific manner (Huang *et al*, 2011). Thus, a transcript that depends on UPF3A/3B in one cell type (i.e., targeted by UPF3A/3B‐dependent branch) could reach the threshold for NMD in another cell type in the absence of UPF3 paralogs (i.e., targeted by UPF3A/3B‐independent branch). Therefore, additional factors such as the complement of proteins bound to an mRNA in a particular cell type can influence UPF3A/3B dependence. In addition to the complete dispensability of UPF3 proteins, the redundant nature of individual UPF3B activities like UPF2 or EJC binding (Wallmeroth *et al*, 2022), also raises questions about contexts where such redundant activities of UPF3 proteins become important for NMD activation.

## Materials and Methods

### Plasmids

For CRISPR‐Cas9‐mediated antibiotic resistance marker and polyadenylation signal knock‐in experiments (for gene knockouts), PX330 plasmid was used for introducing Cas9‐mediated cuts. PX330 was a gift from Feng Zhang (Addgene plasmid # 42230; http://n2t.net/addgene:42230; RRID:Addgene_42230). Guide RNA sequence was cloned as previously described (Ran *et al*, 2013). For donor plasmids, 300–500 bp left and right homology region from gene‐of‐interest, and puromycin resistance marker‐Bovine Growth Harmone poly(A)‐signal (amplified from pMK232 (Natsume *et al*, 2016)) or blasticidin resistance marker‐Simian Virus 40 poly(A)‐signal (amplified from pcDNA6/TR (Thermo Fisher)) were cloned into pTwist‐Amp (Twist Bioscience) using Golden Gate Assembly (NEB). pMK232 was a gift from Masato Kanemaki (Addgene plasmid # 72834; http://n2t.net/addgene:72834; RRID:Addgene_72834).

For the UPF3 WT and chimeric protein plasmids, human UPF3A (CCDS9543.1) domains, N‐terminus (2–62), UPF2‐binding domain (63–160), Mid (163–385), and C‐terminus (386–476), are replaced by corresponding human UPF3B (CCDS14587.1) domains, N‐terminus (2–45), UPF2‐binding domain (46–143), Mid (146–370), and C‐terminus (371–470). UPF3 and chimeric proteins DNA are cloned into pcDNA3ez‐FLAG plasmid using BamHI and XbaI as previously described (Singh *et al*, 2007; Mabin *et al*, 2018). For CASC3 expression plasmid, full‐length CASC3 and CASC3‐HDAA mutant are cloned into pcDNA3ez with EcoRI and XbaI.

For PiggyBac transposase expression plasmid, hyPBase sequence (Yusa *et al*, 2011) was codon optimized for human cell expression and a synthetic DNA was cloned into mammalian expression vector pTwist‐CMV‐Beta‐Globin by Twist Bioscience. PiggyBac transposon plasmids with Tet‐ON system are made from PB‐TRE‐EGFP‐EF1a‐rtTA plasmid in Addgene #104454, which was a gift from Volker Busskamp (Addgene plasmid # 104454; http://n2t.net/addgene:104454; RRID:Addgene_104454). EGFP inserts in this plasmid were replaced by restriction sites NheI and NotI, and the puromycin resistance marker was replaced by neomycin resistance marker using Gibson Assembly (NEB). FLAG‐tagged gene of interests are moved to the PiggyBac plasmid from pcDNA3 using NheI and NotI site.

Plasmids expressing tet‐inducible and control β‐globin NMD reporters were previously described (Lykke‐Andersen *et al*, 2000; Singh *et al*, 2007).

### Cell culture

HCT116 (ATCC) and HeLa Tet‐Off (Takara) cell lines were cultured at 37°C and 5% carbon dioxide in a humidified chamber. McCoy's 5A (Modified) Medium (Gibco) for HCT116 cells and Dulbecco's Modified Eagle Medium with High Glucose (Gibco) for HeLa Tet‐Off cells were supplemented with 10% Fetal Bovine Serum (Sigma) and 1% Penicillin‐Streptomycin (Fisher).

### Cell transfection for transient or stable expression

For protein knockdown using siRNA, 1.6 μl of RNAiMAX, 60 pmol of siRNA and 200 μl OMEM was incubated for 20 min following manufacturer protocol. 3 × 10^5^ cells were then added to the transfection mixture in a 6‐well plate. Forty‐eight hours after initial transfection, total RNA was harvested. siRNA sequences are provided in Appendix Table S1.

For transient expression of proteins in HeLa cells, plasmids were transfected using JetPrime (PolyPlus) transfection reagent following manufacturer protocol with one‐fifth of recommended DNA (e.g., 200 ng DNA was used per well of a 12‐well plate if the user manual recommend 1,000 ng DNA). 24–48 h later, cells were harvested for immunoprecipitation or northern blot.

To make stable PiggyBac cell lines, 286 ng of pTwist‐CMV‐BetaGlobin‐hyPBase plasmid was co‐transfected with 714 ng of transposon plasmid (neomycin resistant) that carries the gene of interest into a 6‐well plate with 3 × 10^5^ cells seeded a day before. Forty‐eight hours post transfection, cells were trypsinized and expanded under 600 μg/ml G418 selection for 2 weeks. Polyclonal cells resistant to G418 were then expanded and frozen for further experiments. To induce the protein expression from polyclonal PiggyBac stable cells, 100 ng/ml of final doxycycline was added to the medium and cells were harvested after 24 h for immunoprecipitation or RNA extraction.

### Electroporation

For electroporating CRISPR‐Cas9 complexes into HeLa or HCT116 cells, ~2.5 × 10^5^ cells were washed in PBS and resuspended in Ingenio Electroporation Solution (Mirus) with 1–2 μM RNP complex to a final volume of 50 μl. The electroporation mix was then transferred to a Gene Pulser Electroporation Cuvette (0.2‐cm gap). Electroporation was performed with Gene Pulser Xcell Electroporation Systems (Bio‐Rad) under the following conditions: HCT 116 cells: 120 V, 13 ms/per pulse, 2 pulses with 1 s interval; HeLa cells: 130 V, 950 µF capacitance, exponential decay pulse.

### CRISPR‐Cas9‐mediated knockout and knockin

For 3B^Δ2BD^, two pX330 plasmids carrying two guide RNA sequences were co‐transfected into HCT116 cells using JetOptimus (PolyPlus) as described above. After 2–3 weeks, single clones were isolated and screened for genomic deletion.

For 3A^KO#1^, 3B^KO^ and 3B^KO#3^, two guide RNAs per gene were synthesized (IDT or Synthego) in either crRNA or sgRNA format. Guide RNA spacer sequences used are provided in the Appendix Table S2. 50 pmol of Cas9 recombinant protein (Berkeley Q3) and 60 pmol of each guide RNA (crRNA will need to be annealed to tracrRNA according to protocol provided by IDT) were incubated for ~20 min in 10 µl reaction supplemented with Ingenio Electroporation Solution. 2.5 × 10^5^ cells were then mixed with CRISPR‐Cas9 RNP complex and a final volume of 50 μl was used for the final electroporation reaction as described above. After 2–3 weeks, single clones were isolated and screened for genomic deletion.

For resistance marker‐based knockouts, donor plasmids carrying antibiotic resistance genes and homology arms along with pX330 plasmid expressing guide RNAs that targeted Cas9 close to the insert site were co‐transfected using JetOptimus (PolyPlus).

For knock‐in of small affinity tags, HCT116 cells were synchronized using 2 μg/ml aphidicolin overnight and the synchronization was released 4 h before the electroporation of CRISPR RNP complex. CRISPR RNP complex was prepared as described above and supplemented with 150 pmol of ssODN (50 nt homology arms each side of the affinity tag; sequences provided in the Appendix Table S3). Electroporation was performed using Gene Pulser Xcell Electroporation System as described above.

For MYC‐UPF2, we were unable to achieve efficient knock‐in without selection. We used a resistance marker‐based knock‐in approach where a donor plasmid carrying hygromycin resistance marker‐P2A‐MYC tag in frame with UPF2 ORF was co‐transfected with pX330 expressing guide RNA targeting a site close to the UPF2 start codon. Hygromycin resistant clones were isolated and screened for correct insert. All DNA sequences edited via CRISPR‐Cas9 were confirmed by Sanger sequencing.

### RNA extraction

Cells were homogenized in TRI‐reagent and RNA was extracted using one of three different methods. (i) RNA extraction was performed following the manufacturer’s protocol, and then the extracted RNA was treated with 2 units of DNase I (NEB) and further cleaned up via phenol‐chloroform (pH 4.3) extraction and standard ethanol precipitation. (ii) One volume of ethanol was added to the TRI‐reagent homogenized sample and the mixture was loaded onto a silica column (Epoch). The flowthrough was discarded and the column was washed once with high salt wash buffer (1.2 M Guanidine Thiocyanate; 10 mM Tris‐HCl pH 7.0; 66% Ethanol) and twice with low salt wash buffer (10 mM Tris‐HCl pH 7.0; 80% Ethanol). RNAs were eluted with water and subject to DNase I treatment as above. To the DNase I digested RNA, three volumes of RNA binding buffer (5.5 M Guanidine Thiocyanate; 0.55 M Sodium Acetate; 10 mM EDTA) and 4 volumes of ethanol were added. The mixture was then loaded into a silica column as above and washed twice with low salt wash buffer. RNA was then eluted in water. (iii) All steps followed the second method with two exceptions: The silica columns were replaced with carboxylate‐modified hydrophobic magnetic beads (Cytiva) and the DNase I digestion was performed in the presence of beads (RNA gets eluted from beads upon addition of DNase I digestion mix). RNA was then re‐bound to the magnetic beads by adding five volumes of ethanol. RNAs are then washed with low salt wash buffer twice before eluting in water.

### RT‐qPCR

A total of 1.5 µg total RNA was reverse transcribed using Maxima RNaseH Minus Reverse Transcriptase following manufacturer’s protocol except that only 0.4 µl of the reverse transcriptase was added instead of 1 µl. cDNAs were then diluted to 5 ng/µl, and 3 µl of diluted cDNA was used per reaction. qPCR reactions were set up using iTaq Universal SYBR Green Supermix (Bio‐Rad) with triplicates of 10 µl per reaction. qPCR was performed on a CFX‐connect (Bio‐Rad) equipment. Sequences of all oligos used for RT‐qPCR are provided in the Appendix Table S4. Biological replicates represent cell lines grown and harvested on different days.

### β‐globin reporter assays and Northern blots

For pulse‐chase assays, 75,000 HeLa Tet‐off cells were plated in each well of a 12‐well plate. After 24 h, reporter mRNA and protein expression plasmids were transfected using JetPrime, following manufacturer’s protocol (using 3:1 ratio of reagent to µg DNA). Cells were transfected with 200 ng pTet2 β39 plasmid, 20 ng β‐GAP internal control, 10 ng pcDNA3ez‐YFP, and 20 ng pcDNA3ez‐FLAG‐UPF3A/B. 100 ng/ml Tetracycline was used to suppress β39 expression. After 24 h, tetracycline was removed to induce β39 expression overnight (~16 h). Tetracycline (1 µg/ml) was added, and cells were harvested in 0.5 ml TRIzol at indicated time points. For steady state assays, cells were transfected and induced in the same way as the reporter decay assay. At the day of harvesting, 1 µg/ml of tetracycline was added to all cells and cells are harvested 4–6 h post transcription shut‐off. RNA was extracted as above and Northern blotting was performed as described previously (Mabin *et al*, 2018).

### Protein immunoprecipitation

Cells were washed with PBS and lysed with Gentle Hypotonic Lysis Buffer (20 mM Tris‐HCl pH 7.5; 15 mM NaCl; 10 mM EDTA; 0.1% Triton X‐100; 1× protease inhibitor cocktail; EDTA was replaced with 0.6 mM MgCl_2_ for magnesium‐dependent IP (Appendix Fig S6B)). A short 4–6 s sonication pulse (10% amplitude) was applied to solubilize the chromatin fraction. 2–5 µl of the FLAG magnetic beads (Sigma) for FLAG‐IP, and ~1 µg primary antibody conjugated with protein‐A dynabeads (Thermo Fisher) for EIF4A3‐IP or CASC3‐IP, were added to cell lysates and nutated at 4°C for 30–60 min. Magnetic beads were then washed 8 times with Isotonic Wash Buffer (20 mM Tris‐HCl pH 7.5; 150 mM NaCl; 0.1% IGEPAL CA‐630). FLAG proteins were eluted for 10–20 min with 250 μg/ml 3× FLAG peptide (APExBIO) in Isotonic Wash Buffer at 37°C. Primary antibody conjugated with protein‐A beads were eluted for 5 mins in Clear Sample Buffer (100 mM Tris‐HCl pH 6.8; 4% SDS; 10 mM EDTA) at 37°C.

### Western blotting

SDS‐PAGE was performed in Bio‐Rad Mini Trans‐Blot Cell and transferred to a nitrocellulose membrane using Trans‐Blot Turbo system. After incubating membrane with primary antibodies, infrared fluorophore conjugated secondary antibodies were used and the membrane were imaged with LI‐COR Odyssey CLx imager. Appendix Table S5 lists all the primary antibodies used.

### Total RNA‐Seq library construction

A total of 800 ng of total RNA was rRNA‐depleted using RiboCop rRNA Depletion Kit V1.3 (Lexogen) following manufacturer’s protocol. Libraries were then constructed using CORALL Total RNA‐Seq Library Prep Kit (Lexogen). Libraries were quantified on RNA TapeStation and mixed at equimolar ratio for paired‐end (2 × 150 bp) sequencing using HiSeq4000 (Novogene) platform. Due to the relatively short fragment length of our libraries, we used only read 1 sequence for downstream analysis. We had three batches of experiments performed at different times, and only RNA‐Seq samples sequenced at the same time were compared during the downstream analysis.

### Total RNA‐Seq analysis

A reference script for mapping RNA‐Seq libraries to the reference genome was kindly provided by Lexogen. For every fastq file, the first 10 bp of each read (UMI) were extracted and appended to the header line using a custom Awk script and saved to a new file together with the remainder of the reads starting at position 13. Adapter trimming was then performed with cutadapt (Martin, 2011) for the sequence “AGATCGGAAGAGCACACGTCTGAACTCCAGTCAC”. Trimmed reads were aligned to the reference genome (GRCh38.p13) using STAR aligner (Dobin *et al*, 2013). Each output bam file was then indexed with samtools (Danecek *et al*, 2021) and deduplicated with UMI‐tools (Smith *et al*, 2017) using the umi_tools dedup command with the [‐‐method=unique ‐‐multimapping‐detection‐method=NH] options. The fastq file with deduplicated reads was then extracted from the deduplicated bam file using samtools. Next, deduplicated fastq files served as input into pseudoalignment tool Kallisto (Bray *et al*, 2016) to quantify transcript abundance based on the Ensembl release 100 transcript reference. Tximport (Soneson *et al*, 2015) was used to extract transcript abundance from Kallisto results and generate count matrices for DESeq2. Transcript per million (TPM) values reported from Kallisto for each transcript were averaged for each experimental condition. We filtered out all the transcripts that have TPM less than 1 in all experimental conditions. After filtering out the transcripts, we use the RUVSeq (Risso *et al*, 2014) package [RUVs‐method] to remove unwanted variation following the instruction manual. DESeq2 (Love *et al*, 2014) was then used to identify differentially expressed transcripts and calculate their fold changes.

### PTC^+^ and PTC^−^ transcript abundance analysis

To generate a list of PTC^+^ transcripts and their PTC^−^ counterparts, we used a custom Python script that takes all human transcripts in Ensembl annotation (version 100) along with their exon and 3′UTR coordinates to annotate each transcript as PTC^+^ if the 3′UTR begins more than 50 nt upstream of the exon junction or if there are more than one exon junctions downstream of the stop codon. Thus, transcripts that acquire a reading frame‐interrupting stop codon due to altered splicing or those transcripts that contain an intron 50 nt downstream of a normal stop codon (i.e., 3’UTR introns) will be identified as PTC^+^ by this approach. PTC^−^ transcripts are those that lack these features but are expressed from genes that express at least one PTC^+^ transcript. Among expressed transcripts in our experiments (i.e., TPM > 1), there are 3,566 PTC^+^ and 5,549 PTC^−^ transcripts from 2,430 genes with a median TPM value of 1.2 and 2.5, respectively. To compare abundance of PTC^+^ and PTC^−^ groups in any two conditions, PTC^+^ and their corresponding PTC^−^ transcripts were identified in the outputs of DESeq2 analysis of RNA‐Seq data from the given conditions. The fold change values thus reported by the DESeq2 analysis for all detectable PTC^+^ transcripts and for the corresponding PTC^−^ transcripts were plotted as cumulative distribution function (CDF) in R using function *ecdf*. The distributions were compared using Kolmogorov–Smirnov (KS) test. All plotted data are available in Source Data files for main and expanded view figures.

### NMD^+^ and NMD^−^ transcript abundance analysis

To define high confidence NMD targets, we used UPF1, SMG6, and SMG7 knockdown datasets from Colombo *et al* (2017). The raw data from these datasets were quantified using Kallisto based on Ensembl release 100 transcript reference as described above. We filtered out all the transcripts that have TPM less than 1 in all experimental conditions (UPF1KD, SMG6KD, SMG7KD, wild type). The differential expression analysis was performed using DESeq2. We defined those transcripts as NMD^+^ transcripts if they have an adjusted *P*‐value of less than 0.05 and a fold change greater than 1.2 in at least two of the three knockdown conditions. If instead a transcript has a fold change less than 1.2 in either direction in at least two of the three knockdown conditions, we defined it as an NMD^−^ transcript. A minimal fold change in only two of the three knockdown conditions is a conservative choice as more stringent criteria would further reduce the chance of false negatives and thus increase the observed differences between NMD targets and controls. We further selected only those transcripts that are expressed in HCT116 cells based on our RNA‐Seq experiment (TPM > 1). From a total of 5,004 genes, this results in 3,150 NMD^+^ transcripts and 5,953 NMD^−^ transcripts with a median TPM value of 1.8 and 2.3, respectively. A total of 379 transcripts are common between NMD^+^ and PTC^+^ transcripts and 948 transcripts are shared between NMD^−^ and PTC^−^ transcripts. Transcript level comparisons between any two conditions were carried out using DESeq2, NMD^+^ and NMD^−^ transcripts were identified in the DESeq2 outputs, and the resulting fold change values for NMD^+^ and NMD^−^ transcripts were graphed as CDF plots and statistically evaluated as described above for PTC^+/−^ transcripts. All plotted data are available in Source Data files for main and expanded view figures.

### Analysis of stringent NMD targets

We defined NMD^+^ transcripts as “stringent” NMD targets if they were expressed from human genes previously reported to encode mRNAs that (i) exhibit increased half‐lives upon UPF1 knockdown and (ii) are enriched in phosphor‐UPF1 immunoprecipitates (Imamachi *et al*, 2016)). This resulted in 93 NMD^+^ transcripts from 93 genes for which fold change values from DESeq2 analysis of four key comparisons were plotted as a clustered heatmap using R package *gplots* (*heatmap.2* function).

### RIPiT‐Seq

RIPiT‐Seq was performed as described (Yi & Singh, 2021). Four biological replicates each were performed for FLAG‐MAGOH:EIF4A3, FLAG‐UPF3B:EIF4A3, and FLAG‐CASC3:EIF4A3 and sequenced on the HiSeq4000 (Novogene) platform.

### RIPiT‐Seq quantification and differential occupancy analysis

Four replicates each of MAGOH‐EJC, CASC3‐EJC, and UPF3B‐EJC RIPiT‐Seq were obtained, for a total of 12 samples. RIPiT‐Seq data analysis was performed similarly to our previous studies (Mabin *et al*, 2018; Patton *et al*, 2020). In short, the first 8 bp of each read (UMI) were extracted and appended to the header line using a custom Awk script and saved to a new file together with the remainder of the reads starting at position 9. Cutadapt (Martin, 2011) [‐‐discard‐untrimmed ‐g ^CC ‐‐no‐indels | ‐‐discard‐untrimmed ‐O 12 ‐a TGGAATTCTCGGGTGCCAAGG ‐] is used to retain any reads start with “CC” and ends with mirCat‐33 adapter. Fastq files are further cleaned up by only retaining reads unable to align to a custom reference of abundant RNA sequences using STAR aligner [‐‐outReadsUnmapped Fastx]. Trimmed reads were aligned to the reference genome (GRCh38.p13) using STAR aligner (Dobin *et al*, 2013). EJC signal for each gene was quantified using reads that overlap with the canonical EJC site (−39 to −9bp of 3′ end of non‐last exon) and was averaged over all canonical EJC sites of a transcript (i.e., intron count). Any gene with EJC counts RPKM ≦ 5 was removed. Gene‐level EJC signal was then input into DESeq2 for differential gene expression analysis (Love *et al*, 2014).

### Meta‐exon analysis

RIPiT replicates and the exon annotation were used to compute total read depth as a function of distance from the 5′ start and 3′ end of each exon. Genes with less than 10 reads were discarded. Each remaining gene’s coverage distribution was normalized by the total number of reads of that gene and such normalized distributions were averaged across all genes. The average read distribution was then plotted with respect to the distance to the start or the end of the exon.

### Expression normalized RIPiT comparisons

Reads mapping to the canonical EJC region for each RIPiT‐Seq sample were normalized by the total length of canonical region for each gene, and this length normalized EJC signal for each gene was divided by the RPKM of that gene from total RNA‐seq. The resulting expression normalized signal for the CASC3, UPF3B, and MAGOH RIPiT‐Seq were then correlated to the NMD efficiency of each gene that contains at least one PTC^+^ and one PTC^−^ isoform. NMD efficiency of each gene is marked by the highest fold change of any of the PTC^+^ isoforms in 3B^Δ2BD^:siUPF3A compared with WT:siNC.

## Author contributions

**Zhongxia Yi:** Conceptualization; Investigation; Writing—original draft; Writing ‐ review & editing. **René M Arvola:** Investigation; Writing—review & editing. **Sean Myers:** Investigation; Writing—review & editing. **Corinne N Dilsavor:** Investigation. **Rabab Abu Alhasan:** Investigation. **Bayley N Carter:** Investigation. **Robert D Patton:** Investigation; Writing—review & editing. **Ralf Bundschuh:** Supervision; Writing—review & editing. **Guramrit Singh:** Conceptualization; Supervision; Writing—review & editing.

In addition to the CRediT author contributions listed above, the contributions in detail are:

Conceptualization: ZY, GS; Investigation: ZY, RMA, SM, CND, RAA, BNC, RDP; Writing—Original Draft: ZY, GS; Writing—Review and Editing: ZY, RMA, SM, RDP, RB, GS; Supervision: RB, GS.

## Disclosures and competing interest statement

The authors declare that they have no conflict of interest.

## Supporting information



Appendix

Expanded View Figures PDF

Source Data for Expanded View and Appendix

Source Data for Figure 1

Source Data for Figure 2

Source Data for Figure 5

Source Data for Figure 6

## Data Availability

RNA‐Seq and RIPiT‐Seq data: Gene Expression Omnibus GSE179843 (https://www.ncbi.nlm.nih.gov/geo/query/acc.cgi?acc=GSE179843).
