# Supplementary material for: Mammalian UPF3A and UPF3B can activate nonsense‐mediated mRNA decay independently of their exon junction complex binding
Source: EMBO J. 2022 Apr 22;41(10):e109202. doi: 10.15252/embj.2021109202 (PMC9108626; doi:10.15252/embj.2021109202)
Supplement: Supplementary file 1 — Appendix [file EMBJ-41-e109202-s007.pdf]

Appendix Table of Contents

Appendix Figure S1: CASC3 regulates UPF3A/3B-dependent NMD	1
Appendix Table S1: siRNAs used in this study	2
Appendix Table S2: Guide RNA spacer sequences used in this study	3
Appendix Table S3: ssODNs used in this study	4
Appendix Table S4: qPCR oligos used in this study	5
Appendix Table S5: Primary antibodies used in this study	6

Appendix Figure S1

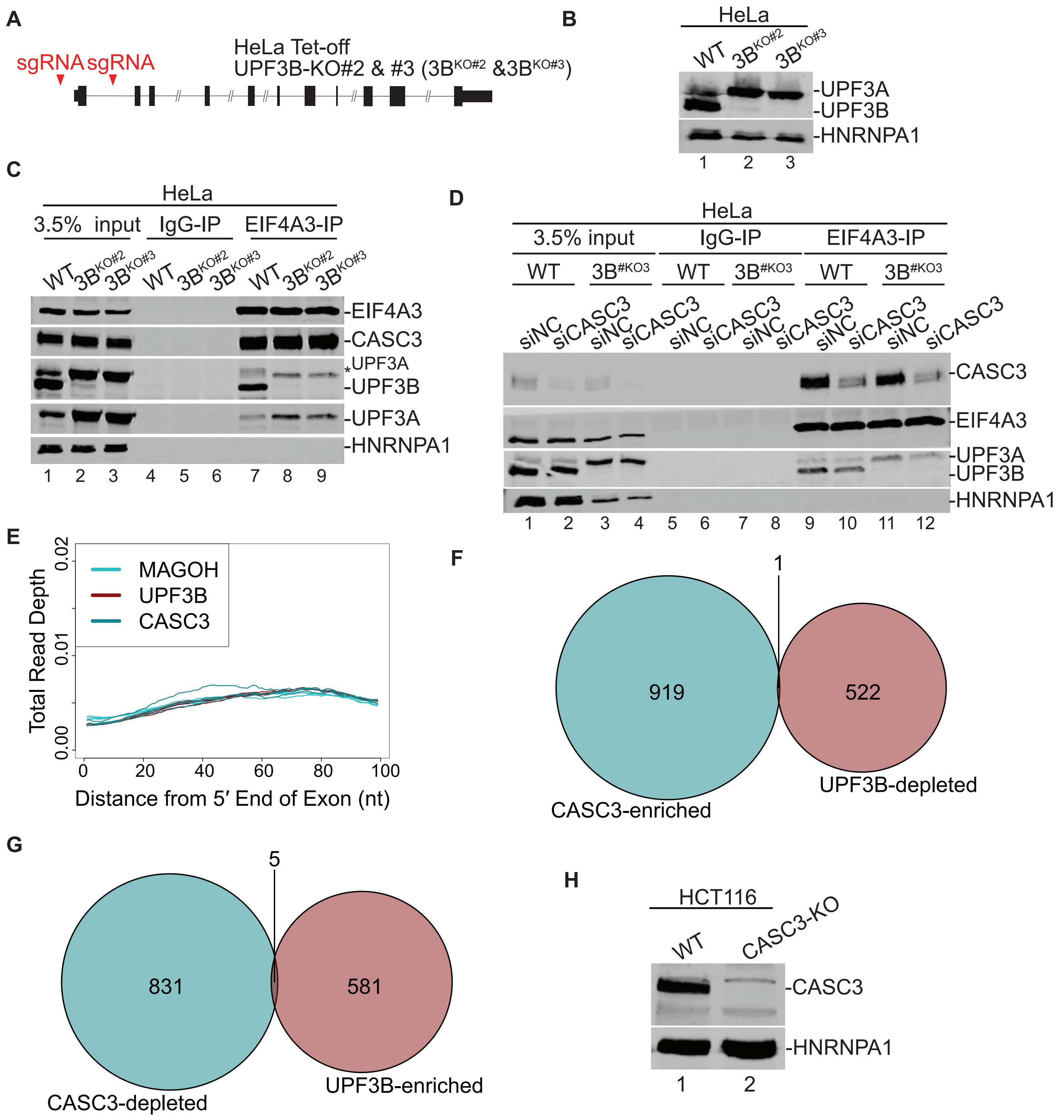

Appendix Figure S1. CASC3 regulates UPF3A/3B-dependent NMD.
A. Schematic of UPF3B knockout in HeLa Tet-off cells using CRISPR-Cas9. Red arrows represent two guide RNA targeting sites which will lead to the deletion of the first exon.
B. Protein immunoblot of UPF3B protein in WT and 3BKO HeLa cells. HNRNPA1 is a loading control.
C. Western blots showing proteins on the right in input and EIF4A3-IP from WT and 3BKO HeLa cells. Normal rabbit IgG is used for control IP.
D. Western blots as in C from HeLa WT and 3BKO cells transfected with either siNC or siCASC3.
E. Meta-exon plot of MAGOH:EIF4A3, UPF3B:EIF4A3, and CASC3:EIF4A3 RIPiT-Seq read-distribution in the 100 nt region from the exon 5′ end.
F, G. Venn diagram of significantly enriched/depleted genes in CASC3:EIF4A3 or UPF3B:EIF4A3 RIPiT-Seq samples as compared to MAGOH:EIF4A3 RIPiT-Seq.
H. Protein immunoblot of CASC3 protein in WT and CASC-KO HeLa cells. HNRNPA1 is a loading control.

Appendix Table S1

Name	Sequence	Source
siNC	NA	Qiagen (catalog# 1027281)
siUPF1	AAGAUGCAGUUCCGCUCCAUU	Horizon On-Target Plus
siUPF2	NA	Qiagen (catalog# SI04216849)
siUPF3A	UUGGGUUUCAUUCGUUUGUAA	Qiagen (catalog# SI03246411)

Appendix Table S1: siRNAs used in this study. NA = not available. All sequences are in 5' to 3' direction.

Appendix Table S2

Name	Spacer sequence	Form	Purpose
crUPF3A1_pro	auaugugcaagggcccggag	crRNA (Synthego)	UPF3A knockout
crUPF3A1_int2	gaggugaggcccgccccgag	crRNA (Synthego)	UPF3A knockout
crUPF3B1 pro	acaauauuagccgaguucag	crRNA (Synthego)	UPF3B knockout
crUPF3B1 int1	auaacuuaguaagccaacgc	crRNA (Synthego)	UPF3B knockout
crMAGOHN	cagauaaaagucacucucca	crRNA (Synthego)	FLAG-MAGOH knock-in
crUPF1N	acccggaggcaccaugagcg	crRNA (Synthego)	FLAG-UPF1 knock-in
sgCASC3N	ggccguucuccguaagaugg	sgRNA (Synthego)	FLAG-CASC3 knock-in and CASC3 knockout
sgUPF3B3	agcgggggacguagccauga	sgRNA (Synthego)	FLAG-UPF3B knock-in
sgUPF3B1	GUAACCCUGUUAACCCCCGC	pX330 plasmid	UPF3B knockout
sgUPF3B2	GAAAGUCGGGACUAUCGAUGA	pX330 plasmid	UPF3B knockout
sgUPF3B3	GAGCGGGGGACGUAGCCAUGA	pX330 plasmid	Antibiotic resistant marker knock-in for UPF3B
sgUPF3AN	GCGGAGAGUGCGGCAUGCGCU	pX330 plasmid	Antibiotic resistant marker knock-in for UPF3A
sgUPF2	GCUGGCUUUUUACGCUCAGC	pX330 plasmid	MYC-UPF2 knock-in (using antibiotic marker followed by P2A)

Appendix Table S2: Guide RNA spacer sequences used in this study. All sequences are in 5' to 3' direction.

Appendix Table S3

Name	Sequence
FLAG-UPF1	CTCGAGTGCAGCGCGGAACCGGCCCGAGGGCCCTACCCGGAGGCACCATGGATTATA AGGATGACGACGACAAAGGCGGAGGATCCAGCGTGGAGGCGTACGGGCCCAGCTCGC AGACTCTCACTTTCCTGGACAC
FLAG-MAGOH	GTGCGGCTTGCTCTTGGAAGTTCAGGCTCGGTTGTCTTTTGGGAGCCATGGATTATAAG GATGACGACGACAAAGGCGGAGGATCCGAGAGTGACTTTTATCTGCGTTACTACGTGG GGCACAAGGGCAAGTTCGG
FLAG-CASC3	GGGCGCGGTGCGTAAGTACCTCGCCGGTGGTGGCCGTTCTCCGTAAGATGGATTATAA GGATGACGACGACAAAGGCGGAGGATCCGCGGACCGGCGGCGGCAGCGCGCTTCGC AAGACACCGAGGACGAGGAATC
FLAG-UPF3B	C*C *GG CCA AGC CGC TTC AGC GGG GGA CGT AGC C AT GGA TTA CAA AGA CGA TGA TGA TAA AGG GGG CGG CGG ATC CAA GGA AGA GAA GGA GCA CAG GCC TAA GGA GAA G*C*G

Appendix Table S3: Single-stranded oligo DNA nucleotides (ssODNs) used in this study. Asterisks indicate phosphorothioate linkages. All sequences are in 5’ to 3’ direction.

Appendix Table S4

HNRNPD_NOR_F	CCAACAGGTGGTGAAGCAGT
HNRNPD_PTC_R	CTCTCGAATGCTGCCGTTTG
HNRNPD_ALL_R	AACTTCAGAGGGACCCAACG
HNRNPA2B1_NOR_F	CGATGGAG AGAGAAAAGGAA
HNRNPA2B1_NOR_R	AAGCTTTCCCCATTGTTCGT
HNRNPA2B1_PTC_F	GTTGGTAGTAAAATGGAAGGTGT
HNRNPA2B1_PTC_R	TGAAGGCACCAACAAGAACT
ILK_NOR_F	GGCTGGACAACACGGAGAA
ILK_NOR_R	CATCTCAACCACAGCAGAGC
ILK_PTC_F	ACCA GGG CTA TTG CAG TAC AAG
ILK_PTC_R	CCACTTGATCTTGGCCCCAA
NFKBIB_NOR_F	ACGAGAAATCCGGCCCCT
NFKBIB_PTC_F	TGGGTAAAGGCAGAGGGAAG
NFKBIB_NOR_R	TGAAACAAATCACACGGGGC
NFKBIB_PTC_R	GACCAATTTCTAGCCAGCCG
SRSF3_PTC_F	CAACTAGCCCTTTCAGCGTCATGTG
SRSF3_NOR_F	GTGAAAAAAGAAGTAGAAATCGTGG
SRSF3_PTC_R	TCATGTGAAACGACACCAGCCAAGC
SRSF3_NOR_R	CTCCTTCTTGGAGATCTGCGACGAG
RPS9_NOR_F	GCCATATCAG GGTCCGCAA
RPS9_PTC_F	TGGACAGGGTCCGCAAGCA
RPS9_ALL_R	CGTAGGGAGAGCGCAGAGAG
CBWD5_NOR_F	ACCGGGTATTTAG GTGCTGGGA
CBWD5_NOR_R	GCGCACTTC CTTCCCCAGAT
CBWD5_PTC_F	TGGGTTGATGCTGAATTAGGG
CBWD5_PTC_R	TGGGCCCTATCCATATGCTC
TBP_F	TGTTTCTTGGCGTGTGAAGATAACC
TBP_R	AGAAACCCTTGCGCTGGAACTCGTC
ACTB_F	CGCGAGAAGATGACCCAGAT
ACTB_R	TCACCGGAGTCCATCACGAT

Appendix Table S4: qPCR oligos used in this study. All sequences are in 5’ to 3’ direction.

Appendix Table S5

Target protein	Source	Comment
UPF1	Bethyl catalog# A301-902A	
UPF2	Bethyl catalog# A303-929A	
UPF3A	Abclonal catalog# A15893	
UPF3B	Bethyl catalog# A303-688A	
UPF3	Lykke-Andersen et al., Cell (2000)	
CASC3	Bethyl catalog# A302-471A	For IP only
CASC3	Proteintech catalog# 18047-1-AP	For WB only
EIF4A3	Bethyl catalog# A302-980A	
EIF4A3	Santa Cruz catalog# sc-365549	
MAGOH	Abcam catalog# ab180505	
RBM8A	Bethyl catalog# A301-033A	
FLAG magnetic beads	Sigma catalog# M8823-1ML	For IP only
FLAG	Proteintech catalog# 20543-1-AP	For WB only
GFP	Proteintech catalog# 66002-1-Ig	
MYC	Cell Signaling catalog# 2276S	
eRF3	Cell Signaling catalog# 14980S	

Appendix Table S5: Primary antibodies used in this study
