# Supplementary material for: Mammalian UPF3A and UPF3B can activate nonsense‐mediated mRNA decay independently of their exon junction complex binding
Source: EMBO J. 2022 Apr 22;41(10):e109202. doi: 10.15252/embj.2021109202 (PMC9108626; doi:10.15252/embj.2021109202)
Supplement: Supplementary file 2 — Expanded View Figures PDF [file EMBJ-41-e109202-s006.pdf]

# Expanded View Figures

**Figure EV1. Changes in gene expression and NMD upon *UPF3B* loss in HCT116 cells.**

A Immunoblots showing levels of proteins on the right in input or FLAG immunoprecipitates (IP) from WT and UPF3B mutant cells (indicated above each lane) expressing FLAG-tagged UPF1 protein.

B Immunoblots showing levels of proteins on the right in input or EIF4A3 IP from WT and UPF3B mutant cells as indicated above each lane.

C Alteration in expression levels of known NMD-regulated genes in the two UPF3B mutant cell lines. RT-qPCR-based quantification of expression levels of previously characterized NMD-sensitive genes (x-axis) in the two UPF3B mutant HCT116 cell lines as compared to their levels in WT cells (set to 1). Relative levels from each replicate are shown by white circles. Error bars indicate standard error of means. The asterisk (*) represents $P < 0.05$ in *t*-test with null hypothesis of true mean being 1 ($n = 3$ biological replicates).

D Immunoblot showing levels of UPF1 and UPF3B proteins in WT and $3B^{\Delta 2BD}$ cells (indicated on top) that were transfected with negative control (siNC) or UPF1-targeting (siUPF1) siRNAs. HNRNPA1 is a loading control. Knockdown percentage (KD%) of UPF1 in siUPF1-transfected cells as compared to siNC-transfected cells is shown with standard error of means (SEM).

E–G MA plots showing differential transcript expression in RNA-Seq samples from (E) $3B^{\Delta 2BD}$ cells (siNC) versus WT cells (siNC), (F) UPF1-KD (siUPF1) versus control knockdown (siNC) in $3B^{\Delta 2BD}$ cells, and (G) UPF1-KD (siUPF1) versus control knockdown (siNC) in WT cells. Each dot represents one transcript isoform with average read counts on the x axis and $\log_2$ fold change on the y axis. Transcripts that are significantly (adjusted *P*-value < 0.05) up (red)- or down (blue)- regulated > 1.5-fold and their counts are indicated.

H Cumulative Distribution Function (CDF) plots of $PTC^{+}$ isoforms and $PTC^{-}$ isoforms from same set of genes. X-axis represents fold change in UPF1-KD (siUPF1) versus control knockdown (siNC) in WT cells. Number of transcripts in each set (*n*) and *P*-value from Kolmogorov-Smirnov (KS) test comparing the two distributions are shown.

I Isoform specific RT-qPCR measuring changes in levels of $PTC^{+}$ and $PTC^{-}$ isoforms expressed from the indicated genes in WT and UPF3B mutant cells. Fold changes are with respect to the levels of $PTC^{-}$ isoforms in WT cells. Relative levels from each replicate are shown by white circles. Error bars indicate standard errors of means. The asterisk (*) represents $P < 0.05$ in *t*-test with null hypothesis of true mean being 1 ($n = 3$ biological replicates).

J Cumulative Distribution Function (CDF) plots of $NMD^{+}$ isoforms and $NMD^{-}$ isoforms. X-axis represents fold change in UPF1-KD (siUPF1) versus control knockdown (siNC) in WT cells. Number of transcripts in each set (*n*) and *P*-value from Kolmogorov-Smirnov (KS) test comparing the two distributions are shown.

Source data are available online for this figure.


© 2022 The Authors

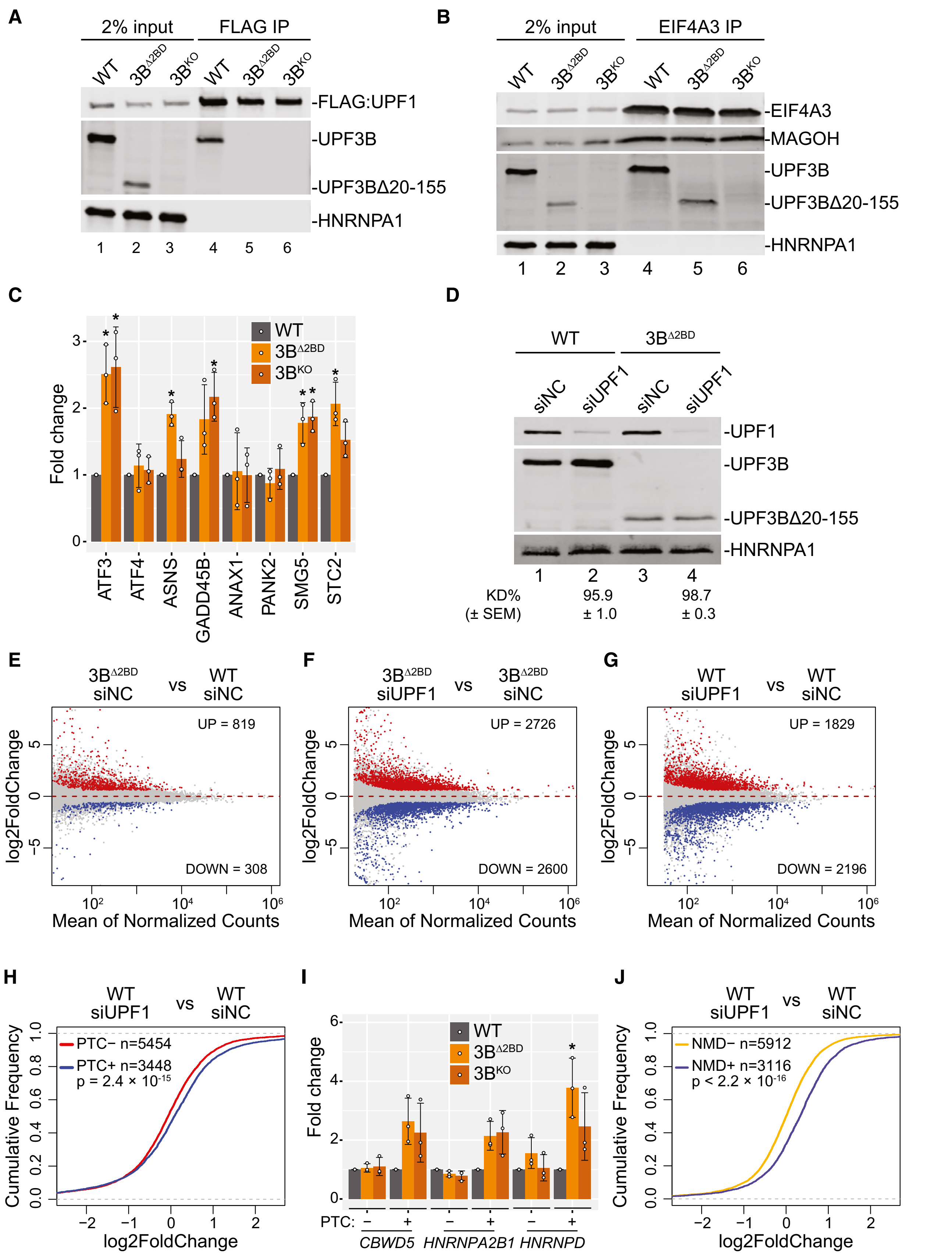
A
2% input
FLAG IP
WT
3BΔ2BD
3BKO
-FLAG:UPF1
-UPF3B
-UPF3BΔ20-155
-HNRNPA1
1 2 3 4 5 6
B
2% input
EIF4A3 IP
-EIF4A3
-MAGOH
-UPF3B
-UPF3BΔ20-155
-HNRNPA1
C
Fold change
WT
3BΔ2BD
3BKO
ATF3
ATF4
ASNS
GADD45B
ANAX1
PANK2
SMG5
STC2
D
WT
3BΔ2BD
siNC
siUPF1
-UPF1
-UPF3B
-UPF3BΔ20-155
-HNRNPA1
1 2 3 4
KD% (± SEM)
95.9 ± 1.0
98.7 ± 0.3
E
3BΔ2BD siNC vs WT siNC
UP = 819
DOWN = 308
log2FoldChange
Mean of Normalized Counts
F
3BΔ2BD siUPF1 vs 3BΔ2BD siNC
UP = 2726
DOWN = 2600
G
WT siUPF1 vs WT siNC
UP = 1829
DOWN = 2196
H
WT siUPF1 vs WT siNC
PTC− n=5454
PTC+ n=3448
p = 2.4 × 10-15
Cumulative Frequency
log2FoldChange
I
Fold change
WT
3BΔ2BD
3BKO
PTC:
CBWD5
HNRNPA2B1
HNRNPD
J
WT siUPF1 vs WT siNC
NMD− n=5912
NMD+ n=3116
p < 2.2 × 10-16
Cumulative Frequency
log2FoldChange

**Figure EV1.**

**Figure EV2. UPF3A activates NMD in the absence of UPF3B.**

A Immunoblots showing levels of proteins on the right in cells indicated above each lane. At the bottom are relative UPF3A levels after normalization to HNRNPA1 levels.

B Western blots showing levels of EJC/UPF proteins or HNRNPA1 in input, normal rabbit IgG-IP or CASC3-IP fractions from WT and UPF3B mutant cells indicated above each lane.

C Western blots showing levels of EJC/UPF proteins or HNRNPA1 in input or FLAG-MAGOH followed by MYC-UPF2 tandem-IP fractions from WT, $3A^{KO}$, and $3B^{KO}$ cells. Samples were RNase A treated during the FLAG IP. The asterisk (*) represents the mouse heavy chain of the MYC-tag antibody.

D Immunoblot showing levels of UPF3A and UPF3B proteins in WT and $3B^{\Delta 2BD}$ cells (indicated on top) that were transfected with negative control (siNC) or UPF3A-targeting (siUPF3A) siRNAs. HNRNPA1 is a loading control.

E MA plot showing differential transcript expression in RNA-Seq samples from UPF3A-KD (siUPF3A) versus control knockdown (siNC) in WT HCT116 cells. Each dot represents one transcript isoform with average read counts on the x-axis and $\log_2$ fold change on the y-axis. Red and blue dots represent > 1.5-fold up- or down-regulated transcripts, respectively, that are significantly changed (adjusted $P$-value < 0.05).

F Schematic of *UPF3A* knockout (UPF3A-KO) strategies using CRISPR-Cas9. *UPF3A* locus is in black where rectangles represent exons and horizontal line denotes introns; coding region is shown as wider rectangles. Red arrowheads represent guide RNA targeting sites. In $3A^{KO\#1}$ (top), two guide RNAs delete first and the second exons of UPF3A protein coding region. In $3A^{KO\#2}$ (bottom), a donor template is used to insert blasticidin resistant gene (BlasticidinR) and Simian Virus 40 (SV40) polyadenylation signal at the cut site.

G Immunoblot of UPF3A and UPF3B proteins in WT, $3A^{KO}$, $3B^{KO}$, and $3^{DKO}$ cells. EIF4A3 is used as a loading control. Relative expression of UPF3A (3A Rel. Exp) in $3B^{\Delta 2BD}$ and $3B^{KO}$ as compared to WT cells is shown along with standard error of means (SEM) below lanes (ND = not determined).

H Isoform specific RT-qPCR of $PTC^{+}$ and $PTC^{-}$ isoforms from the indicated genes in WT, $3A^{KO}$, $3B^{\Delta 2BD}$, $3B^{KO}$, and $3^{DKO}$ cells. Relative levels from each replicate are shown by white circles. Error bars indicate standard errors of means. The asterisk (*) represents $P < 0.05$ in $t$-test with null hypothesis of true mean being 1 ($n$ = 3 biological replicates).

 © 2022 The Authors

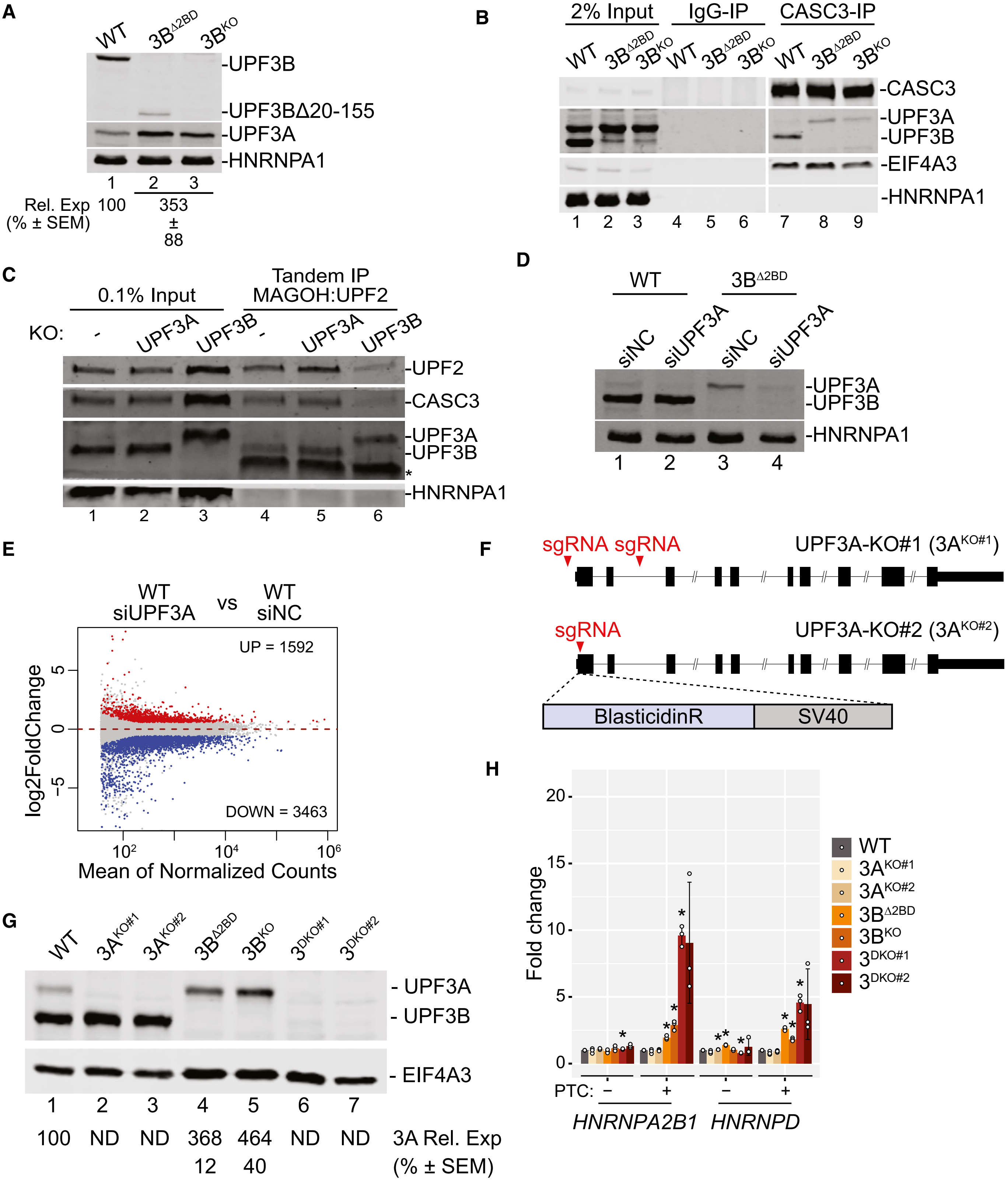
A
WT
3BΔ2BD
3BKO
-UPF3B
-UPF3BΔ20-155
-UPF3A
-HNRNPA1
1 2 3
Rel. Exp 100 353 ± 88
(% ± SEM)
B
2% Input
IgG-IP
CASC3-IP
-CASC3
-UPF3A
-UPF3B
-EIF4A3
-HNRNPA1
1 2 3 4 5 6 7 8 9
C
0.1% Input
Tandem IP MAGOH:UPF2
KO: - UPF3A UPF3B - UPF3A UPF3B
-UPF2
-CASC3
-UPF3A
-UPF3B
*
-HNRNPA1
1 2 3 4 5 6
D
WT
3BΔ2BD
siNC siUPF3A siNC siUPF3A
-UPF3A
-UPF3B
-HNRNPA1
1 2 3 4
E
WT siUPF3A vs WT siNC
UP = 1592
DOWN = 3463
log2FoldChange
Mean of Normalized Counts
F
sgRNA sgRNA
UPF3A-KO#1 (3AKO#1)
sgRNA
UPF3A-KO#2 (3AKO#2)
BlasticidinR
SV40
G
WT 3AKO#1 3AKO#2 3BΔ2BD 3BKO 3DKO#1 3DKO#2
- UPF3A
- UPF3B
- EIF4A3
1 2 3 4 5 6 7
100 ND ND 368 464 ND ND 3A Rel. Exp
12 40 (% ± SEM)
H
Fold change
PTC: – + – +
HNRNPA2B1 HNRNPD
WT
3AKO#1
3AKO#2
3BΔ2BD
3BKO
3DKO#1
3DKO#2

**Figure EV2.**

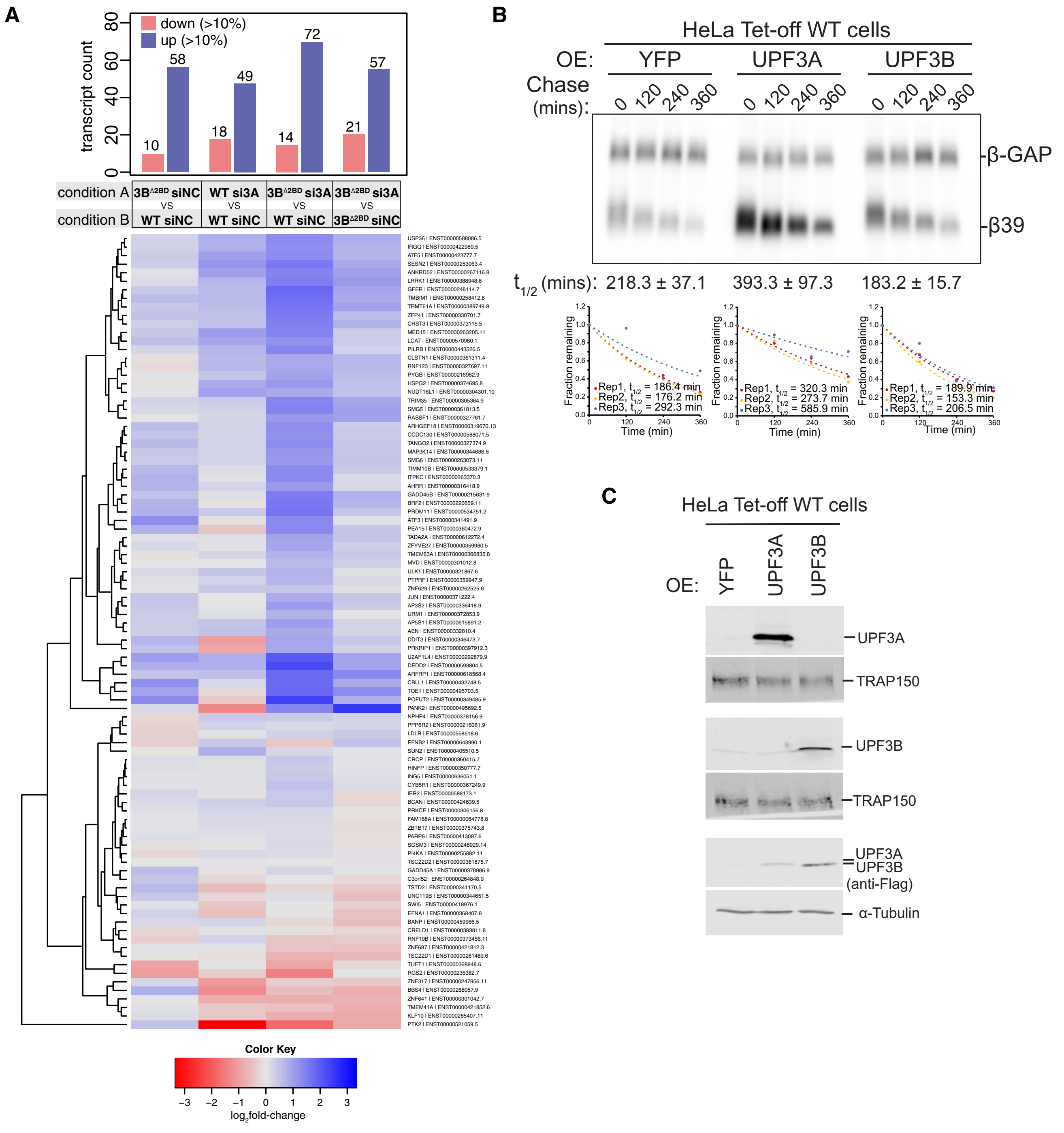


**Figure EV3. A transcript-level view of NMD activity of UPF3A.**

A Top: Bar plot showing the number of transcripts up- or down-regulated > 10% in conditions labeled on x-axis. Bottom: Heatmap of "stringent" NMD transcripts and their fold change in different comparisons. A color key for $\log_2$ fold change values is below the heatmap.

B Northern blots showing levels of β-globin reporter mRNAs in wild-type HeLa Tet-off cells. β39 is a tetracycline (Tet)-inducible reporter with a PTC at codon 39 whose levels are shown at different timepoints after transcriptional shut-off (chase) as indicated above each lane. β-GAP is a stable, constitutively-expressed, longer β-globin mRNA used as transfection control. Proteins overexpressed (OE) in each condition are indicated on top and reporter mRNA half-lives ($t_{1/2}$) along with standard errors of means are on the bottom of each blot. Below each blot are graphs where fraction of β39 mRNA remaining at various time points in three biological replicates is plotted and fitted to an exponential trendline with y-intercept set to zero; mRNA half-lives ($t_{1/2}$) for each replicate obtained from the fit are shown.

C Protein immunoblots from HeLa Tet-off cells overexpressing (OE) YFP or human UPF3 proteins as indicated above each lane. TRAP150 and α-tubulin are used as loading controls.


© 2022 The Authors

**Figure EV4. Interactions of UPF3 paralogs and their chimeras with EJC and UPF factors.**

A Protein immunoblots of input and FLAG-IP from WT and $3B^{KO\#3}$ HeLa cells transiently expressing FLAG-tagged human or mouse UPF3A as indicated above the lanes. HNRNPA1 is a loading and RNase A digestion control.

B Protein immunoblots showing expression levels of FLAG-tagged UPF3 proteins in WT and $3^{DKO\#2}$ HCT116 cells. HNRNPA1 is used as loading control. Relative fold change (Rel FC) indicates levels of UPF3B (lane 3) and UPF3A (lane 4) expression as compared to levels of their endogenous counterparts in lane 1.

C Graphs showing quantification of mRNA decay kinetics for three biological replicates of the assays shown in Fig 4A. Cell lines and overexpressed (OE) protein are indicated on the top. Fraction β39 mRNA remaining at various time points is plotted and fitted to an exponential trendline with y-intercept set to zero; mRNA half-lives ($t_{1/2}$) for each replicate obtained from the fit are shown.

D, E Protein immunoblots of FLAG-IP from $3^{DKO\#2}$ cells stably expressing different FLAG-tagged human UPF3 proteins or their chimeras. HNRNPA1 is used as loading control and RNase A digestion control. FLAG-EGFP is used as an IP control.

© 2022 The Authors

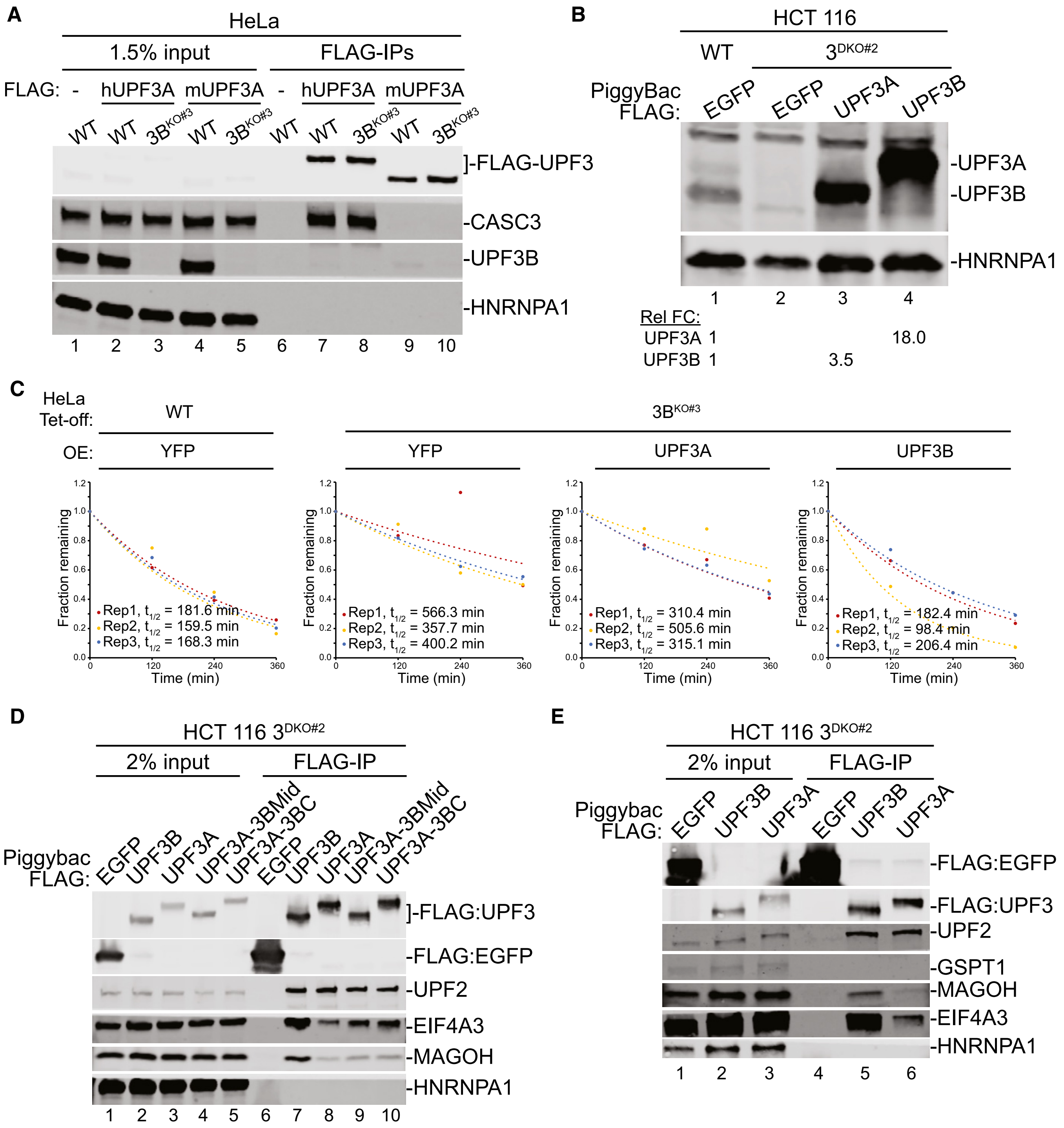


**Figure EV4.**

 © 2022 The Authors

**Figure EV5. UPF1/UPF2 sensitivity of UPF3A/3B-dependent NMD.**

A, B Immunoblots showing levels of (A) UPF1, and (B) UPF2 proteins in $3^{DKO\#2}$ cells that were transfected with negative control (siNC), (A) UPF1-targeting (siUPF1), or (B) UPF2-targeting (siUPF2) siRNAs. HNRNPA1 is a loading control.

C MA plots showing transcript-level changes upon UPF2 (siUPF2) knockdown as compared to control knockdown (siNC) in $3^{DKO\#2}$ cells. Each dot represents one transcript with average read counts on the x-axis and $\log_2$ fold change on the y-axis. Red and blue dots represent transcripts up- or down- regulated more than 1.5-fold with an adjusted $P$-value < 0.05; counts are shown on each plot.

D Cumulative Distribution Function (CDF) plots of $PTC^+$ isoforms and $PTC^-$ isoforms from same set of genes. X-axis represents fold change in UPF2-KD (siUPF2) versus control knockdown (siNC) in $3^{DKO\#2}$ cells. Number of transcripts in each set ($n$) and $P$-value from Kolmogorov-Smirnov (KS) test comparing the two distributions are shown.

E CDF plots of $NMD^+$ isoforms and $NMD^-$ isoforms. X-axis represents fold change in UPF2-KD (siUPF2) versus control knockdown (siNC) in $3^{DKO\#2}$ cells. Number of transcripts in each set ($n$) and $P$-value from Kolmogorov-Smirnov (KS) test comparing the two distributions are shown.

F MA plots showing transcript-level changes upon UPF2 (siUPF2) knockdown as compared to control knockdown (siNC) in WT cells. Each dot represents one transcript with average read counts on the x-axis and $\log_2$ fold change on the y-axis. Red and blue dots represent transcripts up- or down- regulated more than 1.5-fold with an adjusted $P$-value < 0.05; counts are shown on each plot.

G CDF plots of $PTC^+$ isoforms and $PTC^-$ isoforms from same set of genes. X-axis represents fold change in UPF2-KD (siUPF2) versus control knockdown (siNC) in WT cells. Number of transcripts in each set ($n$) and $P$-value from Kolmogorov-Smirnov (KS) test comparing the two distributions are shown.

H CDF plots of $NMD^+$ isoforms and $NMD^-$ isoforms. X-axis represents fold change in UPF2-KD (siUPF2) versus control knockdown (siNC) in WT cells. Number of transcripts in each set ($n$) and $P$-value from Kolmogorov–Smirnov (KS) test comparing the two distributions are shown.

Source data are available online for this figure.

© 2022 The Authors 

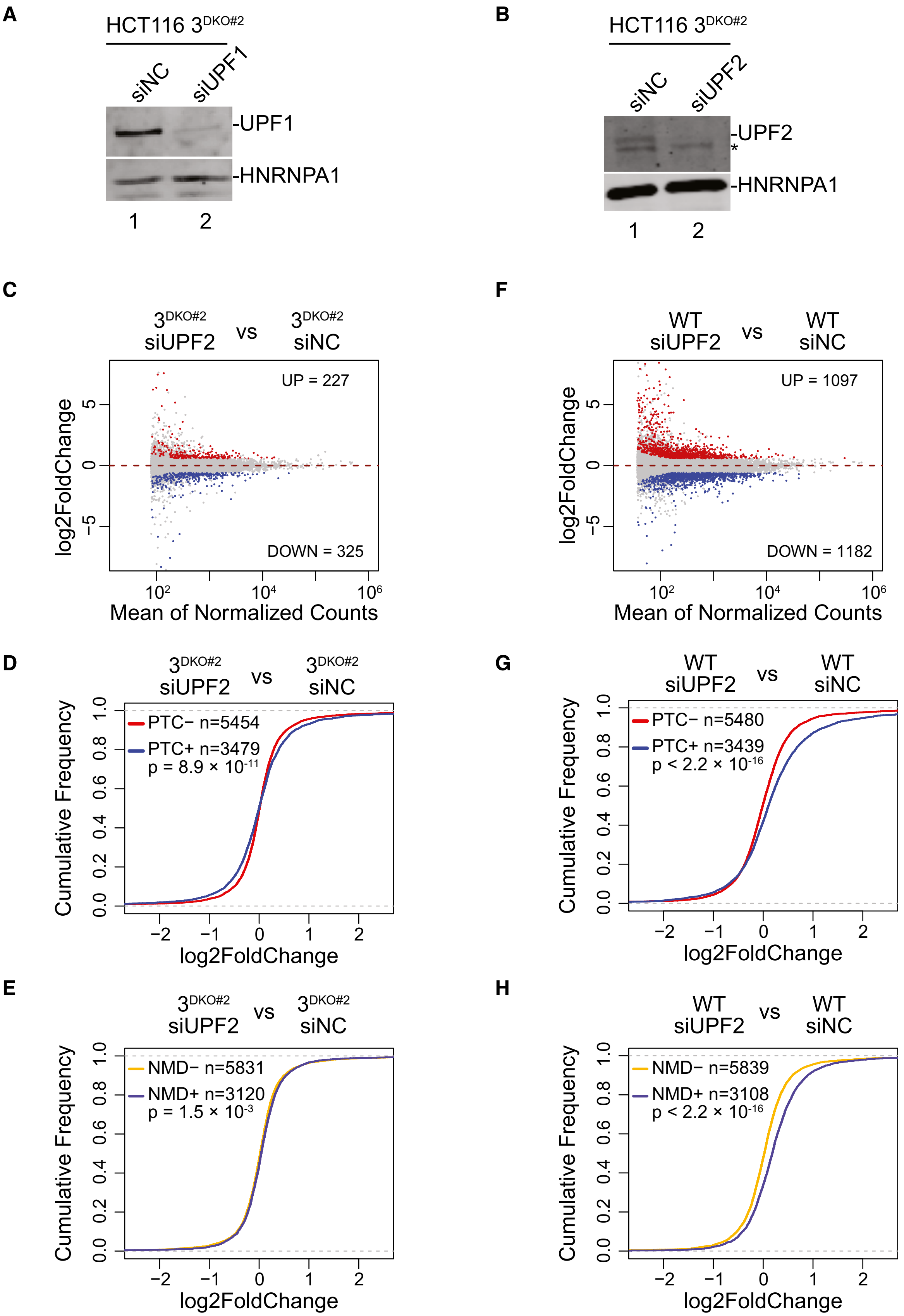


**Figure EV5.**


© 2022 The Authors
