# Supplementary figures and images for: Mammalian UPF3A and UPF3B can activate nonsense‐mediated mRNA decay independently of their exon junction complex binding
Source: EMBO J. 2022 Apr 22;41(10):e109202. doi: 10.15252/embj.2021109202 (PMC9108626; doi:10.15252/embj.2021109202)

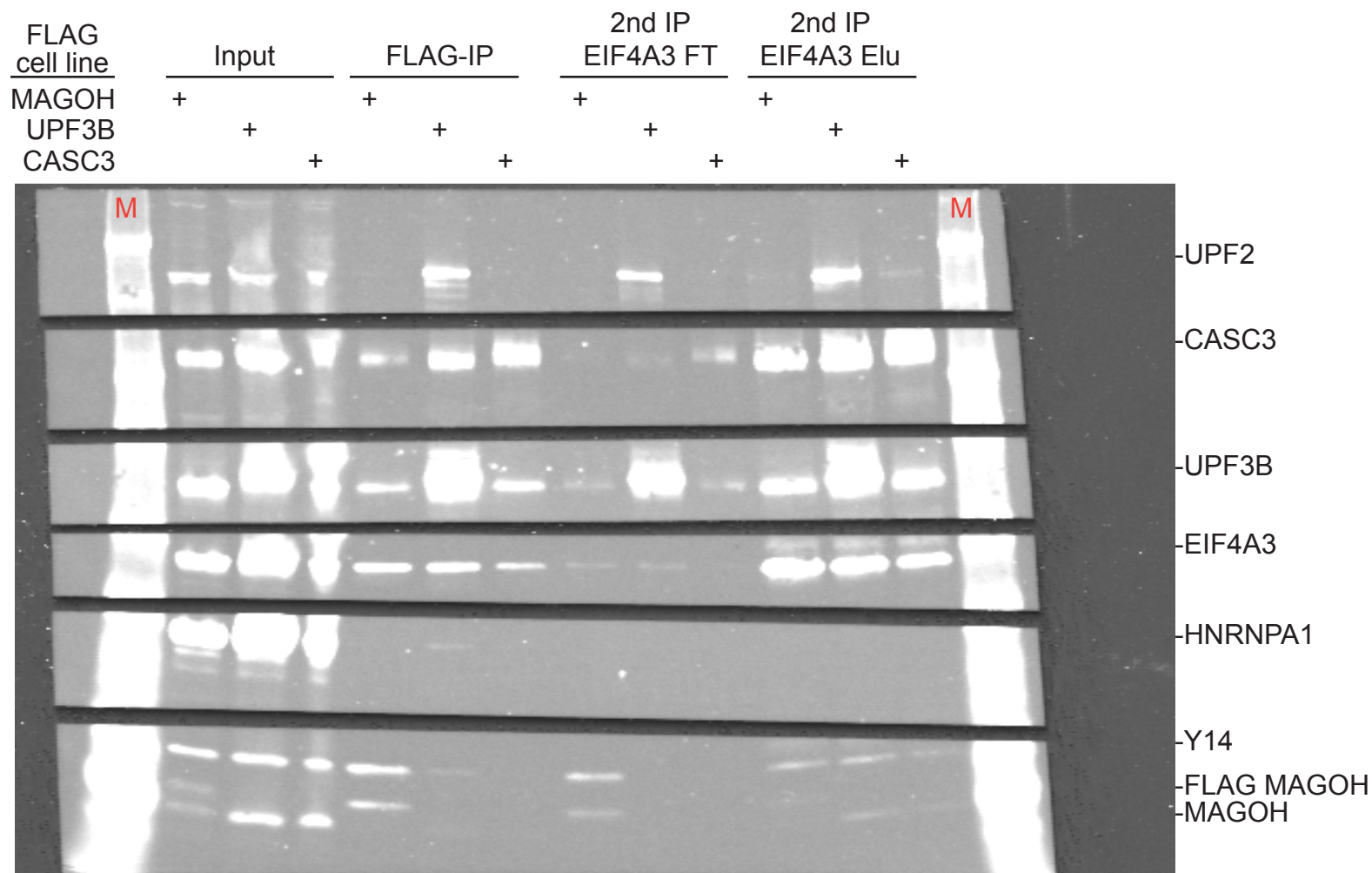

Replicate 1

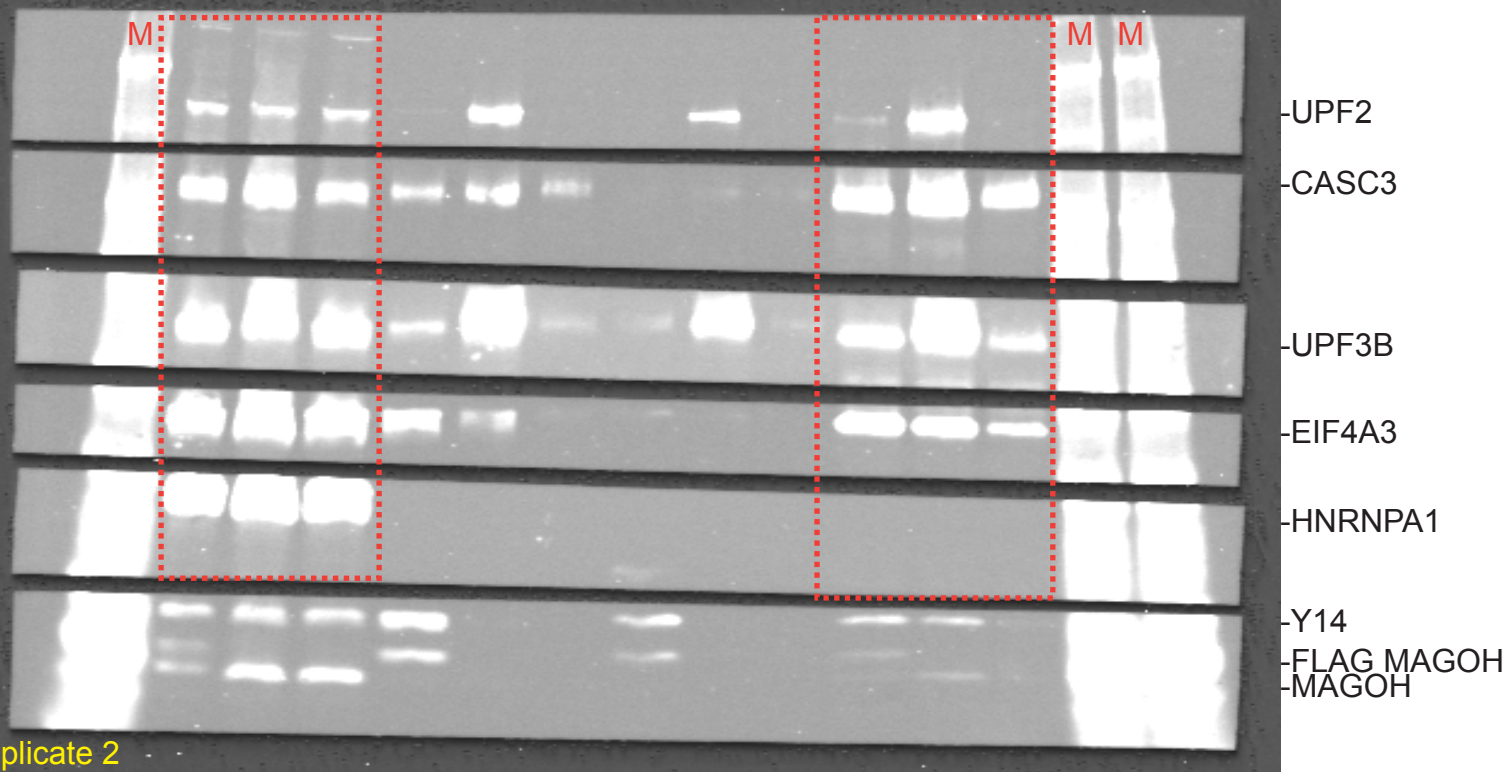

Replicate 2

Supplement: Supplementary file 7 — Source Data for Figure 6 [file EMBJ-41-e109202-s001.zip › sourceData_fig6/0003782_01_800-annotated.pdf]

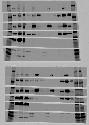

Supplement: Supplementary file 7 — Source Data for Figure 6 [file EMBJ-41-e109202-s001.zip › sourceData_fig6/0003782_01/0003782_01_TH.jpg]
