# Supplementary material for: Mammalian UPF3A and UPF3B can activate nonsense‐mediated mRNA decay independently of their exon junction complex binding
Source: EMBO J. 2022 Apr 22;41(10):e109202. doi: 10.15252/embj.2021109202 (PMC9108626; doi:10.15252/embj.2021109202)
Supplement: Supplementary file 8 [file EMBJ-41-e109202-s008.pdf]

THE EMBO JOURNAL

Mammalian UPF3A and UPF3B can activate nonsense-mediated mRNA decay independently of their exon junction complex binding

Zhongxia Yi, René Arvola, Sean Myers, Corinne Dilsavor, Rabab Abu Alhasan, Bayley Carter, Robert Patton, Ralf Bundschuh, and Guramrit Singh
DOI: 10.15252/embj.2021109202

Corresponding author(s): Guramrit Singh (singh.734@osu.edu)

Review Timeline:

Submission Date:	14th Jul 21
Editorial Decision:	27th Aug 21
Revision Received:	1st Dec 21
Editorial Decision:	18th Jan 22
Revision Received:	21st Mar 22
Accepted:	31st Mar 22

Editor: Stefanie Boehm

Transaction Report:

(Note: With the exception of the correction of typographical or spelling errors that could be a source of ambiguity, letters and reports are not edited. Depending on transfer agreements, referee reports obtained elsewhere may or may not be included in this compilation. Referee reports are anonymous unless the Referee chooses to sign their reports.)

Thank you for submitting your manuscript on the UPF3 isoforms to The EMBO Journal. We have now received three referee reports on your study, which are included below for your information. In light of these comments, we would like to invite you to prepare and submit a revised manuscript.

As you will see, the reviewers appreciate the extent of your analysis and agree on its interest for the field. However, they also raise several major concerns that must be addressed before the study can be considered further for publication. A number of these concerns can likely be resolved by textual revision and restructuring of the manuscript, as they mainly refer to presentation, organization and interpretation of the existing data (ref #2- summary, point 5, 6, 7; ref#3- summary). In addition, it will be important to address the concerns regarding the RNAseq analysis and interpretation of results (ref#2- point 2; ref#3 major concern A-F) and to further discuss the current data in the context of previous work (ref#3- major concern G, H). Furthermore, please critically review the conclusions drawn from the UPF3 domain swapping experiment and add the necessary data and/or revise the text as applicable (ref#2- point 4; ref#3- major concern I). Finally, please also carefully consider all other referee comments and revise the manuscript and figures as appropriate, as well as providing a detailed response to each comment.

Please note that it is our policy to allow only a single round of major revision. Acceptance depends on a positive outcome of a second round of review and therefore on the completeness of your responses included in the next, final version of the manuscript. We realize that lab work worldwide may currently still be affected by the COVID-19/SARS-CoV-2 pandemic and that an experimental revision can be delayed. We can extend the revision time when needed, and we have extended our 'scooping protection policy' to cover the period required for a full revision. However, it is nonetheless important to clarify any questions and concerns at this stage. Therefore, I encourage you to review the referees' comments and to contact me to discuss specific points or a preliminary revision plan in case of any uncertainties or if you have any additional questions regarding this revision.

Referee #1:

The manuscript "Mammalian UPF3A and UPF3B activate NMD independently of their EJC binding" by Yi and colleagues explores the UPF3B-dependent and independent branches of NMD. Triggering of NMD in PTC-containing nonsense mRNA was thought to be strongly dependent on the presence of the EJC, and UPF3 binding to the EJC. In their study, the authors characterize the behaviour of PTC-containing and PTC-lacking NMD targets with respect to presence or absence of the UPF3 paralogs, UPF3B and UPF3A. The main tools used in this study were generation of knockout/endogenous cell lines using CRISPR-Cas technologies and RNA-seq or variations thereof.

The major findings of the paper can be summarized as follows:

• Depletion of UPF3B affects PTC-dependent nonsense mRNA transcripts, but not all NMD-sensitive transcripts indicating that UPF3B probably mediates EJC-depdent NMD.
• The paralogue UPF3A, previously reported to be an NMD inhibitor, is upregulated in the absence of UPF3B and compensates for loss of UPF3B.
• Both UPF3 paralogs are capable of interacting with the EJC, through direct binding with CASC3 (Barentsz/MLN51) although UPF3B shows stronger interactions than UPF3A. The weaker NMD-mediating activity of UPF3A can be attributed to its weaker interaction with the EJC. Depletion/reduction of both UPF3 paralogues was shown to significantly affect NMD.
• Consistent with the 2016 study on the functions of UPF3A and UPF3B, UPF3A was shown to stabilize the beta-globin PTC39 reporter mRNA in this study. Swapping the middle domain of UPF3A with that of UPF3B appeared to alleviate the NMD-inhibitory activity of UPF3A and also enhanced the EJC-binding activity of UPF3A.
• The EJC-binding region of UPF3 appears to be dispensable for NMD as mouse UPF3A which lacks the EJC-binding motif can rescue NMD in UPF3B knockout cells.

In summary, this study presents a new role for the UPF3 proteins in NMD, one that is independent of its ability to act as a bridge between UPF1 and the EJC. The as-yet uncharacterized middle domain of UPF3 appears to play an important role in this process, which is rather intriguing in light of a recent study by Neu-Yilik and colleagues that reported the interaction of UPFB and the eukaryotic release factors as a trigger for NMD. This study challenges the established models of NMD and forces the reader to consider the diversity in triggers/molecular mechanisms of this crucial mRNA decay pathway. As such, the study presents an enormous volume of work (multiple knockout and tagged cell lines, in different backgrounds/cell strains together with high-throughput transcriptomic analysis techniques) that lead to highly significant findings and is a strong manuscript. However, certain points must be corrected/addressed before the manuscript can be accepted for publication.

1. Page 6, line 258 - the reference to the figures appears to be incorrect "(Figure 2G and Figure S2F)" should read "(Figure 2H and Figure S2G)".
2. The figure legends for Figure S2H-S2I in Supplementary Information/Expanded View Content are missing.
3. Do the authors have an explanation for the observation in Figure 3C, where although CASC3 is enriched upon Flag-UPF3B IP, UPF3B is not enriched in Flag-CASC3 IP? The amount of UPF3B co-precipitated appears to be lower than that co-precipitated by Magoh. Is it because there is a lot of free CASC3 or CASC3 in other complexes (not EJC) in cells?

Minor points:
• Poly(A) tail instead of polyA tail.
• Page 4, line 128: mention that the UPF2-binding domain of UPF3 is its RRM.
• Page 6, lines 235-237: This is an important statement. It would be great if the authors could re-word or simplify the sentence to make it readily understandable.
• Is the beta-globin reporter the only transcript where UPF3A has NMD-inhibiting activity? Have the authors identified, through their high throughput studies, any naturally occurring transcript where UPF3A acts as an NMD-inhibitor? It would be greatly beneficial if the authors could comment on this.
• It would be great if the revised manuscript were to be formatted as justified text with wider spacing between the lines, and with figures embedded within the text (instead of the very end). Makes for easier reading!

Referee #2:

Mammalian UPF3A and UPF3B activate NMD independently of their EJC binding

In this manuscript, Yi et al. evaluate the role of UPF3 paralogs, UPF3A and UPF3B, in targeting of mRNA to nonsense-mediated decay (NMD). Early studies demonstrated UPF3B as being expressed at higher levels in mammalian cells and playing the major role in eliciting NMD presumably through its binding to EJC complexes and mediating interactions with UPF2, UPF1 and the terminating ribosome; more recent reports, however, have suggested UPF3A can act as a NMD repressor and antagonize NMD as a consequence of its reduced binding to EJCs. To address these conflicting data, the authors here use CRISPR to generate knockout (KO) lines of UPF3 genes in human HCT116 cells and evaluate changes in mRNA levels by RT-qPCR or RNA-Seq. KO of UPF3B (in one case lacking full-length UPF3B but expressing a truncated variant; see below) led to increased levels of a significant number of transcripts harboring a downstream NMD-inducing EJC (compared to those without), but did not alter levels of transcripts targeted to NMD by nature of their long 3' UTR - demonstrating a specific role for UPF3B in EJC-dependent targeting of mRNA to NMD. Importantly, an additional subset of mRNAs in UPF3B KO cells were up-regulated upon siRNA-mediated depletion of the core NMD factor, UPF1, suggesting that NMD was still functional in targeting substrates in the absence of UFP3B. RNA-Seq analysis of cells depleted for UPF3A revealed no significant changes in gene expression, suggesting UPF3A acts as neither an enhancer nor repressor of NMD in these cells. In contrast, depletion of UPF3A in UPF3B KO cells led to a significant increase in transcripts harboring a downstream EJC compared to UPF3B KO alone, providing strong evidence that UPF3A compensates for loss of UPF3B and serves to activate NMD in this system. Consistent with this finding, UPF3A levels and binding to both UPF1 and EJC-proteins, EIF4A3 and CASC3, was shown to be enhanced in the absence of UPF3B. Additional RNA-Seq experiments indicated that UPF1 depletion but not UPF2 knockdown lead to up-regulation of additional EJC-dependent NMD transcripts UPF3A/UPF3B double KO cells suggesting that UPF2 functions only in the presence of UPF3 but UPF1 can target mRNA to NMD in a UPF3-independent manner.

Based on prior findings that CASC3 mediates interaction between UPF3B and the EJC, the authors showed that over-expression of CASC3 (but not a CASC3 mutant) increased association between EIF4A3 with UPF3B (in wild-type) and EIF43 and UPF3A (in UPF3B KO cells), that UPF3B-containing EJCs were highly enriched for CASC3, and that CASC3 KO led to a moderate increase in the levels of a number of EJC-dependent NMD targets as measured by RT-qPCR. Despite these data, CASC3-enriched transcripts did not significantly overlap with transcripts sensitive to UPF3B depletion, and UPF3B-EJC occupancy on an mRNA did not correlate with the efficiency of targeting the mRNA to NMD for that transcript.

Interestingly and consistent with previous reports, over-expression of UPF3A (but not UPF3B) was shown to inhibit targeting of a β-globin mRNA reporter harboring a premature termination codon to NMD, and replacement of the mid-domain from UPF3B (but not its UPF2 binding domain) into UPF3A abrogated this antagonistic function, possibly through a purported interaction with translation termination factor eRF3 (that was not able to be experimentally confirmed in vivo in this study). In contrast to the plethora of data demonstrating UPF3 binding and association with EJCs, expression of murine UPF3A in UPF3A/UPF3B double KO cells was shown to rescue NMD activity, despite mUPF3A falling to interact with human EJC components by coIP, providing intriguing evidence that UPF3A function in NMD is not mediated through its association with EJCs.

This manuscript would benefit from better presentation and organization of the data. Despite this, the authors provide sufficient evidence that UPF3A acts as a general activator in NMD in HCT116 cells and can complement loss of UPF3B in triggering EJC-dependent NMD substrates to this pathway (except in the case of the PTC-containing β-globin for which over-expression of UPF3A inhibits NMD). While a number of figures are dedicated to demonstrating UPF3 interaction with EJC components (likely mediated by CASC3), this interaction does not appear to correlate with NMD targeting efficiency. Moreover, mouse UPF3A, which fails to exhibit detectable interaction with EJC proteins in HCT116, fully complements cells lacking UPF3A/B, further suggesting that this interaction is dispensable for NMD. How UPF3 functions to mediate EJC-dependent NMD independent of binding the EJC is highly speculated in a confusing model (Figure 7) but not experimentally tested or resolved in this present work.

Specific concerns:

The UPF3B knockout cell line #1 expresses a truncated UPF3B polypeptide (lacking amino acids 20-155) at ~10% of full-length UPF3B levels (Fig. 1B, lane 2) that is predicted to maintain its EJC binding domain. This clone should not be considered a UPF3B 'knockout' as the expressed peptide could have ability to interact with EJCs and impact NMD (perhaps in a dominant-negative fashion) or mRNA metabolism generally. This possibility should be discussed and protein interaction blots presented in Fig. 2A and 2C should be expanded to display both full length and truncated UPF3B polypeptides. Given that the author isolated a second UPF3B knockout clone (i.e. #2) that appears to completely disrupt UPF3B expression, it is unclear why this clone was not used for analyzing the role of UPF3B in gene expression throughout.

The authors designate transcripts harboring a EJC >50 nt downstream of the stop codon as "PTC+" (and those lacking as "PTC-"). A more thorough explanation of how these classes of transcripts are defined (how many genes, their expression levels, etc) is needed, especially in clarifying whether they contain a premature termination codon, or, rather, just a downstream EJC.

The authors demonstrate in Fig. 2B a ~3-fold upregulation of UFP3A protein levels in UPF3B knockout cells, which is consistent with prior published findings. This finding is not recapitulated in input samples in Fig. 2A (lanes 1-4); the authors should address this inconsistency.

Domain swapping experiments presented in Fig. 5 provide evidence that the mid-domain of UPF3A is required for its ability to inhibit targeting of a PTC-containing β-globin reporter to NMD. Based on the data presented, it is inaccurate to conclude that this domain in UPF3B plays an important role in NMD activation; moreover, speculation regarding the function of this domain from UPF3B (i.e. eRF3 binding) is not supported by experiments performed by this group (Fig. S5B). If the authors want to demonstrate an importance for this domain in mediating NMD by UPF3B, then mutational analysis (or replacement with the mid-domain from 3A) needs to be performed.

The manuscript is sufficiently well written but difficult to follow, and the presentation and organization of the data is confusing. Moreover, the lack of a clear and quantitative discussion of the data (i.e. fold-changes listed in the co-IPs and cumulative frequency graphs) leaves the onus to the reader to interpret the significance of each observation and the true findings from this study. In addition, a signifiant amount of the manuscript is devoted to demonstrating UPF3A/B interact with EJC protein components, despite the fact that a key finding is that UPF3 can promote EJC-dependent NMD independent of its association with EJC.

The title indicates that neither UPF3A nor UPF3B need to bind EJCs to promote NMD in mammals. While this is directly supported by heterologous data using murine UPF3A, it is unclear what evidence directly supports this conclusion for UPF3B.

The model presented in Fig. 7 is confusing, highly speculative and presents novel functions for UPF3 not studied or supported by this work, and is better suited for a review.

Referee #3:

Yi et al. provide an intriguing study on 2 key factors in the NMD RNA turnover pathway: UPF3A and UPF3B (which I will refer to as 3A and 3B, respectively). There is widespread interest in 3A and 3B because of their association with human disease (e.g., 3B mutations cause human intellectual disability) and the evidence implicating these two factor as key regulators/factors of the NMD pathway.

Using HCT116 colorectal tumor cells for most of their studies, the authors conduct a wide range of impressive experiments to address questions about 3A/3B. They provide evidence for the following main points: First, 3B has a role in NMD, but is not absolutely necessary for NMD (verifying past studies that drew the same conclusion). Second, 3A is largely dispensable for NMD in control cells, but becomes an important back-up when 3B is absent (the first point is not well supported [see below] and the second point verifies the results of past studies). Third, the authors claim they obtained evidence from 3A/3B double knockout (ko) cells that 3A/3B act together in a unique "branch" of NMD, thereby verifying previous work using 3A/3B knockdown (kd) cells. Actually, I think the authors' data indicates instead that 3A/3B do NOT act in a branch-specific way, but rather act together to stimulate the WHOLE NMD pathway (see below). This is not a criticism; indeed, I regard this finding as very important for this field. Fourth, the authors find that 3B does not activate NMD by the means that has been assumed for more than 2 decades. In particular, previous evidence had strongly suggested that 3B is a key tethering molecule that bridges the NMD factor, UPF2, with the exon-junction complex (EJC), the latter of which is a "NMD amplifier" complex bound near exon-exon junctions after RNA splicing. The authors' evidence puts this model in grave doubt - a significant advance for the field. Fifth, the authors find that 3A can act as both an NMD activator and repressor (depending on the context), thus verifying past work (however, their evidence suggests that 3A is mainly a NMD activator in the cells they examined, which differs from a previous study showing that 3A is mainly a NMD repressor in several other cell types). Sixth, the authors identify an intriguing domain in 3A responsible for its repressor activity. Seventh, they investigate the role of the cytoplasmic EJC protein, CASC3, in 3B-dependent NMD, and make several findings, including that CASC3 promotes the association of 3B with the EJC, and that 3B and CASC3 are important for degrading a largely overlapping set of transcripts. Finally, the authors provide evidence that 3A/3B/EJC degrades largely the same transcripts as UPF2. This is important, as it contradicts previous evidence suggesting that UPF2 and the EJC act in different NMD branches, as detailed below. Together with the authors' evidence that 3A/3B does not

drive an independent branch of NMD, this MS suggests a re-evaluation of the whole concept that NMD is a branched pathway.

The breadth of the authors' experiments is both an advantage and disadvantage. A key advantage is the authors address many fundamental questions in the field. A disadvantage is the authors sometimes provide insufficient evidence for a given point to make a compelling case. Fortunately, much of this can be rectified with further analysis of the authors' existing data, as detailed below. Another disadvantage of the breadth of studies in this MS is it is often challenging to follow the train of thought through the entire MS. Indeed, this MS does not present a single story. This problem could potentially be rectified by re-organizing the MS and/or moving some sections to a follow-up MS.

Major concerns:
(A) Limitations of the authors' approach to determine whether NMD is impacted by a given factor. To determine the function of 3A, 3B, and other NMD factors, the authors generated RNAseq datasets from cells deficient (by ko or kd) in the factor of interest. Premature termination codon (PTC)+ and PTC- transcripts were defined from annotated transcripts in the Ensembl database. In accordance the well-established "-50 boundary rule" for EJC-dependent NMD, a PTC+ transcript was defined a mRNA that harbors an in-frame stop codon at least 50 nt from the last exon-exon junction. The PTC+ vs. PTC- data was represented as cumulative distribution function (CDF or MA) plots (e.g., Fig. 1C). While this statistical CDF analysis of PTC+ vs. PTC- transcripts led the authors to draw many interesting conclusions throughout the MS, there are caveats with this analysis, including that not all "PTC+ transcripts" defined by this analysis are necessarily direct NMD targets; e.g., NMD silencer elements have been defined that allow NMD escape, and alternative processing events can obscure interpretation. Conversely, many of the transcripts defined as "PTC-" ARE NMD targets; e.g., many mRNAs with long 3'UTRs are degraded by NMD. Such caveats should be mentioned by the authors. More importantly, the authors often disregard signals other than the EJC that trigger NMD, leading them to sometimes make inappropriate sweeping conclusions about the effect of specific factors on NMD. Examples are given below.
(B) Fig. 1E. Have the 4 RNAs examined by qPCR previously been shown to be EJC-dependent NMD targets, such as by EJC factor kd? Just because a transcript conforms to the -50 boundary rule does mean it is a EJC-dependent NMD target. Also, why were these particular transcripts chosen for qPCR verification? Were they chosen in an unbiased manner?
(C) Long 3'UTR-dependent NMD. The authors claim that 3B does not (or hardly) degrades mRNAs harboring long 3'UTR (a well-established NMD-inducing signal). While this conclusion may be correct, it would be more fair to the reader if the authors also briefly explained (in the Results or Discussion) the caveats of this conclusion. For example, the analysis was done on ALL transcripts. If focused on 3B and/or 3A/3B-dependent transcripts, a different conclusion might be drawn. Also, the results only pertain to HCT116 colorectal tumor cells. 3B might broadly promote the decay of mRNAs harboring long 3'UTRs in other cell types, including normal cells from other cell lineages.
(D) 3A is inconsequential as a NMD activator or repressor in HCT116 cells. This claim made by the authors is not sufficiently supported, as they only did the total PTC+ vs. PTC- transcript analysis described in A, above, to draw this conclusion. If 3A acts on a specific subset of NMD targets, as shown previously by Shum et al. 2016 in other cell types, this will likely not be revealed by such broad analysis. The authors' data clearly shows that 3A broadly regulates gene expression: Fig. S2D shows that more than 5000 transcripts are significantly dysregulated by 3A kd (with more downregulated than upregulated, consistent with 3A acting as NMD repressor). There are many alternative approaches to better assess the function of 3A in HCT116 cells, including analyzing the effect of 3A on high-confidence NMD targets identified by many past publications. Regardless of the approaches chosen by the authors, it is critical that they rigorously test the possibility that 3A regulates a subset of NMD targets, with one subset being positively regulated by 3A and another subset being negatively regulated by 3A.
(E) 3A and 3B are functionally redundant. The authors make a good case that this is correct, based on analysis of 3A kd in 3B-ko cells, as well as 3A-ko/3B-ko (dKO) cells. Furthermore, previous studies have also suggested this is the case. However, the authors analysis would be more complete if they examined not only EJC-dependent targets (by the approach described in A, above) but also analyzed previously identified high-confidence NMD targets, as in D, above. Also, their present analysis does not exclude that 3A ALSO acts as a NMD repressor (for some transcripts) in the context of 3Bko (as shown by Shum et al. in non-malignant cells). This should be dissected. Thus, while the authors analysis of 4 NMD targets by qPCR in Fig. 2H nicely demonstrates that 3A and 3B act redundantly, this does not address whether 3A can ALSO act as a repressor. Thus, they should also seek to verify the downregulation of specific transcripts in response to 3A ko or kd in the context of 3B ko.
(F) Fig. 3G. The authors interpretation of this data is 3B/EJC occupancy does not affect NMD efficiency, but the analysis includes significantly regulated transcripts that are barely misregulated (Log2FC close to 0). Thus, the authors should consider introducing reasonable fold cut-offs. Also, they could focus their analysis only on putative 3A/3B target mRNAs.
(G) Does 3A/3B promotes the decay the same EJC-dependent transcripts as UPF2? The authors claim the answer is "yes" based on reasonable evidence, but nonetheless this seems an overly strong conclusion given that the overlap between the two is not complete. Also, this appears to contradict the results of Gehring et al. Mol Cell '05, a study that obtained evidence that distinct transcripts are targeted by UPF2 vs. EJC factors, leading to the proposal that there are independent UPF2- and EJC-dependent branches. This early work only examined a handful of transcripts, so an apparent discrepancy with the current study is not surprising, but this topic is important conceptually for the field, so it should be discussed.
(H) Is there a 3A/3B-independent branch of NMD? Previous 3A/3B kd (in cell lines) and 3B ko (in mice) studies had suggested that there is a 3A/3B-independent branch of NMD. The idea was that this NMD branch degrades transcripts using the central NMD factor, UPF1, but not 3A and 3B. By completely knocking out both 3A and 3B in cells, the authors are in a unique position to rigorously test this model. Interestingly, the authors found that UPF1 kd hardly had any effect on EJC-dependent 3A/3B-independent target mRNAs. In my mind, this and all the other data from the authors suggests that 3A/3B mainly acts as an amplifier of ALL EJC-dependent NMD transcripts, NOT as an amplifier of a specific subset of NMD targets. Thus, I interpret their

data as suggesting that 3A/3B does NOT direct a specific branch of NMD. That said, this conclusion is based only on analysis of EJC-dependent transcripts (using the approach described in A, above). The authors should expand their analysis to other transcripts to more fully address whether a 3A/3B-independent NMD branch exists or not.
(I) Domain-swapping experiments. The authors interesting conclusions from these experiments are based on rescue with plasmids encoding wt 3A, wt 3B, and a panel of 3A/3B swap mutants (Fig. 5C/D). The results are likely to critically depend on expression level of the force expressed plasmids. For physiological relevance, optimally, these should be expressed at endogenous levels. Are they? If not, how much under- or over-expressed?
(J) Does 3A act independently of its EJC-binding domain? The authors' domain swapping experiments, as well as experiments with mouse 3A (which has a very weak EJC-interaction domain), suggest that 3A supports NMD without using its EJC-interaction domain. This is an extremely important concept for the field that would optimally be further tested by evaluating a 3A mutant lacking the EJC-interaction domain. However, an alternative is to refer to the Wallmeroth et al. paper, as they conducted such an experiment.

Minor concerns:

(1) Line 20. The word after "mediated" is "NMD," so the sentence is essentially saying "EJC-mediated nonsense-mediated RNA decay." Replace "mediated" with "dependent" or some similar word.
(2) The authors sometimes use the broad term "UPF3" instead of referring specifically to 3A, 3B paralog, or both. This is ok sometimes (such as on line 46), but not on lines 23, 24, 72, 73, 74, and 76. One line 261, the authors explain they use the general term "UPF3" from then on, but this will be confusing for readers that miss this line. Best to say "both UPF3 paralogs" or "UPF3A/UPF3" or something like that.
(3) UPF3 paralogs is defined twice: on lines 61 and 82.
(4) Line 99. Gehring et al. does not provide evidence for UPF3B-independent NMD.
(5) Line 131. Sentence needs revising.
(6) Line 134. Polyadenylation signals do not terminate transcription. Transcription can often proceed to many scattered sites many kb downstream of the polyadenylation signal.
(7) The Western blot method seems to be missing from the M&Ms. Also, is there a table of Abs used?
(8) Line 256. Fig. 2H, not G, right? Fig. S2F, not S2G.
(9) Line 266. Fig. S2I, not S2H, right?
(10) It is suggested to use the term "control" rather than "wild type" when referring to non-mutant or non-kd cells. WT means "non mutant," and the cell lines used by the authors are tumor cells that presumably have many mutations.
(11) Line 424. "relevant," not "present."
(12) Figs. 1E, 2H, 6C, S1A, S1F, S2G. Not clear what bars are PTC- and PTC+. To rectify, add lines above the - and + symbols that extend to the appropriate bars (e.g., 3 bars for Fig. 1E). This line should be half the length of the line above the gene names. Of course, other solutions are possible.
(13) Fig. 6C. Would be more clears if legend indicated that * means values statistically significantly different from EGFP-transfected control cells, which has a value of 1.
(14) The Discussion is generally well written. However, some suggested alterations follow. First, the authors' contributions to the key questions they list are understated in the sentence on lines 455-457. Change to something like: "By generating UPF3A- and UPF3B-deficient human cell lines, we address these important questions using a battery of different approaches." Another issue is the authors do not appear to acknowledge that the reason some of the results they obtained differ from previous studies may be because different cells were examined. For example, most of the results obtained by Shum et al. 2016 were from P19 cells, a non-malignant pluripotent cell line, as well as purified cell types from mice in vivo. This is critical, as there is evidence that NMD acts in a cell type-specific manner. The authors should also acknowledge that Shum et al. did not claim that 3A was only a NMD repressor; they also obtained evidence that 3A is also a NMD activator for some transcripts.

Response to reviewer's comments:

We would like to thank all three reviewers for their careful evaluation of our work and their constructive feedback, which has further improved our manuscript. Below we provide our point-by-point response to every concern raised by the reviewers.

Referee #1:
The manuscript "Mammalian UPF3A and UPF3B activate NMD independently of their EJC binding" by Yi and colleagues explores the UPF3B-dependent and independent branches of NMD. Triggering of NMD in PTC-containing nonsense mRNA was thought to be strongly dependent on the presence of the EJC, and UPF3 binding to the EJC. In their study, the authors characterize the behaviour of PTC-containing and PTC-lacking NMD targets with respect to presence or absence of the UPF3 paralogs, UPF3B and UPF3A. The main tools used in this study were generation of knockout/endogenous cell lines using CRISPR-Cas technologies and RNA-seq or variations thereof.

The major findings of the paper can be summarized as follows:
• Depletion of UPF3B affects PTC-dependent nonsense mRNA transcripts, but not all NMD-sensitive transcripts indicating that UPF3B probably mediates EJC-depdent NMD.
• The paralogue UPF3A, previously reported to be an NMD inhibitor, is upregulated in the absence of UPF3B and compensates for loss of UPF3B.
• Both UPF3 paralogs are capable of interacting with the EJC, through direct binding with CASC3 (Barentsz/MLN51) although UPF3B shows stronger interactions than UPF3A. The weaker NMD-mediating activity of UPF3A can be attributed to its weaker interaction with the EJC. Depletion/reduction of both UPF3 paralogues was shown to significantly affect NMD.
• Consistent with the 2016 study on the functions of UPF3A and UPF3B, UPF3A was shown to stabilize the beta-globin PTC39 reporter mRNA in this study. Swapping the middle domain of UPF3A with that of UPF3B appeared to alleviate the NMD-inhibitory activity of UPF3A and also enhanced the EJC-binding activity of UPF3A.
• The EJC-binding region of UPF3 appears to be dispensable for NMD as mouse UPF3A which lacks the EJC-binding motif can rescue NMD in UPF3B knockout cells.

In summary, this study presents a new role for the UPF3 proteins in NMD, one that is independent of its ability to act as a bridge between UPF1 and the EJC. The as-yet uncharacterized middle domain of UPF3 appears to play an important role in this process, which is rather intriguing in light of a recent study by Neu-Yilik and colleagues that reported the interaction of UPFB and the eukaryotic release factors as a trigger for NMD. This study challenges the established models of NMD and forces the reader to consider the diversity in triggers/molecular mechanisms of this crucial mRNA decay pathway. As such, the study presents an enormous volume of work (multiple knockout and tagged cell lines, in different backgrounds/cell strains together with high-throughput transcriptomic analysis techniques) that lead to highly significant findings and is a strong manuscript. However, certain points must be corrected/addressed before the manuscript can be accepted for publication.

1. Page 6, line 258 - the reference to the figures appears to be incorrect "(Figure 2G and Figure S2F)" should read "(Figure 2H and Figure S2G)".
Response: We have updated the results section including references to all figures in the text.

2. The figure legends for Figure S2H-S2I in Supplementary Information/Expanded View Content are missing.

Response: We have now carefully checked to ensure that there are no such omissions in the revised manuscript.

3. Do the authors have an explanation for the observation in Figure 3C, where although CASC3 is enriched upon Flag-UPF3B IP, UPF3B is not enriched in Flag-CASC3 IP? The amount of UPF3B co-precipitated appears to be lower than that co-precipitated by Magoh. Is it because there is a lot of free CASC3 or CASC3 in other complexes (not EJC) in cells?
Response: We agree with the reviewer that there appear to be differences in CASC3 and UPF3B co-IP efficiencies in Fig 6C (earlier 3C). However, when we normalize co-IP levels to EIF4A3, which should be indicative of EJC core levels in these complexes, UPF3B and CASC3 co-IP efficiencies are comparable (please see quantifications under lanes 4-6 of new Fig 6C).

Minor points:
• Poly(A) tail instead of polyA tail.
Response: We have made this change throughout the manuscript.

• Page 4, line 128: mention that the UPF2-binding domain of UPF3 is its RRM.
Response: We have made this addition.

• Page 6, lines 235-237: This is an important statement. It would be great if the authors could re-word or simplify the sentence to make it readily understandable.
Response: We have split this sentence into two and rephrased them.

• Is the beta-globin reporter the only transcript where UPF3A has NMD-inhibiting activity? Have the authors identified, through their high throughput studies, any naturally occurring transcript where UPF3A acts as an NMD-inhibitor? It would be greatly beneficial if the authors could comment on this.
Response: Prompted by this and similar comments from reviewer 3 below, we have expanded our analysis of endogenous NMD targets in the RNA-Seq experiments to look for genes or transcripts where UPF3A may act as NMD repressor. Specifically, we closely examined a set of ~100 genes that are defined as robust NMD targets based on UPF1 depletion leading to both gene upregulation and increase in mRNA half-life (compiled by and available as Supplemental Table S1 in Kurosaki et al, Genes & Dev 2014). Notably, this list includes several NMD factor genes that are known to be auto-regulated by NMD. In this set, while a larger subset of the genes are upregulated upon UPF3A knockdown (Figure EV3A), we do find some genes that are downregulated upon UPF3A knockdown and thus could be potential targets of UPF3A repressor activity. However, a majority of these genes become upregulated when UPF3A knockdown is combined with UPF3B loss of function. Only a few genes are consistently downregulated in all UPF3A knockdown conditions (toward the bottom of the heatmap in Figure EV3A; e.g. ASNS) and hence may be repressed by UPF3A. Importantly, however, in global analyses when effects are limited to a small group of transcripts/genes and are not amenable to statistical evaluation for being robust trends, it is not possible to dissect the direct effects on NMD from the possible indirect effects (e.g. transcriptional changes). This contrasts with clear and statistically significant upregulation we observe for PTC+ and NMD+ transcript groups as compared to their control PTC- and NMD- counterparts in many other conditions (e.g., UPF3B or UPF3A+3B depletion). Also, as PTC+ and PTC- transcripts share transcriptional controls, PTC- group serves as a good control for indirect effects of protein factor depletion. For such reasons, we avoid cherry-picking certain genes showing a trend that is not widespread and significant. Hence, in the revised manuscript, we do acknowledge the caveat that some genes can still be repressed by UPF3A but we do not make any strong conclusions about UPF3A's ability to repress endogenous transcripts in cell lines we have examined except for the beta-globin reporter.

• It would be great if the revised manuscript were to be formatted as justified text with wider spacing between the lines, and with figures embedded within the text (instead of the very end). Makes for easier reading!

__Response:__ We have increased line spacing and justified the text. We attempted inserting figures within the text but reverted to putting them at the end. Many of our figures have multiple panels, so it is difficult to fit figures and their legends on a single page and still have them be legible. In our opinion, inserting multiple pages within the main text also interrupts the flow. We hope this does not cause too much inconvenience to reviewers.

Referee #2:

Mammalian UPF3A and UPF3B activate NMD independently of their EJC binding

In this manuscript, Yi et al. evaluate the role of UPF3 paralogs, UPF3A and UPF3B, in targeting of mRNA to nonsense-mediated decay (NMD). Early studies demonstrated UPF3B as being expressed at higher levels in mammalian cells and playing the major role in eliciting NMD presumably through its binding to EJC complexes and mediating interactions with UPF2, UPF1 and the terminating ribosome; more recent reports, however, have suggested UPF3A can act as a NMD repressor and antagonize NMD as a consequence of its reduced binding to EJCs. To address these conflicting data, the authors here use CRISPR to generate knockout (KO) lines of UPF3 genes in human HCT116 cells and evaluate changes in mRNA levels by RT-qPCR or RNA-Seq. KO of UPF3B (in one case lacking full-length UPF3B but expressing a truncated variant; see below) led to increased levels of a significant number of transcripts harboring a downstream NMD-inducing EJC (compared to those without), but did not alter levels of transcripts targeted to NMD by nature of their long 3' UTR - demonstrating a specific role for UPF3B in EJC-dependent targeting of mRNA to NMD. Importantly, an additional subset of mRNAs in UPF3B KO cells were up-regulated upon siRNA-mediated depletion of the core NMD factor, UPF1, suggesting that NMD was still functional in targeting substrates in the absence of UFP3B. RNA-Seq analysis of cells depleted for UPF3A revealed no significant changes in gene expression, suggesting UPF3A acts as neither an enhancer nor repressor of NMD in these cells. In contrast, depletion of UPF3A in UPF3B KO cells led to a significant increase in transcripts harboring a downstream EJC compared to UPF3B KO alone, providing strong evidence that UPF3A compensates for loss of UPF3B and serves to activate NMD in this system. Consistent with this finding, UPF3A levels and binding to both UPF1 and EJC-proteins, EIF4A3 and CASC3, was shown to be enhanced in the absence of UPF3B. Additional RNA-Seq experiments indicated that UPF1 depletion but not UPF2 knockdown lead to up-regulation of additional EJC-dependent NMD transcripts UPF3A/UPF3B double KO cells suggesting that UPF2 functions only in the presence of UPF3 but UPF1 can target mRNA to NMD in a UPF3-independent manner.

Based on prior findings that CASC3 mediates interaction between UPF3B and the EJC, the authors showed that over-expression of CASC3 (but not a CASC3 mutant) increased association between EIF4A3 with UPF3B (in wild-type) and EIF43 and UPF3A (in UPF3B KO cells), that UPF3B-containing EJCs were highly enriched for CASC3, and that CASC3 KO led to a moderate increase in the levels of a number of EJC-dependent NMD targets as measured by RT-qPCR. Despite these data, CASC3-enriched transcripts did not significantly overlap with transcripts sensitive to UPF3B depletion, and UPF3B-EJC occupancy on an mRNA did not correlate with the efficiency of targeting the mRNA to NMD for that transcript.

Interestingly and consistent with previous reports, over-expression of UPF3A (but not UPF3B)

was shown to inhibit targeting of a β-globin mRNA reporter harboring a premature termination codon to NMD, and replacement of the mid-domain from UPF3B (but not its UPF2 binding domain) into UPF3A abrogated this antagonistic function, possibly through a purported interaction with translation termination factor eRF3 (that was not able to be experimentally confirmed in vivo in this study). In contrast to the plethora of data demonstrating UPF3 binding and association with EJCs, expression of murine UPF3A in UPF3A/UPF3B double KO cells was shown to rescue NMD activity, despite mUPF3A falling to interact with human EJC components by coIP, providing intriguing evidence that UPF3A function in NMD is not mediated through its association with EJCs.

This manuscript would benefit from better presentation and organization of the data. Despite this, the authors provide sufficient evidence that UPF3A acts as a general activator in NMD in HCT116 cells and can complement loss of UPF3B in triggering EJC-dependent NMD substrates to this pathway (except in the case of the PTC-containing β-globin for which over-expression of UPF3A inhibits NMD). While a number of figures are dedicated to demonstrating UPF3 interaction with EJC components (likely mediated by CASC3), this interaction does not appear to correlate with NMD targeting efficiency. Moreover, mouse UPF3A, which fails to exhibit detectable interaction with EJC proteins in HCT116, fully complements cells lacking UPF3A/B, further suggesting that this interaction is dispensable for NMD. How UPF3 functions to mediate EJC-dependent NMD independent of binding the EJC is highly speculated in a confusing model (Figure 7) but not experimentally tested or resolved in this present work.

Specific concerns:

1. The UPF3B knockout cell line #1 expresses a truncated UPF3B polypeptide (lacking amino acids 20-155) at ~10% of full-length UPF3B levels (Fig. 1B, lane 2) that is predicted to maintain its EJC binding domain. This clone should not be considered a UPF3B 'knockout' as the expressed peptide could have ability to interact with EJCs and impact NMD (perhaps in a dominant-negative fashion) or mRNA metabolism generally. This possibility should be discussed and protein interaction blots presented in Fig. 2A and 2C should be expanded to display both full length and truncated UPF3B polypeptides. Given that the author isolated a second UPF3B knockout clone (i.e. #2) that appears to completely disrupt UPF3B expression, it is unclear why this clone was not used for analyzing the role of UPF3B in gene expression throughout.
<u>Response:</u> The reviewer is correct to point out that UPF3B protein lacking its UPF2 binding domain cannot be considered a true "knockout". We now call this mutant "3B$^{\Delta 2BD}$" throughout the manuscript. As predicted by the reviewer, this truncated UPF3B protein indeed still interacts with EJC (Fig EV1B). While we did not specifically test if the truncated polypeptide acts in a dominant-negative manner, similar NMD inhibition observed in this (3B$^{\Delta 2BD}$) and complete UPF3B knockout mutant (3B^{KO}) (qPCR results in Figs EV1C, 1D, EV1I, 2J, EV2H) underscores the similar NMD phenotype of the two mutations. Based on results in Fig EV1C, we had decided early in our studies to use 3B$^{\Delta 2BD}$ cell line for all RNA-Seq analysis. As all main conclusions from RNA-Seq analyses in 3B$^{\Delta 2BD}$ cell line are validated by experiments carried out in 3B^{KO} cell line, we are confident of our results.

2. The authors designate transcripts harboring a EJC >50 nt downstream of the stop codon as "PTC+" (and those lacking as "PTC-"). A more thorough explanation of how these classes of transcripts are defined (how many genes, their expression levels, etc) is needed, especially in clarifying whether they contain a premature termination codon, or, rather, just a downstream EJC.
<u>Response:</u> We have included more details in the methods section about PTC+ and NMD+ (new addition) transcript definitions as requested by the reviewer.

3. The authors demonstrate in Fig. 2B a ~3-fold upregulation of UFP3A protein levels in UPF3B knockout cells, which is consistent with prior published findings. This finding is not recapitulated in input samples in Fig. 2A (lanes 1-4); the authors should address this inconsistency.
Response: *UPF3A was probed with a different antibody in Fig 2A (old Fig 2B), which yields a cross-reacting band of similar size in total extracts but not in IP lanes. We have added a short explanation in the revised text. The upregulation is evident in Fig EV2A where a different UPF3A antibody was used.*

4. Domain swapping experiments presented in Fig. 5 provide evidence that the mid-domain of UPF3A is required for its ability to inhibit targeting of a PTC-containing β-globin reporter to NMD. Based on the data presented, it is inaccurate to conclude that this domain in UPF3B plays an important role in NMD activation; moreover, speculation regarding the function of this domain from UPF3B (i.e. eRF3 binding) is not supported by experiments performed by this group (Fig. S5B). If the authors want to demonstrate an importance for this domain in mediating NMD by UPF3B, then mutational analysis (or replacement with the mid-domain from 3A) needs to be performed.
Response: *We have modified the text in the abstract where we incorrectly interpreted the domain swap experiments in Fig 3 (old Fig 5) to suggest that UPF3B mid-domain is required for full UPF3B activity. Our interpretation is that the UPF3B mid domain swapped into UPF3A makes UPF3A a better NMD activator suggesting that this domain is an important determinant of differences in NMD activity of the two paralogs. We also explicitly state that our speculation that the mid domain may function via eRF3 interaction is based on previous studies and is not supported by our work. The mutational analysis that reviewer suggests is an important next step, which will be addressed in future work.*

5. The manuscript is sufficiently well written but difficult to follow, and the presentation and organization of the data is confusing. Moreover, the lack of a clear and quantitative discussion of the data (i.e. fold-changes listed in the co-IPs and cumulative frequency graphs) leaves the onus to the reader to interpret the significance of each observation and the true findings from this study. In addition, a signifiant amount of the manuscript is devoted to demonstrating UPF3A/B interact with EJC protein components, despite the fact that a key finding is that UPF3 can promote EJC-dependent NMD independent of its association with EJC.
Response: *We have addressed multiple issues listed in this comment as follows:*
Presentation and organization of data: *We have reorganized the results section and order of figures, which we hope improves the flow of the manuscript.*
Quantitative discussion of data: *Where possible, we have quantified western blots for expression/overexpression levels, co-IP efficiency etc, and discuss these quantitative differences in the text. However, there remain a few cases where it is not possible to quantify these data due to technical issues (e.g., Fig 2A, please see the previous comment) or when differences are too obvious (e.g., knockouts). As for differences in cumulative frequency distributions (CDF), we have explained the key visual cue of these plots in the text when CDFs first appear in the manuscript (Fig 1C). As CDF plots compare distributions of a quantity between two populations, it is not possible to summarize the differences between two distributions with a single numerical value.*
Focus on UPF3A/B EJC interaction despite the key finding of EJC independent UPF3 activity: *We agree that EJC independent NMD activation by UPF3 paralogs is our key finding. Still discussion of UPF3-EJC interaction is necessary first to establish the redundant function of the two paralogs and then to address the possible function of UPF3-EJC interaction, which is conserved among multicellulates and is well-known to be consequential for NMD. We hope that reorganization of the results and figures puts UPF3-EJC interaction studies in a better frame of context in the revised manuscript.*

6. The title indicates that neither UPF3A nor UPF3B need to bind EJCs to promote NMD in mammals. While this is directly supported by heterologous data using murine UPF3A, it is unclear what evidence directly supports this conclusion for UPF3B.
<u>Response:</u> We agree with the reviewer's point. We have now performed an experiment where human UPF3B C-terminal domain is replaced by C-term of mouse UPF3A, which has no detectable EJC-binding. This chimera can still fully rescue NMD of the tested targets (new Fig 3D and E). Thus, this data now supports UPF3B's ability to function in NMD without detectable EJC binding.

7. The model presented in Fig. 7 is confusing, highly speculative and presents novel functions for UPF3 not studied or supported by this is better suited for a review.
<u>Response:</u> We have revised Fig 7 and edited the figure legend to more accurately reflect our key findings in the model. We also try to be more explicit about the speculative parts of the model, where the intention is also to provoke readers to think about possible functions of EJC/UPF factors in NMD that is supported by our and others data.

Referee #3:

Yi et al. provide an intriguing study on 2 key factors in the NMD RNA turnover pathway: UPF3A and UPF3B (which I will refer to as 3A and 3B, respectively). There is widespread interest in 3A and 3B because of their association with human disease (e.g., 3B mutations cause human intellectual disability) and the evidence implicating these two factor as key regulators/factors of the NMD pathway.

Using HCT116 colorectal tumor cells for most of their studies, the authors conduct a wide range of impressive experiments to address questions about 3A/3B. They provide evidence for the following main points: First, 3B has a role in NMD, but is not absolutely necessary for NMD (verifying past studies that drew the same conclusion). Second, 3A is largely dispensable for NMD in control cells, but becomes an important back-up when 3B is absent (the first point is not well supported [see below] and the second point verifies the results of past studies). Third, the authors claim they obtained evidence from 3A/3B double knockout (ko) cells that 3A/3B act together in a unique "branch" of NMD, thereby verifying previous work using 3A/3B knockdown (kd) cells. Actually, I think the authors' data indicates instead that 3A/3B do NOT act in a branch-specific way, but rather act together to stimulate the WHOLE NMD pathway (see below). This is not a criticism; indeed, I regard this finding as very important for this field. Fourth, the authors find that 3B does not activate NMD by the means that has been assumed for more than 2 decades. In particular, previous evidence had strongly suggested that 3B is a key tethering molecule that bridges the NMD factor, UPF2, with the exon-junction complex (EJC), the latter of which is a "NMD amplifier" complex bound near exon-exon junctions after RNA splicing. The authors' evidence puts this model in grave doubt - a significant advance for the field. Fifth, the authors find that 3A can act as both an NMD activator and repressor (depending on the context), thus verifying past work (however, their evidence suggests that 3A is mainly a NMD activator in the cells they examined, which differs from a previous study showing that 3A is mainly a NMD repressor in several other cell types). Sixth, the authors identify an intriguing domain in 3A responsible for its repressor activity. Seventh, they investigate the role of the cytoplasmic EJC protein, CASC3, in 3B-dependent NMD, and make several findings, including that CASC3 promotes the association of 3B with the EJC, and that 3B and CASC3 are important for degrading a largely overlapping set of transcripts. Finally, the authors provide evidence that 3A/3B/EJC degrades largely the same transcripts as UPF2. This is important, as it contradicts previous evidence suggesting that UPF2

and the EJC act in different NMD branches, as detailed below. Together with the authors' evidence that 3A/3B does not drive an independent branch of NMD, this MS suggests a re-evaluation of the whole concept that NMD is a branched pathway.

The breadth of the authors' experiments is both an advantage and disadvantage. A key advantage is the authors address many fundamental questions in the field. A disadvantage is the authors sometimes provide insufficient evidence for a given point to make a compelling case. Fortunately, much of this can be rectified with further analysis of the authors' existing data, as detailed below. Another disadvantage of the breadth of studies in this MS is it is often challenging to follow the train of thought through the entire MS. Indeed, this MS does not present a single story. This problem could potentially be rectified by re-organizing the MS and/or moving some sections to a follow-up MS.

Response: As pointed out by this reviewer and reviewer 2 above, we agree that manuscript can be better organized to present a cohesive story. We hope the re-organized results section now improves the flow of the manuscript.

Major concerns:
(A) Limitations of the authors' approach to determine whether NMD is impacted by a given factor. To determine the function of 3A, 3B, and other NMD factors, the authors generated RNAseq datasets from cells deficient (by ko or kd) in the factor of interest. Premature termination codon (PTC)+ and PTC- transcripts were defined from annotated transcripts in the Ensembl database. In accordance the well-established "-50 boundary rule" for EJC-dependent NMD, a PTC+ transcript was defined a mRNA that harbors an in-frame stop codon at least 50 nt from the last exon-exon junction. The PTC+ vs. PTC- data was represented as cumulative distribution function (CDF or MA) plots (e.g., Fig. 1C). While this statistical CDF analysis of PTC+ vs. PTC- transcripts led the authors to draw many interesting conclusions throughout the MS, there are caveats with this analysis, including that not all "PTC+ transcripts" defined by this analysis are necessarily direct NMD targets; e.g., NMD silencer elements have been defined that allow NMD escape, and alternative processing events can obscure interpretation. Conversely, many of the transcripts defined as "PTC-" ARE NMD targets; e.g., many mRNAs with long 3'UTRs are degraded by NMD. Such caveats should be mentioned by the authors. More importantly, the authors often disregard signals other than the EJC that trigger NMD, leading them to sometimes make inappropriate sweeping conclusions about the effect of specific factors on NMD. Examples are given below.
Response: To address this concern, we have now quantified the effect of our manipulations on NMD by an additional approach that expands our analysis beyond PTC+ transcripts (as defined by the –50nt rule). We have defined a new set of NMD sensitive (NMD+) transcripts using an existing dataset where such transcripts are significantly upregulated >1.2-fold upon knockdown of at least two out of three key NMD factors, UPF1, SMG6 and SMG7 (Colombo et al, RNA 2017). This set of transcripts should address both the main concerns above: (i) they are expected to be bona fide NMD targets and hence unlikely to be subjected to regulation by NMD silencer elements. (ii) These transcripts are also not limited to any one particular NMD targeting feature and thus allow us to monitor NMD regardless of the underlying signal. Notably, there is only a partial overlap between NMD+ and PTC+ transcripts. As control, we defined NMD- transcripts as those that change <1.2-fold in either direction upon knockdown of two out of three NMD factors. All key comparisons were repeated using these NMD+/NMD- transcript groups and resulting figures are now included throughout the manuscript (Fig 1E, 1G, 2D, 2F, 2I, 5B, 5E, EV1J, EV5C and EV5D). The outcomes of these new analyses are along the lines of our observations with the PTC+/PTC- group of transcripts, thus lending further support to our main conclusions.

(B) Fig. 1E. Have the 4 RNAs examined by qPCR previously been shown to be EJC-dependent NMD targets, such as by EJC factor kd? Just because a transcript conforms to the -50 boundary rule does mean it is a EJC-dependent NMD target. Also, why were these particular transcripts chosen for qPCR verification? Were they chosen in an unbiased manner?
<u>Response:</u> The genes whose transcripts we chose to monitor via qPCR (4 genes in Fig 1D and 3 genes in Fig EV1I – total 7 genes) were part of a randomly chosen larger set of genes that express both PTC+ and PTC- isoforms. The criteria to pick this larger set was the ability to design PCR primers to specifically amplify/detect their PTC+ and PTC- isoforms. This larger set self-winnowed down to the 7 reported in the manuscript as for some transcripts, PCR oligos did not perform as expected in preliminary tests (e.g., more than 1 PCR product or no PCR product). Thus, these 7 genes tested throughout the manuscript do represent an unbiased group. Although we have not tested PTC+ transcripts for sensitivity to EJC core protein knockdown, Fig 6I does show that a subset is sensitive to CASC3 KO.

(C) Long 3'UTR-dependent NMD. The authors claim that 3B does not (or hardly) degrades mRNAs harboring long 3'UTR (a well-established NMD-inducing signal). While this conclusion may be correct, it would be more fair to the reader if the authors also briefly explained (in the Results or Discussion) the caveats of this conclusion. For example, the analysis was done on ALL transcripts. If focused on 3B and/or 3A/3B-dependent transcripts, a different conclusion might be drawn. Also, the results only pertain to HCT116 colorectal tumor cells. 3B might broadly promote the decay of mRNAs harboring long 3'UTRs in other cell types, including normal cells from other cell lineages.
<u>Response:</u> We have now removed the analysis of long 3'UTR-dependent NMD from the manuscript for two reasons. First, the analysis of NMD+/- group now enables us to extend our work to a wider range of NMD targets beyond the EJC-dependent NMD substrates including those subjected to long 3'UTR dependent NMD. Second, even when we focused our analysis on UPF3A/3B dependent targets (a la new Fig 6G), we did not see any correlation between 3'UTR length and UPF3A/3B sensitivity. Interestingly, a recent publication from the Muhlemann group (Karousis et al., Genome Biol. 2021) also did not observe a 3'UTR length-dependent effect on transcript abundance upon SMG6/7 knockdown in HeLa cells. This raises an important question about long 3'UTRs as a general NMD targeting feature that needs to be examined more rigorously in a separate study. As the reviewer suggests, one needs to carefully consider factors such as cell type, mRNA stability versus indirect transcriptional effects etc.

(D) 3A is inconsequential as a NMD activator or repressor in HCT116 cells. This claim made by the authors is not sufficiently supported, as they only did the total PTC+ vs. PTC- transcript analysis described in A, above, to draw this conclusion. If 3A acts on a specific subset of NMD targets, as shown previously by Shum et al. 2016 in other cell types, this will likely not be revealed by such broad analysis. The authors' data clearly shows that 3A broadly regulates gene expression: Fig. S2D shows that more than 5000 transcripts are significantly dysregulated by 3A kd (with more downregulated than upregulated, consistent with 3A acting as NMD repressor). There are many alternative approaches to better assess the function of 3A in HCT116 cells, including analyzing the effect of 3A on high-confidence NMD targets identified by many past publications. Regardless of the approaches chosen by the authors, it is critical that they rigorously test the possibility that 3A regulates a subset of NMD targets, with one subset being positively regulated by 3A and another subset being negatively regulated by 3A.
<u>Response:</u> We have addressed this concern by two new analyses. First, as detailed in response to comment A by this reviewer, we have extended our analysis to transcripts that are validated to be NMD targets in human cells (NMD+ and their control cohort NMD-). We observe the same general trend that upon UPF3A knockdown there is a very weak shift most of which is toward the positive side in fold-change distribution of NMD+ versus NMD- transcripts (Fig 2D) suggesting

that in parental WT HCT116 cells a general function of UPF3A is as a weak NMD activator. Second, as described in response to a comment from reviewer 1 above, we quantified gene-level changes in expression of a smaller group of ~100 genes that can be considered "high confidence NMD targets" (defined in Supplemental Table S1 in Kurosaki et al (G&D, 2014)). This set includes several targets that have been tested as NMD targets in multiple studies, e.g., ATF3, GADD45B, DNAJB2 and several NMD genes such as UPF1, UPF2, SMG1, SMG5, SMG6. Note that we could not perform transcript level analysis on these genes as they express multiple transcripts with no a priori knowledge of which transcript is likely to be NMD substrate. As shown in a heatmap in Fig EV3A, and in line with our analysis of PTC+ and NMD+ transcripts, UPF3A knockdown causes a weak upregulation of a larger set of these high-confidence NMD genes. Only a smaller set of genes show downregulation upon UPF3A knockdown. In the revised text, we mention that some of these genes could be target of UPF3A's NMD repressor activity but this is likely to be an exception rather than a rule in cell lines we tested.

(E) 3A and 3B are functionally redundant. The authors make a good case that this is correct, based on analysis of 3A kd in 3B-ko cells, as well as 3A-ko/3B-ko (dKO) cells. Furthermore, previous studies have also suggested this is the case. However, the authors analysis would be more complete if they examined not only EJC-dependent targets (by the approach described in A, above) but also analyzed previously identified high-confidence NMD targets, as in D, above. Also, their present analysis does not exclude that 3A ALSO acts as a NMD repressor (for some transcripts) in the context of 3Bko (as shown by Shum et al. in non-malignant cells). This should be dissected. Thus, while the authors analysis of 4 NMD targets by qPCR in Fig. 2H nicely demonstrates that 3A and 3B act redundantly, this does not address whether 3A can ALSO act as a repressor. Thus, they should also seek to verify the downregulation of specific transcripts in response to 3A ko or kd in the context of 3B ko.
Response: As detailed above in responses to A and D, we have now extended our analyses to two other sets of previously validated NMD targets. Again, we observe general trends that are consistent with our earlier conclusions that UPF3A and UPF3B act redundantly. Importantly, such a conclusion is applicable even in UPF3B KO cells where UPF3A knockdown leads to a more robust upregulation of an even larger set of genes (Fig EV3A). Again, we cannot rule out that UPF3A may act as a repressor in a minority of the cases, and indeed now explicitly point this out as a caveat in the text.

(F) Fig. 3G. The authors interpretation of this data is 3B/EJC occupancy does not affect NMD efficiency, but the analysis includes significantly regulated transcripts that are barely misregulated (Log2FC close to 0). Thus, the authors should consider introducing reasonable fold cut-offs. Also, they could focus their analysis only on putative 3A/3B target mRNAs.
Response: We have redone this analysis along the lines suggested by the reviewer. We have compared EJC/UPF3B occupancy of transcripts that are upregulated, downregulated or unchanged upon UPF3A/3B depletion. Such an analysis has revealed a weak but significant correlation between UPF3A/3B dependence and EJC core occupancy but not CASC3 or UPF3B occupancy. This analysis is now described in new Fig 6G and 6H.

(G) Does 3A/3B promotes the decay the same EJC-dependent transcripts as UPF2? The authors claim the answer is "yes" based on reasonable evidence, but nonetheless this seems an overly strong conclusion given that the overlap between the two is not complete. Also, this appears to contradict the results of Gehring et al. Mol Cell '05, a study that obtained evidence that distinct transcripts are targeted by UPF2 vs. EJC factors, leading to the proposal that there are independent UPF2- and EJC-dependent branches. This early work only examined a handful of transcripts, so an apparent discrepancy with the current study is not surprising, but this topic is important conceptually for the field, so it should be discussed.

<u>Response:</u> We have revised the text to soften our conclusion regarding the UPF2 dependence of UPF3-dependent targets. While we have tested UPF3A/3B versus UPF2 dependence, work of Gehring et al Mol Cell 2005 had compared dependence of NMD transcripts on EJC factors RNPS1 and CASC3 with their dependence on UPF2. Due to these differences, we do not think our analysis is comparable to the previous work.

(H) Is there a 3A/3B-independent branch of NMD? Previous 3A/3B kd (in cell lines) and 3B ko (in mice) studies had suggested that there is a 3A/3B-independent branch of NMD. The idea was that this NMD branch degrades transcripts using the central NMD factor, UPF1, but not 3A and 3B. By completely knocking out both 3A and 3B in cells, the authors are in a unique position to rigorously test this model. Interestingly, the authors found that UPF1 kd hardly had any effect on EJC-dependent 3A/3B-independent target mRNAs. In my mind, this and all the other data from the authors suggests that 3A/3B mainly acts as an amplifier of ALL EJC-dependent NMD transcripts, NOT as an amplifier of a specific subset of NMD targets. Thus, I interpret their data as suggesting that 3A/3B does NOT direct a specific branch of NMD. That said, this conclusion is based only on analysis of EJC-dependent transcripts (using the approach described in A, above). The authors should expand their analysis to other transcripts to more fully address whether a 3A/3B-independent NMD branch exists or not.
<u>Response:</u> We agree with the reviewer that the target specificity of UPF3-dependent versus -independent NMD branches is an important question. As suggested by the reviewer, we have expanded this analysis to the NMD+/- group described above. This led us to a somewhat surprising result where we find that, unlike in case of PTC+ transcripts (Fig 5C and 5D), both UPF3-dependent and -independent sets of NMD+ transcripts show a similar upregulation upon additional UPF1 knockdown (Fig 5E). These results suggest that beyond the EJC-dependent NMD, there exist mRNAs that are targeted by either UPF3-dependent or UPF3-independent branches. We have also modified the discussion section to reflect these new results.

(I) Domain-swapping experiments. The authors interesting conclusions from these experiments are based on rescue with plasmids encoding wt 3A, wt 3B, and a panel of 3A/3B swap mutants (Fig. 5C/D). The results are likely to critically depend on expression level of the force expressed plasmids. For physiological relevance, optimally, these should be expressed at endogenous levels. Are they? If not, how much under- or over-expressed?
<u>Response:</u> We have now included western blots that show relative expression levels of rescue constructs expressing full-length UPF3A and UPF3B (Fig EV4B). As shown in the figure, exogenous expression of UPF3B results in ~3.5-fold overexpression in comparison to endogenous UPF3B levels. UPF3A exogenous expression is 18-fold higher than endogenous UPF3A in WT cells, but this still represents ~3-fold overexpression over the total UPF3 (UPF3A+3B) protein in WT cells as the FLAG-UPF3A is the only source of UPF3 in the $UPF3^{DKO}$ cell line. Fig 3C and 3E show that the domain-swap FLAG-tagged chimeras are expressed at similar levels as their FLAG-tagged parent UPF3A and UPF3B counterparts. So we expect domain-swap chimeras to be expressed at ~3-fold higher than endogenous UPF3A+3B levels. We have now added to the text that NMD activity observed in these experiments is under ~3-fold overexpression conditions.

(J) Does 3A act independently of its EJC-binding domain? The authors' domain swapping experiments, as well as experiments with mouse 3A (which has a very weak EJC-interaction domain), suggest that 3A supports NMD without using its EJC-interaction domain. This is an extremely important concept for the field that would optimally be further tested by evaluating a 3A mutant lacking the EJC-interaction domain. However, an alternative is to refer to the Wallmeroth et al. paper, as they conducted such an experiment.

Response: *We have now included a new experiment in Fig 3D and 3E where we have swapped the C-terminal domain of human UPF3B with the C-terminal domain of mouse UPF3A. This chimeric UPF3B shows negligible EJC binding and can still rescue NMD completely (Fig 3E). This new result further bolsters our conclusion about UPF3B (or UPF3A) acting in NMD without much EJC binding. In the discussion, we have also referred to the complete deletion of the C-terminal domain of UPF3B (not UPF3A) in the Wallmeroth et al. paper.*

Minor concerns:

(1) Line 20. The word after "mediated" is "NMD," so the sentence is essentially saying "EJC-mediated nonsense-mediated RNA decay." Replace "mediated" with "dependent" or some similar word.

Response: *We have made this change.*

(2) The authors sometimes use the broad term "UPF3" instead of referring specifically to 3A, 3B paralog, or both. This is ok sometimes (such as on line 46), but not on lines 23, 24, 72, 73, 74, and 76. One line 261, the authors explain they use the general term "UPF3" from then on, but this will be confusing for readers that miss this line. Best to say "both UPF3 paralogs" or "UPF3A/UPF3" or something like that.

Response: *We have carefully reworded the term UPF3 and use the wording suggested by the reviewer.*

(3) UPF3 paralogs is defined twice: on lines 61 and 82.

Response: *We have removed this redundancy.*

(4) Line 99. Gehring et al. does not provide evidence for UPF3B-independent NMD.

Response: *We have removed the reference from this sentence.*

(5) Line 131. Sentence needs revising.

Response: *We have revised the sentence.*

(6) Line 134. Polyadenylation signals do not terminate transcription. Transcription can often proceed to many scattered sites many kb downstream of the polyadenylation signal.

Response: *We have corrected this description.*

(7) The Western blot method seems to be missing from the M&Ms. Also, is there a table of Abs used?

Response: *Methods section has been accordingly updated. A table listing all antibodies used in the study has been included as Appendix Table S5.*

(8) Line 256. Fig. 2H, not G, right? Fig. S2F, not S2G.

Response: *These errors are now fixed in the revision.*

(9) Line 266. Fig. S2I, not S2H, right?

Response: *These errors are now fixed in the revision.*

(10) It is suggested to use the term "control" rather than "wild type" when referring to non-mutant or non-kd cells. WT means "non mutant," and the cell lines used by the authors are tumor cells that presumably have many mutations.

Response: *We agree that malignant cell lines do have several mutations and it is technically inaccurate to describe them as WT. However, we use term "control" to also describe non-targeting siRNAs that are in some cases transfected into parental or "WT" HCT116 cells. Due to the lack of more suitable term and to avoid using "control" for multiple nouns in the same sentence, we have chosen to keep "WT". We have however modified definition of WT as "parental wild-type" (line 129).*

(11) Line 424. "relevant," not "present."
Response: *We have made the change.*

(12) Figs. 1E, 2H, 6C, S1A, S1F, S2G. Not clear what bars are PTC- and PTC+. To rectify, add lines above the - and + symbols that extend to the appropriate bars (e.g., 3 bars for Fig. 1E). This line should be half the length of the line above the gene names. Of course, other solutions are possible.
Response: *We have made the suggested change to all these figures.*

(13) Fig. 6C. Would be more clears if legend indicated that * means values statistically significantly different from EGFP-transfected control cells, which has a value of 1.
(14) The Discussion is generally well written. However, some suggested alterations follow. First, the authors' contributions to the key questions they list are understated in the sentence on lines 455-457. Change to something like: "By generating UPF3A- and UPF3B-deficient human cell lines, we address these important questions using a battery of different approaches." Another issue is the authors do not appear to acknowledge that the reason some of the results they obtained differ from previous studies may be because different cells were examined. For example, most of the results obtained by Shum et al. 2016 were from P19 cells, a non-malignant pluripotent cell line, as well as purified cell types from mice in vivo. This is critical, as there is evidence that NMD acts in a cell type-specific manner. The authors should also acknowledge that Shum et al. did not claim that 3A was only a NMD repressor; they also obtained evidence that 3A is also a NMD activator for some transcripts.
Response: *We have made changes to the Discussion as suggested by the reviewer to indicate the possibility of cell type specific UPF3A repressor function. We have also clarified in the revised manuscript that Shum et al did identify NMD activating role for UPF3A for a smaller subset of genes.*

1st Revision - Editorial Decision **18th Jan 2022**

Thank you for submitting the revised version of your manuscript. We have now received the reports from the three initial referees (see comments below). All referees acknowledge that the manuscript has improved. However, while referee #1 finds that her/his comments have largely been resolved, referees #2 and #3 still have a number of concerns. These issues should addressed in the final version of the manuscript. Therefore, in an exceptional second round of revision, please revise the text accordingly, add the requested information on experimental procedures and expand the discussion as needed. Please also provide a detailed point-by-point response to each of the comments for this revision. In addition, please also address a number of editorial issues that are listed in detail below in the revised version of the manuscript. Please make all edits using the "track changes" option in the manuscript file the data editors have added their notes to (please see below).

Referee #1:

The revised manuscript is considerably improved by the new data, analyses and writing changes. The authors addressed my comments as best as they could. I only have minor correction: Page 16, line 527 should read "Surprisingly, our data shows that mouse UPF3A, which is missing..." (shows instead of shoes). Other than that, I recommend publication of this manuscript in this form.

Referee #2:

Mammalian UPF3A and UPF3B activate NMD independently of their EJC binding

Yi et al.

This revised manuscript by Yi and colleagues addresses many of the concerns raised by this and the other reviewers and is an important contribution to the field. Notwithstanding, there are still a number of clarifications that the authors should consider addressing to increase the clarity and impact of the work.

1. Line 22. It is misleading to state that NMD remains active in cells lacking both UPF3 proteins - this should be qualified to truly represent the data that a subset of transcripts is insensitive to loss of UPF3A/3B but that UPF3 plays a role in NMD for the majority of transcripts targeted to this pathway.
2. Lines 119&120. It is still confusing to conclude that UPF3 proteins promote NMD independently of their association with EJC complexes, but then stating that there is role (albeit secondary) for EJCs in recruiting UPF3 proteins to mRNAs.
3. Line 122. UPF3B is required but not necessary for EJC-dependent NMD. This is very confusing and contradictory.
4. Lines 136-140. It would be of interest to attempt to address why some NMD-sensitive transcripts are sensitive to 3B levels and some are not.
5. Lines 146-152. Description of the RNA-seq data is still very descriptive.
6. Line 151-152. Why does UPF1 KD impact significantly less transcripts in WT than in the 3B delta2BD cells? Is this correlated to KD efficiency over the replicates? Is so, please state.
7. This reviewer is still unclear about the composition of the two datasets analyzed in the RNA-seq analysis (PTC+/- and

NMD+/-). Are PTC+ transcripts also part of the NMD+ dataset?
8. Line 187. Please clarify if 'partially' refers to a reduced level of targeting on all NMD substrates or a smaller subset of substrates. I suspect the latter - which would support the notion of a UPF3-independent pathway for targeting transcripts to NMD.
9. Line 219. 'particularly' should be 'predominantly'.
10. Line 230. 'transcript levels fold changes' is confusing.
11. If UPF3A increases in the absence of UPF3B, why is that not observed in Fig. EV2G (compare lanes 1 to 4 and 5)?
12. Line 306. It is overstated that mouse and rat UPF3A proteins do not interact with EJC proteins. This is true for the heterologous system used here, but has this been tested directly in mouse and rat cells (and in the absence of 3B)?
13. Fig. 4A would benefit from plots to directly indicate decay kinetics of the PTC-containing b-globin mRNA.
14. Lines 348-350. Does this indicate that both the Mid and BC domains of UPF3B can mediate EJC interactions?
15. Line 351. Should be forthright that the observed interaction between UPF3B and eRF3 is in cell extracts and was observed even in the absence of UPF1 or UPF2 (i.e. a functional NMD promoting-complex).
16. The section ending in line 395 is confusing. What is the evidence that EJC's still enhance NMD in the absence of UPF3A/3B?
17. Section starting on line 397. As stated above, it is confusing that the authors want to highlight a finding that CASC3 promotes UPF3A/3B association with EJC to promote EJC-dependent NMD when their main conclusion is that these proteins act independently of their binding to EJC.
18. The model in Fig 7 still fall short in graphically representing the important findings/advances from this work. This reviewer asserts that 'provoking readers to think about possible function of EJC/UPF factors in NMD that is supported by our and others data' is more suited for a perspective or review.

Referee #3:

For the most part, Yi et al. have done an excellent job of responding to the Reviewers' suggestions, including adding new data and new analyses. Overall, their studies involved a huge amount of work, and their interesting results touch on several important issues in the NMD field. Nevertheless, there are some remaining issues, most of which can be dealt with by re-writing.

(1) NMD branches. The notion that NMD is not a single linear pathway, but is instead comprised of branches, is an intriguing notion that has been supported by data for >15 years, but still remains murky. The authors have interesting data on this topic, which is improved by their further analyses since their first submission. That said, there are some pending issues:
(a) Generating a more accurate list of NMD targets. In the original submission, the authors generated and analyzed a list of NMD target mRNAs, which they referred to as premature termination codon + (PTC+) transcripts. These targets are likely to be EJC-dependent, based on their observing the -50 boundary rule. In the revision, the authors report generating a second list of NMD targets, called "NMD+," based on RNAseq data from the Colombo et al. 2017 paper. They also generated a NMD- list, which consists of mRNAs that appear to not be degraded by NMD. Below, we make suggestions on improving these lists. First, it is suggested to re-define the "NMD-" list by only including transcripts shown to be <1.2-fold in response to all 3 NMD factor knockdowns (not just 2 out of 3). Second, as we understand it, NMD+ transcripts were defined as NMD target mRNAs based only on being upregulated in response to NMD factor knockdown. It is well known that many mRNAs upregulated by NMD factor knockdown are indirectly regulated (e.g., all gene targets of a transcriptional activator encoded by a NMD target mRNA would be predicted to be transcriptionally upregulated in response to NMD factor knockdown). While Colombo et al. knocked down several NMD factors, this does not circumvent this problem. Thus, the authors should refine their "NMD+" list by culling all transcripts that do not have at least one other line of evidence for being a direct NMD target (from other papers; no experiments necessary). Alternatively, they could generate a "high confidence" NMD target list from studies other than (or in addition to) Colombo et al. (e.g., studies directly measuring RNA 1/2 life or using elevated phospho-UPF1 as a proxy for being a direct target; indeed, the authors did this for analysis of UPF3A repressor activity - why not use this list for other analyses?). Third, to obtain more meaningful data, the authors should divide their final list of "NMD+" targets into two lists: EJC-dependent and EJC-independent targets (based on the -50 boundary rule). This would allow the authors to potentially draw more clear conclusions about the nature of the mRNA targets degraded by NMD with the assistance of UPF3A and/or UPF3B.
(b) Are there UPF3B-dependent and -independent NMD branches in HCT116 cells? Figs. 1F and 1G clearly show that UPF3B acts as an NMD amplifier - that UPF3B promotes the decay of transcripts acted upon by the central NMD factor UPF1. However, what about NMD target mRNAs not affected by UPF3B KO? Are these upregulated by UPF1 knockdown? If so, we believe this would provide good evidence for a UPF3B-independent branch of NMD. For this analysis, the authors should test both the revised PTC+/PTC- transcript and NMD+/NMD- transcript lists (revised using the approaches suggested above in a).
(c) Previous evidence for UPF3B-dependent and -independent branches. The authors should acknowledge the previous evidence for UPF3B-dependent and -independent branches of NMD in the Introduction and Discussion. Papers identifying NMD targets in UPF3B knockdown and KO cells, including normal (non-malignant and non-immortalized) cells, include: Huang et al. Mol Cell 2011, Nguyen et al. Mol Psych 2012, Huang et al. Mol Psych 2018, Tan et al. eLIFE 2020, and Domingo et al. HMG 2020. Three of these studies analyzed and identified UPF3B-dependent NMD substrates in cells harboring a Upf3b-null mutation: mouse ES cells, purified B and T cells, macrophages, and many adult tissues (Huang et al. 2011), mouse cerebral cortex (Huang et al. 2018), and mouse olfactory neural cell subsets (Tan et al. 2020). It is also stated more than once, including on line 363, that it is not clear whether there are really UPF3B-independent NMD targets, given that only siRNA knockdown

experiments were done. This is not the case.
(d) Are there a UPF3A/B-dependent and independent NMD branches in HCT116 cells? Together, Figs. 5C and 5D suggest the answer is "yes," but for more clarity and depth, the analysis should be repeated with the revised PTC+/PTC- and NMD+/NMD- lists described above.
(e) The Introduction inadequately discusses the functions of UPF3B. As currently written, the Introduction makes it appear that UPF3B (and the UPF3 paralogs in general) are of uncertain importance in higher organisms. Actually, there is considerable evidence for functional and molecular roles for UPF3B, which should at least be summarized. Above, in c, are papers identifying UPF3B-dependent NMD targets. Studies in mice and rodent neural cells have strongly suggested specific biological and molecular functions of UPF3B.
(f) Discussion (lines 585-609). These two paragraphs should be revised in light of two things: (1) possibly revised conclusions, based on the re-analysis suggested, above, in b and d, and (2) the current literature, particularly previous studies on UPF3B- and UPF3A/B-dependent and -independent targets. Some specific suggestions follow: (i) Line 586: this opening sentence unfairly summarizes the literature. And no references are cited. (ii) Line 588: we think this statement is correct, but it needs be better justified in terms of the current literature. (ii) It is important that the authors define in the Discussion (or earlier) exactly what they mean by a "NMD branch." There are situations in which this might be ambiguous. For example, transcripts downregulated by NMD independently of UPF3B in one context may depend on UPF3B in another context (e.g. cell line or cell type). If this is the case, is it fair to call that transcript regulated by the UPF3B-independent branch? It seems a reasonable possibility that what UPF3B does is alter the probability that a given transcript is degraded by NMD. In cell type A, the threshold for decay for that transcript may only be reached in the presence of UPF3B. But in cell type B, the threshold for decay may already be exceeded without UPF3B. If this type of scenario is common, what the authors have done is defined UPF3B- and UPF3A/B-dependent and -independent NMD target mRNAs in HCT116 colorectal cancer cells, but it remains to be seen how these mRNAs are regulated by UPF3B in other cell types, including normal cells. This is not to diminish the authors' work; only to emphasize that they need to put their results in context.

(2) UPF3A function and context. The authors have made a good faith effort to try to resolve the difficult task of elucidating molecular function(s) of UPF3A. They have also made a reasonably strong attempt to reconcile past studies on UPF3A with their own. However, we think a fundamental error they make is not adequately considering context, as we attempt to explain below:
a. A gene paralog is very unlikely to be selected for a property that only exists under rare conditions. The authors' data clearly shows that UPF3A can serve as a NMD activator, a notion that is also supported by past studies. However, the only context in which the authors find that UPF3A acts as a broad NMD activator is when its paralog, UPF3B, is mutated. Because UPF3B is rarely mutated, it thus seems extremely unlikely that UPF3A was selected for during evolution merely because it can serve as a back-up for UPF3B. UPF3A has persisted for a long time, as the available evidence indicates that it was generated by gene duplication at the dawn of the vertebrate lineage, which is estimated to have occurred ~500 million years ago. Thus, while the authors have identified a specific context in which UPF3A is an activator (in response to loss of UPF3B), it is hard to imagine that this function is why this gene has persisted in all vertebrates. It also seems unlikely that this function is physiologically important in normal individuals in the present day. Instead, it is potentially very important for understanding why mutations in UPF3B invariably cause intellectual disability in humans; e.g., the activation of UPF3A NMD-promoting activity in these individuals could potentially be responsible for defects in neural development or function.
b. A tissue- and cell type-specific role of UPF3A is supported by the available evidence. A novel function that UPF3A could be selected for during evolution is to serve as a NMD repressor. Evidence for this was obtained by Shum et al. 2016 in a variety of biological contexts, including normal cells. Why did Yi et al. find some, but limited, evidence for this function? One obvious possibility is that UPF3A acts as a repressor in a cell type- and tissue-specific manner. Consistent with this, UPF3A is only present in vertebrates, which are multicellular organisms with complex cell type- and tissue-specific mechanisms. As further support, its paralog, UPF3B has been shown to be highly regulated and exert its function only in specific tissues and cell types (Huang et al. Mol Cell 2011). A second possible reason for the authors' negative findings is that their evidence comes from a malignant cell line: HCT116 colon cancer cells. While HCT116 cells are probably fine for defining some aspects of NMD, it is reasonable that they would not reproduce all aspects of normal biology, including cell type- and tissue-specific regulatory mechanisms. To the authors' credit, they bring up the possibility that UPF3A is a repressor in some contexts, but it is written as if it was unlikely (e.g. "special conditions" on line 288), which we regard as unfair if one considers that biology is all about context. Indeed, as indicated above, the authors only find that UPF3A has the opposite function (as a NMD activator) in only a very specific (and rare) context.
c. Suggested revisions. It is strongly suggested that the authors revise the MS with the above in mind. Some specific suggestions follow: (i) Line 288: revise this sentence so that it doesn't imply unlikely "special" conditions. Instead, UPF3A repressor function may be cell type- and tissue-specific as indicated above. (ii) Add the important caveat that the results obtained in the authors' study were exclusively performed in malignant cell lines. Contrast this with the previously published findings on UPF3A which were done in normal primary cells, specific cell types in vivo, and immortalized but relatively normal P19 pluripotent cells (which differentiate into neurons and other lineages under non-growth conditions). (iii) Acknowledge that the activator function UPF3A is only observed under the "special condition" that UPF3B is not present (this is mentioned in the revised manuscript, but not as a special condition). (iv) Given that UPF3B mutations cause intellectual disability, expand on the ramifications of this finding in terms of neural disease (in the Introduction, Discussion, and possibly even Abstract). Discussing clinical relevance will likely greatly increase the impact of this MS. (v) Line 496: like the authors' study, the Wallmeroth at al. study showed that UPF3A acts as an NMD activator only in the context of UPF3B loss. Their finding that it only had this activity in this context should be emphasized (here or elsewhere) so that it is clear that this property is unlikely to be the reason that the UPF3A gene exists in vertebrate genomes. (vi) Line 504: the term "artificial conditions" is not accurate. As it says in the next

sentence, UPF3A levels are high in normal cells. (vii) Line 516: P19 cells are not a cell type, they are a cell line. The "cell type" they represent are pluripotent "early embryo" cells. Furthermore, to more fairly represent the previously published data, the authors should list the many mouse Upf3a-null normal cell types that were found to downregulate NMD target mRNAs (primary neural stem cells, primary embryo fibroblasts, FACS-purified spermatoctyes, and olfactory sensory precursor cells and neurons). This data suggests that UPF3A acts as a repressor in all these cell types.
d. Further analysis. For their revision, the authors further examined whether UPF3A act as a repressor in HCT116 cells (in response to a Rev 1 concern, as well as Rev 3, concern D). They found more than 1/3 of dysregulated "high confidence" NMD target mRNAs were downregulated in UPF3A KO cells (Fig. EV3A), which provides some support for the notion that UPF3A can act as a NMD repressor in HCT116 cells. It is suggested that the authors break this down into EJC-dependent and -independent NMD targets to determine whether UPF3A's putative NMD repressor activity in this cell line is directed towards a specific class of NMD target. mRNAs bearing another NMD-inducing feature, long 3'UTRs, could also be examined. If the authors expand their NMD target list (for analysis of UPF3B- and UPF3A/UPF3B-dependent targets in response to critique 1, above), they should also examine these targets.

Minor issues:
Previous Rev 3, comment B. Fig. 1E. Have the 4 RNAs examined by qPCR previously been shown to be EJC-dependent NMD targets, such as by EJC factor knockdown? Just because a transcript conforms to the -50 boundary rule does not mean it is a EJC-dependent NMD target. Also, why were these particular transcripts chosen for qPCR verification? Were they chosen in an unbiased manner?
We are satisfied with the authors' explanation for why the 7 genes were chosen. However, we think it would be highly preferable that mRNA from all 7 genes were directly tested as to whether they are EJC-dependent NMD targets by, for example, knockdown of the EJC factor eIF4A3. Alternatively, if the authors chose not to do this, we request that they at least add the caveat that these mRNAs were not directly tested to be EJC-dependent NMD targets.
Transcript vs. gene level analysis. For the authors' analysis of ~100 high-confidence NMD targets (identified from an earlier study that, in part, used phosphorylated UPF1 as a measure of whether an RNA is a NMD target), they indicated they did gene level, not transcript level, analysis because they did not know which mRNA isoforms are NMD targets. Transcript level analysis is much preferred because it is common for only one isoform transcribed from a given gene to be a NMD target. There are many ways to identify the NMD target isoform(s) such as those that abide by the -50 boundary role and those annotated in other studies to be targeted by NMD.

Abstract. Line 18: "UPF3A/B," not "UPF3B." Line 19: this sentence overly uses the word "dependent." Also, add "an" so it reads "...in an UPF3B...". Line 22: "Surprisingly" seems inappropriate, as it was previously shown by at least 2 papers that UPF3A/UPF3B knockdown does not affect some NMD target mRNAs. Line 25: it is suggested to change the beginning of this sentence to something like "Together, our results demonstrate that..."

Line 122. First section title does not seem accurate. The authors' data indicates that UPF3B is not required for EJC-dependent NMD. A title such as "UPF3B is a NMD amplifier" would accurately depict the contents of this section.

Line 135 incorrectly states initiating premature transcriptional termination prevents expression of full-length transcripts (this was also mentioned in the previous review: Rev 3, minor comment 6). It is suggested to revise to say: "...polyadenylation signal to generate a truncated transcript lacking most UPF3B exons."

Line 307. Since this is a hypothesis, it should be definitive: "...UPF3A will not compensate..."

Line 358. This is a poor title. It is suggested to change to something like "EJC-dependent NMD is promoted by but does not require the UPF3 paralogs."

Line 361. It is suggested to also cite Karam et al. EMBO Reports 2015 here, as this paper also reported evidence of UPF3A/UPF3B-dependent and -independent substrates, albeit not on a genome-wide scale.

Line 462. Add "the" between "without" and "EJC."

Lines 465-467. This sentence undervalues the contributions of the authors data to the field. It is suggested to combine this sentence with the next in the following manner: "...cell lines addresses these questions, leading to an updated model..."

Line 473. Shum et al. also provided evidence that UPF3A is a weak NMD activator.

Line 519. By "conservation," it appears that the authors mean fixation of the two UPF3 paralogs into vertebrate genomes. It is suggested to revise this sentence by saying something along the lines of: "It remains unclear what selective forces permitted the UPF3A paralog to be retained after duplication of the UPF3 gene at the dawn of the vertebrate lineage ~500 million years ago." It is suggested to follow this with a brief discussion of what these selective forces might be.

Previous Rev 3, minor comment 13. There was no response.

<u>Referee #1:</u>

The revised manuscript is considerably improved by the new data, analyses and writing changes. The authors addressed my comments as best as they could. I only have minor correction: Page 16, line 527 should read "Surprisingly, our data shows that mouse UPF3A, which is missing..." (shows instead of shoes). Other than that, I recommend publication of this manuscript in this form.

<u>Response:</u> We thank the reviewer for encouraging words on our work and for catching the spelling error, which we have now corrected.

<u>Referee #2:</u>

Mammalian UPF3A and UPF3B activate NMD independently of their EJC binding

Yi et al.

This revised manuscript by Yi and colleagues addresses many of the concerns raised by this and the other reviewers and is an important contribution to the field. Notwithstanding, there are still a number of clarifications that the authors should consider addressing to increase the clarity and impact of the work.

We thank the reviewer for their constructive comments on our manuscript. Below we address each of their specific concerns.

1. Line 22. It is misleading to state that NMD remains active in cells lacking both UPF3 proteins - this should be qualified to truly represent the data that a subset of transcripts is insensitive to loss of UPF3A/3B but that UPF3 plays a role in NMD for the majority of transcripts targeted to this pathway.

<u>Response:</u> We agree that the sentence in the abstract is not accurate. We have modified this statement to include the qualifier "partially" so that it reads "NMD remains <u>partially</u> active...".

2. Lines 119&120. It is still confusing to conclude that UPF3 proteins promote NMD independently of their association with EJC complexes, but then stating that there is role (albeit secondary) for EJCs in recruiting UPF3 proteins to mRNAs.

<u>Response:</u> Even though our key new results suggest that UPF3 proteins can promote NMD without interacting with EJC, that does not rule out that UPF3-EJC binding cannot play a role in the pathway, albeit a secondary one. We presented this viewpoint in the Discussion section "A revised model for UPF3A/3B function in NMD". Therein, we have cited multiple lines of evidence from the literature that signifies the role of UPF3B-EJC interaction in NMD, even though this interaction may not be essential for UPF3 paralogs to function in NMD. We have also modified the title of the manuscript to better reflect the totality of our work: "Mammalian UPF3A and UPF3B <u>can</u> activate NMD independently of their EJC binding".

3. Line 122. UPF3B is required but not necessary for EJC-dependent NMD. This is very confusing and contradictory.

Response: We have reworded this title: "UPF3B loss of function only partially inhibits the NMD pathway".

4. Lines 136-140. It would be of interest to attempt to address why some NMD-sensitive transcripts are sensitive to 3B levels and some are not.

Response: We have addressed this issue in the revised last section of the discussion.

5. Lines 146-152. Description of the RNA-seq data is still very descriptive.

Response: The descriptive references to RNA-Seq data are based on precise counts of up- and down-regulated transcripts that were shown in Figs EV1E-F. We have now included these numbers in the main text.

6. Line 151-152. Why does UPF1 KD impact significantly less transcripts in WT than in the 3B delta2BD cells? Is this correlated to KD efficiency over the replicates? Is so, please state.

Response: We observe very similar UPF1 KD efficiency in WT and $3B^{\Delta 2BD}$ cells as shown in updated Fig EV1D. Our interpretation of fewer affected transcripts in UPF1 KD in WT cells as compared to $3B^{\Delta 2BD}$ cells is as follows: In WT cells, UPF1 knockdown is expected to upregulate all NMD targeted mRNAs. However, the degree of effect on NMD targets will be variable due to factors such as target expression level, UPF1 sensitivity of transcripts (some UPF1 is still present), etc. So, some NMD targets may be affected but not show a significant change. In $3B^{\Delta 2BD}$ cells, a subset of NMD targets is already significantly upregulated and several others may be teetering close to the threshold of NMD inhibition. When UPF1 is knocked down in these cells, a larger number of transcripts are inefficiently targeted to NMD, most likely because two different NMD factors being depleted at the same time.

7. This reviewer is still unclear about the composition of the two datasets analyzed in the RNA-seq analysis (PTC+/- and NMD+/-). Are PTC+ transcripts also part of the NMD+ dataset?

Response: We have tried to further explain our approach to define the two sets of transcripts in Materials and Methods section. Therein, we have also included the overlap between PTC+ and NMD+ datasets (379 transcripts). We have now included spreadsheets with all DESeq2 comparisons where PTC +/- or NMD +/- status of each transcript is also indicated (source data files for Figs 1, 2, 5, EV1, and EV5).

8. Line 187. Please clarify if 'partially' refers to a reduced level of targeting on all NMD substrates or a smaller subset of substrates. I suspect the latter - which would support the notion of a UPF3-independent pathway for targeting transcripts to NMD.

Response: *We have updated Fig 5 to address the issue of whether UPF3A/3B-independent NMD represents reduced targeting of all substrates or targets a smaller set of substrates. Fig 5C shows two possible models for "partial" UPF3A/B-dependent and -independent NMD activity. These models are tested in Fig 5D-F. The answer is that we observe evidence for both models, as seen in Figs 5D-F. We have made changes to the results section to first present these models and then the data examining them. We have also further expanded on the interpretation and implications of these findings in the "UPF3A/B-dependent and -independent NMD branches" section of the Discussion.*

9. Line 219. 'particularly' should be 'predominantly'.

Response: *We have made this change.*

10. Line 230. 'transcript levels fold changes' is confusing.

Response: *We have reworded this phrase to make it clear.*

11. If UPF3A increases in the absence of UPF3B, why is that not observed in Fig. EV2G (compare lanes 1 to 4 and 5)?

Response: *We have included new western blots in Fig EV2G along with quantification of three biological replicates where UPF3A increase is evident in UPF3B KO cells.*

12. Line 306. It is overstated that mouse and rat UPF3A proteins do not interact with EJC proteins. This is true for the heterologous system used here, but has this been tested directly in mouse and rat cells (and in the absence of 3B)?

Response: *This is a good point as, to our knowledge, the EJC-UPF3A interaction has not been rigorously tested directly in rodent cells. We have added "may" as a qualifier to the statement.*

13. Fig. 4A would benefit from plots to directly indicate decay kinetics of the PTC-containing b-globin mRNA.

Response: *We have included these plots in Fig EV3B and Fig EV4C. We also want to add that in Fig 4 in the previous submission, there was an unintentional error in half-life calculations for one of the replicates (except in the case of UPF3B OE). We have re-calculated half-lives for this replicate (#2) and also the overall average half-lives to correct this error. The overall result and conclusions for this experiment remain the same.*

14. Lines 348-350. Does this indicate that both the Mid and BC domains of UPF3B can mediate EJC interactions?

Response: *Yes, the reviewer is correct about our conclusion, which is stated on lines 434-437 (lines 353-356 in the previous revision).*

15. Line 351. Should be forthright that the observed interaction between UPF3B and eRF3 is in cell extracts and was observed even in the absence of UPF1 or UPF2 (i.e. a functional NMD promoting-complex).

Response: The previous UPF3B-eRF3 interactions were reported both from cell extracts and from in vitro pull-down assays using recombinant proteins. We have modified the text to more accurately reflect the previous results.

16. The section ending in line 395 is confusing. What is the evidence that EJC's still enhance NMD in the absence of UPF3A/3B?

Response: As noted in response to comment 8 above, we have modified Fig 5 and its description in the text to address the non-essential but stimulatory role of UPF3 paralogs in NMD. The evidence that the EJC still enhances NMD in the absence of UPF3A/3B is in Fig 5D where a group of PTC+ mRNAs (and hence probable EJC-dependent NMD substrates), but not their PTC- counterparts, are further upregulated upon UPF1 knockdown in $UPF3^{DKO}$ cells. Thus, for these transcripts, NMD is likely triggered by downstream splicing and hence the EJC in a UPF3 independent manner.

17. Section starting on line 397. As stated above, it is confusing that the authors want to highlight a finding that CASC3 promotes UPF3A/3B association with EJC to promote EJC-dependent NMD when their main conclusion is that these proteins act independently of their binding to EJC.

Response: Please see response to comment 2 above.

18. The model in Fig 7 still fall short in graphically representing the important findings/advances from this work. This reviewer asserts that 'provoking readers to think about possible function of EJC/UPF factors in NMD that is supported by our and others data' is more suited for a perspective or review.

Response: The reviewer did not identify specific issues with Fig 7, so we have made some minor changes to the figure and the Fig legend to further clarify the model and its description. Here we describe how Fig 7 graphically represents important findings/advances from our work:

-Fig 7A summarizes the early step where EJC interaction with UPF3B (or 3A) can recruit these proteins to mRNA to position them to enhance NMD (supported by data from Fig 6 and also Fig 2).

-Fig 7B (middle schematic) shows UPF3 proteins functioning during recognition of premature termination event. This occurs independently of UPF3-EJC binding (supported by Fig 3), so we do not show any direct contact between UPF3B and EJC. This is unlike current models that show UPF3 as a “bridge”. Again, 3A can replace 3B in this function (supported by Figs 2 and 3). The red dotted arrow with a question mark indicates an unknown mode of communication between UPF1 and the downstream EJC. Presence of an EJC in 3’UTR is still likely to enhance NMD (supported by Figs 5A, 5B and 5D).

-Fig 7C represents UPF3A/3B-independent NMD (supported by Figs 5A, 5B, 5D and 5E), which may also be UPF2-independent (supported by Fig EV5D and EV5E).

Referee #3:

For the most part, Yi et al. have done an excellent job of responding to the Reviewers' suggestions, including adding new data and new analyses. Overall, their studies involved a huge amount of work, and their interesting results touch on several important issues in the NMD field. Nevertheless, there are some remaining issues, most of which can be dealt with by re-writing.

We thank the reviewer for their constructive comments on our manuscript. Below is a point-by-point response to how we have addressed each of their concerns.

(1) NMD branches. The notion that NMD is not a single linear pathway, but is instead comprised of branches, is an intriguing notion that has been supported by data for >15 years, but still remains murky. The authors have interesting data on this topic, which is improved by their further analyses since their first submission. That said, there are some pending issues:
(a) Generating a more accurate list of NMD targets. In the original submission, the authors generated and analyzed a list of NMD target mRNAs, which they referred to as premature termination codon + (PTC+) transcripts. These targets are likely to be EJC-dependent, based on their observing the -50 boundary rule. In the revision, the authors report generating a second list of NMD targets, called "NMD+," based on RNAseq data from the Colombo et al. 2017 paper. They also generated a NMD- list, which consists of mRNAs that appear to not be degraded by NMD. Below, we make suggestions on improving these lists.

First, it is suggested to re-define the "NMD-" list by only including transcripts shown to be <1.2-fold in response to all 3 NMD factor knockdowns (not just 2 out of 3).

Response (1a-i): After careful consideration, we have decided not to make this change. This is an arbitrary decision that will have only a very minor effect on the negative control data set (NMD-). If anything, it will make the control NMD- group even less likely to contain any false negatives and hence will further enhance the differences, an important point that we now explicitly mention in the revised manuscript. We do not expect our results or conclusions to change significantly.

Second, as we understand it, NMD+ transcripts were defined as NMD target mRNAs based only on being upregulated in response to NMD factor knockdown. It is well known that many mRNAs upregulated by NMD factor knockdown are indirectly regulated (e.g., all gene targets of a transcriptional activator encoded by a NMD target mRNA would be predicted to be transcriptionally upregulated in response to NMD factor knockdown). While Colombo et al. knocked down several NMD factors, this does not circumvent this problem. Thus, the authors should refine their "NMD+" list by culling all transcripts that do not have at least one other line of evidence for being a direct NMD target (from other papers; no experiments necessary). Alternatively, they could generate a "high confidence" NMD target list from studies other than (or in addition to) Colombo et al. (e.g., studies directly measuring RNA 1/2 life or using elevated phospho-UPF1 as a proxy for being a direct target; indeed, the authors did this for analysis of UPF3A repressor activity - why not use this list for other analyses?).

Response (1a-ii): *We agree with the referee that the indirect effects of NMD factor knockdown is an important issue to consider. It is for this reason we compare potential NMD targeted PTC+ transcripts with non-NMD target PTC- isoforms from the same set of genes. Indirect effects are likely to affect both PTC+ and PTC- transcripts whereas effects on NMD are expected to impact only PTC+ transcripts. We had pointed out this important issue for the PTC+/- pair in the previous version of the manuscript.*

Following the referee's suggestion, in the previous revision we had also addressed this issue in Fig EV3A by examining the effect of our manipulations on genes that were previously determined to be more likely direct targets of NMD activity due to their increased mRNA half-life upon UPF1 depletion and their mRNA enrichment with phospho-UPF1. This analysis yielded results consistent with our results with NMD+ and PTC+ datasets.

We have further attempted to improve our analysis in Fig EV3A by restricting to only those NMD+ transcripts that are also supported by "direct" measures of NMD noted above. We want to note that these comparisons are hindered by several limitations that result in very short list of candidates to examine. First, the datasets in these comparisons (published and our own datasets) are obtained from different cell lines with different gene expression patterns. Due to this reason, the overlaps between significantly affected genes in different datasets are usually small. Thus, in Fig EV3A in the previous revision we were able to query only ~90 genes, which came from Kurosaki et al., PNAS 2014. We have now combined the list from Kurosaki et al. with another similar study (Imamachi et al., Genome Res 2017) that also defined NMD targets based on their increased mRNA half-life upon UPF1 depletion and mRNA enrichment with phospho-UPF1. This gene count of the combined list goes up to ~300 genes but we also note that there are only 30 genes common between datasets from Kurosaki et al. (105 genes) and Imamachi et al. (247 genes). Second, the published datasets listed above provide gene level quantification as they have insufficient sequencing depth to achieve transcript level quantification. This is why in Fig EV3A in the previous revision we performed only gene level analysis. Now when we overlap our NMD+ transcript set with the list of ~300 genes (union of Kurosaki et al. and Imamachi et al.), we are left with 93 transcripts, which we define as "stringent" NMD targets. We have updated Fig EV3A to show these 93 transcripts for the four key comparisons that represent the foundation of our study. Importantly, the overall results remain the same.

To further confirm our results, we have also compared our PTC+ and NMD+ transcripts to those that are defined as NMD targets by Lykke-Andersen et al. (Genes & Dev 2014) on the basis of NMD-dependent SMG6 endo-cleavage or decapping of mRNAs. Following this comparison, ~1200 PTC+ transcripts qualified as "stringent" NMD targets. Next, we compared fold changes of these PTC+ transcripts to their PTC- counterparts in the four key DESeq2 analyses. Strikingly, we obtained almost identical results as with the larger PTC+ list (Response to reviewers Fig 1A). Possibly due to the limitations noted above, comparison of NMD targets from Lykke-Andersen et al. with the NMD+ group yielded only ~240 NMD+ transcripts and ~120 corresponding NMD- transcripts. Again, when we compared these NMD+ and NMD- transcripts in the four key DESeq2 analyses, our results remain the same (Response to reviewers Fig 1B). As these new analyses confirm our previous results using the larger PTC+ and NMD+ datasets, we have decided not to include the results from "stringent" PTC+ and NMD+ sets in the revised manuscript.

Third, to obtain more meaningful data, the authors should divide their final list of "NMD+" targets into two lists: EJC-dependent and EJC-independent targets (based on the -50 boundary rule).

This would allow the authors to potentially draw more clear conclusions about the nature of the mRNA targets degraded by NMD with the assistance of UPF3A and/or UPF3B.

Response (1a-iii): While it will be interesting to evaluate the need for UPF3A/3B for EJC-dependent and EJC-independent NMD substrates, comparisons of our NMD+ lists with previously published NMD lists yields a limited set of transcripts to analyze, as noted in the response to the previous comment. So, we are not sure that meaningful conclusions can be drawn by further stratification of these small lists.

(b) Are there UPF3B-dependent and -independent NMD branches in HCT116 cells? Figs. 1F and 1G clearly show that UPF3B acts as an NMD amplifier - that UPF3B promotes the decay of transcripts acted upon by the central NMD factor UPF1. However, what about NMD target mRNAs not affected by UPF3B KO? Are these upregulated by UPF1 knockdown? If so, we believe this would provide good evidence for a UPF3B-independent branch of NMD. For this analysis, the authors should test both the revised PTC+/PTC- transcript and NMD+/NMD- transcript lists (revised using the approaches suggested above in a).

Response: Fig 5 already tests the main idea suggested in the above comment. Importantly, in our opinion, we cannot exclusively focus on NMD that is dependent or independent of only UPF3B because, as shown in Fig EV2I, in HCT116 cells UPF3A acts on a remarkably similar set of transcripts as UPF3B. If an mRNA is UPF3B-dependent, it is also very likely to be UPF3A-dependent, and vice versa. So, the analysis specifically requested in this comment cannot be performed. We have already analyzed the UPF3A+B-dependent and -independent NMD targets in Fig 5A and B.

(c) Previous evidence for UPF3B-dependent and -independent branches. The authors should acknowledge the previous evidence for UPF3B-dependent and -independent branches of NMD in the Introduction and Discussion. Papers identifying NMD targets in UPF3B knockdown and KO cells, including normal (non-malignant and non-immortalized) cells, include: Huang et al. Mol Cell 2011, Nguyen et al. Mol Psych 2012, Huang et al. Mol Psych 2018, Tan et al. eLIFE 2020, and Domingo et al. HMG 2020. Three of these studies analyzed and identified UPF3B-dependent NMD substrates in cells harboring a Upf3b-null mutation: mouse ES cells, purified B and T cells, macrophages, and many adult tissues (Huang et al. 2011), mouse cerebral cortex (Huang et al. 2018), and mouse olfactory neural cell subsets (Tan et al. 2020). It is also stated more than once, including on line 363, that it is not clear whether there are really UPF3B-independent NMD targets, given that only siRNA knockdown experiments were done. This is not the case.

Response: Here is our rationale why we have focused on a subset of studies that have identified UPF3B NMD targets: Chan et al., EMBO J 2007 also used UPF1 knockdown to assess UPF3B dependence vs independence of a subset of NMD targets whereas Huang et al. Mol Cell 2011 tested UPF3B dependence of a subset of NMD targets in different cell types. So, inferences of UPF3B dependence versus independence can be clearly drawn from these two studies. We have added Karam et al. EMBO Rep 2015 to this list upon the reviewers' suggestion. Other studies cited in the comment above focus on mRNAs that change upon UPF3B loss but do not assess the possibility of UPF3B independence (e.g., by a more widespread inhibition of the NMD pathway). It is for this reason we specifically focused on Chan

et al. on line 363 (line 443 in revised manuscript). In the Introduction of the revised manuscript, we have now included a brief discussion of all the suggested works in the comment above.

(d) Are there a UPF3A/B-dependent and independent NMD branches in HCT116 cells? Together, Figs. 5C and 5D suggest the answer is "yes," but for more clarity and depth, the analysis should be repeated with the revised PTC+/PTC- and NMD+/NMD- lists described above.

<u>Response:</u> We have not done this analysis because, as noted above in the response to 1a, this will require further stratification of a short list of "stringent" NMD+ transcripts.

(e) The Introduction inadequately discusses the functions of UPF3B. As currently written, the Introduction makes it appear that UPF3B (and the UPF3 paralogs in general) are of uncertain importance in higher organisms. Actually, there is considerable evidence for functional and molecular roles for UPF3B, which should at least be summarized. Above, in c, are papers identifying UPF3B-dependent NMD targets. Studies in mice and rodent neural cells have strongly suggested specific biological and molecular functions of UPF3B.

<u>Response:</u> We summarized UPF3B functions including its important biological roles in the Introduction but from the viewpoint that unlike the other two UPF factors, UPF3B is non-essential for NMD and organismal viability. This is intentional as we want to raise the key question addressed in the manuscript: how NMD can work independently of UPF3B. In the revised introduction, we have further clarified that, despite being non-essential, UPF3B has important biological functions. We have also added suggested references that support biological and molecular functions of UPF3B in specific processes.

(f) Discussion (lines 585-609). These two paragraphs should be revised in light of two things: (1) possibly revised conclusions, based on the re-analysis suggested, above, in b and d, and

<u>Response:</u> Some suggested analysis that we have done has not changed any conclusions. As explained above in responses to (d) above, other analysis was not performed. So, the conclusions that form the basis of the revised discussion remain the same.

(2) the current literature, particularly previous studies on UPF3B- and UPF3A/B-dependent and -independent targets. Some specific suggestions follow: (i) Line 586: this opening sentence unfairly summarizes the literature. And no references are cited.

<u>Response:</u> We have modified the sentence. As the sentence refers to a topic already covered (with relevant papers cited) in the Introduction, we did not include references but have done so in the revision.

(ii) Line 588: we think this statement is correct, but it needs be better justified in terms of the current literature.

<u>Response:</u> We have revised the sentence and include reference to previous studies that attempted to assess NMD activity by depleting both UPF3A and UPF3B, like our work.

(ii) It is important that the authors define in the Discussion (or earlier) exactly what they mean by a "NMD branch." There are situations in which this might be ambiguous. For example, transcripts downregulated by NMD independently of UPF3B in one context may depend on UPF3B in another context (e.g. cell line or cell type). If this is the case, is it fair to call that transcript regulated by the UPF3B-independent branch? It seems a reasonable possibility that what UPF3B does is alter the probability that a given transcript is degraded by NMD. In cell type A, the threshold for decay for that transcript may only be reached in the presence of UPF3B. But in cell type B, the threshold for decay may already be exceeded without UPF3B. If this type of scenario is common, what the authors have done is defined UPF3B- and UPF3A/B-dependent and -independent NMD target mRNAs in HCT116 colorectal cancer cells, but it remains to be seen how these mRNAs are regulated by UPF3B in other cell types, including normal cells. This is not to diminish the authors' work; only to emphasize that they need to put their results in context.

Response: We have now defined the idea of "NMD branches" in the Introduction and expanded on the idea in results section. We have also incorporated the possibility of cell type specific nature of NMD branches/NMD factor thresholds in the discussion.

(2) UPF3A function and context. The authors have made a good faith effort to try to resolve the difficult task of elucidating molecular function(s) of UPF3A. They have also made a reasonably strong attempt to reconcile past studies on UPF3A with their own. However, we think a fundamental error they make is not adequately considering context, as we attempt to explain below:

a. A gene paralog is very unlikely to be selected for a property that only exists under rare conditions. The authors' data clearly shows that UPF3A can serve as a NMD activator, a notion that is also supported by past studies. However, the only context in which the authors find that UPF3A acts as a broad NMD activator is when its paralog, UPF3B, is mutated. Because UPF3B is rarely mutated, it thus seems extremely unlikely that UPF3A was selected for during evolution merely because it can serve as a back-up for UPF3B. UPF3A has persisted for a long time, as the available evidence indicates that it was generated by gene duplication at the dawn of the vertebrate lineage, which is estimated to have occurred ~500 million years ago. Thus, while the authors have identified a specific context in which UPF3A is an activator (in response to loss of UPF3B), it is hard to imagine that this function is why this gene has persisted in all vertebrates. It also seems unlikely that this function is physiologically important in normal individuals in the present day. Instead, it is potentially very important for understanding why mutations in UPF3B invariably cause intellectual disability in humans; e.g., the activation of UPF3A NMD-promoting activity in these individuals could potentially be responsible for defects in neural development or function.

b. A tissue- and cell type-specific role of UPF3A is supported by the available evidence. A novel function that UPF3A could be selected for during evolution is to serve as a NMD repressor. Evidence for this was obtained by Shum et al. 2016 in a variety of biological contexts, including normal cells. Why did Yi et al. find some, but limited, evidence for this function? One obvious possibility is that UPF3A acts as a repressor in a cell type- and tissue-specific manner. Consistent with this, UPF3A is only present in vertebrates, which are multicellular organisms with complex cell type- and tissue-specific mechanisms. As further support, its paralog, UPF3B has been shown to be highly regulated and exert its function only in specific tissues and cell types (Huang et al. Mol Cell 2011). A second possible reason for the authors' negative findings

is that their evidence comes from a malignant cell line: HCT116 colon cancer cells. While HCT116 cells are probably fine for defining some aspects of NMD, it is reasonable that they would not reproduce all aspects of normal biology, including cell type- and tissue-specific regulatory mechanisms. To the authors' credit, they bring up the possibility that UPF3A is a repressor in some contexts, but it is written as if it was unlikely (e.g. "special conditions" on line 288), which we regard as unfair if one considers that biology is all about context. Indeed, as indicated above, the authors only find that UPF3A has the opposite function (as a NMD activator) in only a very specific (and rare) context.

Response to 2a+b:

Conservation of the UPF3A gene: We believe the current body of evidence about UPF3A function, including from our own manuscript, is insufficient to make definitive conclusions about the evolutionary forces that have shaped co-existence of the two UPF3 paralogs. There are several theories for conservation of gene paralogs that invoke various possible mechanisms for this phenomenon (Introduction of Assis and Bachtrog, PNAS 2013): conservation (retention of ancestral function in both copies), sub-functionalization (both paralogs required to preserve ancestral function), neo-functionalization (one paralog takes a new function, such as UPF3B-antagonistic function for UPF3A as proposed by Shum et al.), and specialization (sub- and neo-functionalization acting in concert). In our opinion, UPF3A function has been investigated in only very few contexts and that too in many cases only at a limited scale, at genome-wide level in human and mouse cell lines and for a few candidate genes in primary mouse cells. Currently available data argue for UPF3A function as both an NMD activator and repressor. Until more exhaustive analysis of UPF3A function is undertaken in multiple contexts (different organisms, cell types, tissues, developmental stages etc.), it is not possible to conclude which specific mechanism is at work for UPF3A co-existence with UPF3B. For example, it is still possible that in a yet unknown context, NMD activating function of both UPF3A and UPF3B is needed to achieve proper "UPF3" dosage for normal function. In another context that is already noted in the discussion, the UPF3B gene, which is located on the X chromosome, is possibly silenced during meiotic X-chromosome inactivation. Current data do not preclude that in a specific stage/cell type during spermatogenesis, UPF3A also acts as NMD activator where it is the predominant source of UPF3 due to silencing of UPF3B. The data supporting UPF3A acting as a repressor obtained by Shum et al. tested only 7 NMD targets in this cell type and of these 4-5 support UPF3A function as a repressor. Therefore, we believe that it is not prudent to make such a strong claim about which activity of UPF3A has contributed to its retention in vertebrate genomes.

UPF3A may also be retained in vertebrate genomes due to a non-NMD function. We have discussed this possibility of UPF3A function outside NMD (line 297-299) given its clear impact on gene expression (Fig EV2E) but not on NMD (Fig 2C and 2D).

c. Suggested revisions. It is strongly suggested that the authors revise the MS with the above in mind. Some specific suggestions follow: (i) Line 288: revise this sentence so that it doesn't imply unlikely "special" conditions. Instead, UPF3A repressor function may be cell type- and tissue-specific as indicated above.

Response: We agree that from currently available data on UPF3A function in the contexts tested here and elsewhere, it is not possible to tell if one condition/cell-type is special or outlier as compared to others. So, we have revised this sentence.

(ii) Add the important caveat that the results obtained in the authors' study were exclusively performed in malignant cell lines. Contrast this with the previously published findings on UPF3A which were done in normal primary cells, specific cell types in vivo, and immortalized but relatively normal P19 pluripotent cells (which differentiate into neurons and other lineages under non-growth conditions).

Response: We have discussed this caveat in the revised manuscript. To present a balanced view, we have also discussed other caveats of such a limited number of NMD targets tested in most mouse primary cell types in Shum et al. We have also noted that differences in our findings as compared to the previous work could also stem from mouse versus human cells.

(iii) Acknowledge that the activator function UPF3A is only observed under the "special condition" that UPF3B is not present (this is mentioned in the revised manuscript, but not as a special condition).

Response: As noted above, we don't believe that from the limited evidence available for UPF3A function one can conclude that one condition is more special than the other. For example, all studies combined in total have looked at only a select cell types from two organisms. In many of these cell types, only a handful of possible NMD targets have been tested. Thus, we have not made this change.

(iv) Given that UPF3B mutations cause intellectual disability, expand on the ramifications of this finding in terms of neural disease (in the Introduction, Discussion, and possibly even Abstract). Discussing clinical relevance will likely greatly increase the impact of this MS.

Response: We have expanded on this topic in the discussion.

(v) Line 496: like the authors' study, the Wallmeroth at al. study showed that UPF3A acts as an NMD activator only in the context of UPF3B loss. Their finding that it only had this activity in this context should be emphasized (here or elsewhere) so that it is clear that this property is unlikely to be the reason that the UPF3A gene exists in vertebrate genomes.

Response: We already stated that UPF3A's NMD activator function becomes obvious only when UPF3B is absent (lines 296-297). However, as stated above in response to 2a+b, it is not possible based on the current data to rule out if UPF3A NMD activation does not contribute to its presence in vertebrate genomes.

(vi) Line 504: the term "artificial conditions" is not accurate. As it says in the next sentence, UPF3A levels are high in normal cells.

Response: The term "artificial conditions" strictly referred to our overexpression of UPF3A in HeLa cells, and we did not intend for the connotation of this term to carry over to the next sentence. We have now reworded this sentence.

(vii) Line 516: P19 cells are not a cell type, they are a cell line. The "cell type" they represent are pluripotent "early embryo" cells. Furthermore, to more fairly represent the previously published data, the authors should list the many mouse Upf3a-null normal cell types that were found to downregulate NMD target mRNAs (primary neural stem cells, primary embryo fibroblasts, FACS-purified spermatoctyes, and olfactory sensory precursor cells and neurons). This data suggests that UPF3A acts as a repressor in all these cell types.

Response: *We have deleted "types" from the sentence and changed the preceding word to "cells". Also, as suggested, we have included in Discussion the primary cell types where UPF3A function as an NMD repressor was shown.*

d. Further analysis. For their revision, the authors further examined whether UPF3A act as a repressor in HCT116 cells (in response to a Rev 1 concern, as well as Rev 3, concern D). They found more than 1/3 of dysregulated "high confidence" NMD target mRNAs were downregulated in UPF3A KO cells (Fig. EV3A), which provides some support for the notion that UPF3A can act as a NMD repressor in HCT116 cells. It is suggested that the authors break this down into EJC-dependent and -independent NMD targets to determine whether UPF3A's putative NMD repressor activity in this cell line is directed towards a specific class of NMD target. mRNAs bearing another NMD-inducing feature, long 3'UTRs, could also be examined. If the authors expand their NMD target list (for analysis of UPF3B- and UPF3A/UPF3B-dependent targets in response to critique 1, above), they should also examine these targets.

Response: *In Fig EV3A, there are only a handful of targets that show downregulation upon UPF3A knockdown in all conditions tested (transcripts toward the bottom of the heatmaps). The same was true in the gene-level heatmap in Fig EV3A in the previous revision. Even if we consider only UPF3A knockdown condition alone, we believe the number of targets (18 transcripts in the new Fig EV3A; 26 genes in the previous version) is too small to further divide into groups as suggested. Moreover, as noted above, these datasets have other confounding variables that could lead to change in one or the other direction. So, we do not think that reliable conclusions can be made from such an analysis.*

Minor issues:
Previous Rev 3, comment B. Fig. 1E. Have the 4 RNAs examined by qPCR previously been shown to be EJC-dependent NMD targets, such as by EJC factor knockdown? Just because a transcript conforms to the -50 boundary rule does not mean it is a EJC-dependent NMD target. Also, why were these particular transcripts chosen for qPCR verification? Were they chosen in an unbiased manner?
We are satisfied with the authors' explanation for why the 7 genes were chosen. However, we think it would be highly preferable that mRNA from all 7 genes were directly tested as to whether they are EJC-dependent NMD targets by, for example, knockdown of the EJC factor eIF4A3. Alternatively, if the authors chose not to do this, we request that they at least add the caveat that these mRNAs were not directly tested to be EJC-dependent NMD targets.

Response: *We have included the caveat that it remains to be tested if qPCR targets are also sensitive to EJC factor knockdown.*

Transcript vs. gene level analysis. For the authors' analysis of ~100 high-confidence NMD targets (identified from an earlier study that, in part, used phosphorylated UPF1 as a measure of whether an RNA is a NMD target), they indicated they did gene level, not transcript level, analysis because they did not know which mRNA isoforms are NMD targets. Transcript level analysis is much preferred because it is common for only one isoform transcribed from a given gene to be a NMD target. There are many ways to identify the NMD target isoform(s) such as those that abide by the -50 boundary role and those annotated in other studies to be targeted by NMD.

Response: *We agree with the referee that transcript level analysis is preferable. That is why we have used the PTC+/PTC- list as presence of downstream introns is the most well-characterized feature at the transcript level that defines a potential NMD target. For the same reasons, we strived to specifically measure transcript level abundance for the NMD+/NMD- list that we added in the previous revision. As noted in response to comment 1, we were able to obtain a list of 93 stringent NMD+ transcripts that we have used to update Fig EV3A in the current revision.*

Abstract. Line 18: "UPF3A/B," not "UPF3B." Line 19: this sentence overly uses the word "dependent." Also, add "an" so it reads "...in an UPF3B...". Line 22: "Surprisingly" seems inappropriate, as it was previously shown by at least 2 papers that UPF3A/UPF3B knockdown does not affect some NMD target mRNAs. Line 25: it is suggested to change the beginning of this sentence to something like "Together, our results demonstrate that..."

Response: *We have made all the suggested changes.*

Line 122. First section title does not seem accurate. The authors' data indicates that UPF3B is not required for EJC-dependent NMD. A title such as "UPF3B is a NMD amplifier" would accurately depict the contents of this section.

Response: *We have changed the title to "UPF3B loss of function only partially inhibits the NMD pathway".*

Line 135 incorrectly states initiating premature transcriptional termination prevents expression of full-length transcripts (this was also mentioned in the previous review: Rev 3, minor comment 6). It is suggested to revise to say: "...polyadenylation signal to generate a truncated transcript lacking most UPF3B exons."

Response: *We have modified the sentence as suggested.*

Line 307. Since this is a hypothesis, it should be definitive: "...UPF3A will not compensate..."

Response: *We have made this change.*

Line 358. This is a poor title. It is suggested to change to something like "EJC-dependent NMD is promoted by but does not require the UPF3 paralogs."

Response: *We have changed the title to "Some NMD can occur independently of both UPF3 paralogs".*

Line 361. It is suggested to also cite Karam et al. EMBO Reports 2015 here, as this paper also reported evidence of UPF3A/UPF3B-dependent and -independent substrates, albeit not on a genome-wide scale.

Response: *We have included this reference.*

Line 462. Add "the" between "without" and "EJC."

Response: *We have made the suggested change.*

Lines 465-467. This sentence undervalues the contributions of the authors data to the field. It is suggested to combine this sentence with the next in the following manner: "...cell lines addresses these questions, leading to an updated model..."

Response: We have made the suggested change.

Line 473. Shum et al. also provided evidence that UPF3A is a weak NMD activator.

Response: We have re-worded this sentence to make the suggested change.

Line 519. By "conservation," it appears that the authors mean fixation of the two UPF3 paralogs into vertebrate genomes. It is suggested to revise this sentence by saying something along the lines of: "It remains unclear what selective forces permitted the UPF3A paralog to be retained after duplication of the UPF3 gene at the dawn of the vertebrate lineage ~500 million years ago." It is suggested to follow this with a brief discussion of what these selective forces might be.

Response: We have made changes to the discussion along the suggested lines.

Previous Rev 3, minor comment 13. There was no response.

Response: We are sorry to have overlooked this comment ("(13) Fig. 6C. Would be more clears if legend indicated that * means values statistically significantly different from EGFP-transfected control cells, which has a value of 1.*"). We have modified the explanation in the current revision.*

Response to reviewers Fig 1

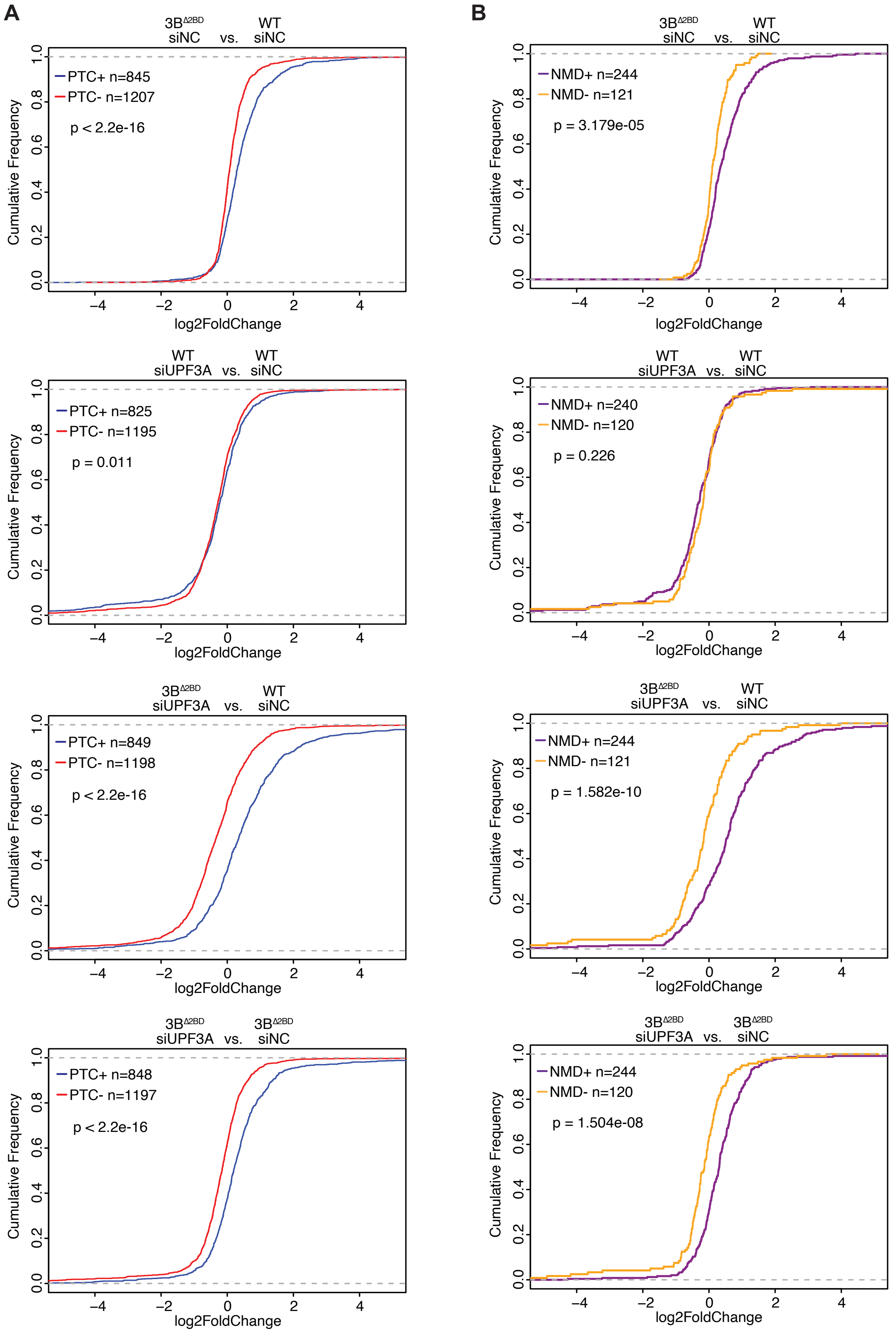

Response to reviewers Fig 1. Analysis of UPF3A/3B dependence of “stringent” PTC+ and NMD+ transcripts.

A. Cumulative Distribution Function (CDF) plots of PTC+ isoforms and PTC- isoforms from same set of genes. X-axis represents fold change in conditions listed above each plot. Number of transcripts in each set (n) and p-value from Kolmogorov-Smirnov (KS) test comparing the two distributions are shown. Only those PTC+ transcripts were included in this analysis that were also predicted to be NMD targets by Lykke-Andersen et al. (2014) *Genes & Development*, *28*(22), 2489–2517.

B. Cumulative Distribution Function (CDF) plots of NMD+ isoforms and NMD- isoforms from same set of genes. X-axis represents fold change in conditions listed above each plot. Number of transcripts in each set (n) and p-value from Kolmogorov-Smirnov (KS) test comparing the two distributions are shown. Only those NMD+ transcripts were included in this analysis that were also predicted to be NMD targets by Lykke-Andersen et al. (2014) *Genes & Development*, *28*(22), 2489–2517.

2nd Revision - Editorial Decision 31st Mar 2022

Thank you again for submitting the final revised version of your manuscript and addressing the remaining points. I am pleased to inform you that we have now accepted it for publication in The EMBO Journal.

EMBO PRESS

YOU MUST COMPLETE ALL CELLS WITH A PINK BACKGROUND

PLEASE NOTE THAT THIS CHECKLIST WILL BE PUBLISHED ALONGSIDE YOUR PAPER

Corresponding Author Name: Guramrit Singh
Journal Submitted to: EMBO J
Manuscript Number: EMBOJ-2021-109202R

Reporting Checklist For Life Sciences Articles (Rev. June 2017)

This checklist is used to ensure good reporting standards and to improve the reproducibility of published results. These guidelines are consistent with the Principles and Guidelines for Reporting Preclinical Research issued by the NIH in 2014. Please follow the journal's authorship guidelines in preparing your manuscript.

A- Figures
1. Data
The data shown in figures should satisfy the following conditions:

- the data were obtained and processed according to the field's best practice and are presented to reflect the results of the experiments in an accurate and unbiased manner.
- figure panels include only data points, measurements or observations that can be compared to each other in a scientifically meaningful way.
- graphs include clearly labeled error bars for independent experiments and sample sizes. Unless justified, error bars should not be shown for technical replicates.
- if n< 5, the individual data points from each experiment should be plotted and any statistical test employed should be justified
- Source Data should be included to report the data underlying graphs. Please follow the guidelines set out in the author ship guidelines on Data Presentation.

2. Captions

Each figure caption should contain the following information, for each panel where they are relevant:

- a specification of the experimental system investigated (eg cell line, species name).
- the assay(s) and method(s) used to carry out the reported observations and measurements
- an explicit mention of the biological and chemical entity(ies) that are being measured.
- an explicit mention of the biological and chemical entity(ies) that are altered/varied/perturbed in a controlled manner.

- the exact sample size (n) for each experimental group/condition, given as a number, not a range;
- a description of the sample collection allowing the reader to understand whether the samples represent technical or biological replicates (including how many animals, litters, cultures, etc.).
- a statement of how many times the experiment shown was independently replicated in the laboratory.
- definitions of statistical methods and measures:
  - common tests, such as t-test (please specify whether paired vs. unpaired), simple χ2 tests, Wilcoxon and Mann-Whitney tests, can be unambiguously identified by name only, but more complex techniques should be described in the methods section;
  - are tests one-sided or two-sided?
  - are there adjustments for multiple comparisons?
  - exact statistical test results, e.g., P values = x but not P values < x;
  - definition of 'center values' as median or average;
  - definition of error bars as s.d. or s.e.m.

Any descriptions too long for the figure legend should be included in the methods section and/or with the source data.

In the pink boxes below, please ensure that the answers to the following questions are reported in the manuscript itself. Every question should be answered. If the question is not relevant to your research, please write NA (non applicable). We encourage you to include a specific subsection in the methods section for statistics, reagents, animal models and human subjects.

USEFUL LINKS FOR COMPLETING THIS FORM

http://www.antibodypedia.com
http://1degreebio.org
http://www.equator-network.org/reporting-guidelines/improving-bioscience-research-repor
http://grants.nih.gov/grants/olaw/olaw.htm
http://www.mrc.ac.uk/Ourresearch/Ethicsresearchguidance/Useofanimals/index.htm
http://ClinicalTrials.gov
http://www.consort-statement.org
http://www.consort-statement.org/checklists/view/32-consort/66-title
http://www.equator-network.org/reporting-guidelines/reporting-recommendations-for-tumo
http://datadryad.org
http://figshare.com
http://www.ncbi.nlm.nih.gov/gap
http://www.ebi.ac.uk/ega
http://biomodels.net/
http://biomodels.net/miriam/
http://jjj.biochem.sun.ac.za
https://osp.od.nih.gov/biosafety-biosecurity-and-emerging-biotechnology/
http://www.selectagents.gov/

B- Statistics and general methods

Please fill out these boxes (Do not worry if you cannot see all your text once you press return)

1.a. How was the sample size chosen to ensure adequate power to detect a pre-specified effect size?	All the experiments are performed in a minimum of 3 replicates that is common for the field.
1.b. For animal studies, include a statement about sample size estimate even if no statistical methods were used.	No animals were used in this study.
2. Describe inclusion/exclusion criteria if samples or animals were excluded from the analysis. Were the criteria pre-established?	No animals were used in this study.
3. Were any steps taken to minimize the effects of subjective bias when allocating animals/samples to treatment (e.g. randomization procedure)? If yes, please describe.	Samples of different treatments from same replicates are processed at the same time.
For animal studies, include a statement about randomization even if no randomization was used.	No animals were used in this study.
4.a. Were any steps taken to minimize the effects of subjective bias during group allocation or/and when assessing results (e.g. blinding of the investigator)? If yes please describe.	Samples of different treatments from same replicates are processed at the same time.
4.b. For animal studies, include a statement about blinding even if no blinding was done	No animals were used in this study.
5. For every figure, are statistical tests justified as appropriate?	Yes.
Do the data meet the assumptions of the tests (e.g., normal distribution)? Describe any methods used to assess it.	We have applied non-parametric tests.
Is there an estimate of variation within each group of data?	Standard error is calculated for some of the experiments where applicable.

Is the variance similar between the groups that are being statistically compared?	Not checked.

C- Reagents

6. To show that antibodies were profiled for use in the system under study (assay and species), provide a citation, catalog number and/or clone number, supplementary information or reference to an antibody validation profile. e.g., Antibodypedia (see link list at top right), 1DegreeBio (see link list at top right).	Antibodies used in this study are listed in Appendix Table S5.
7. Identify the source of cell lines and report if they were recently authenticated (e.g., by STR profiling) and tested for mycoplasma contamination.	Cell lines are acquired from ATCC.

* for all hyperlinks, please see the table at the top right of the document

D- Animal Models

8. Report species, strain, gender, age of animals and genetic modification status where applicable. Please detail housing and husbandry conditions and the source of animals.	No live vertebrates were used in this study.
9. For experiments involving live vertebrates, include a statement of compliance with ethical regulations and identify the committee(s) approving the experiments.	No live vertebrates were used in this study.
10. We recommend consulting the ARRIVE guidelines (see link list at top right) (PLoS Biol. 8(6), e1000412, 2010) to ensure that other relevant aspects of animal studies are adequately reported. See author guidelines, under 'Reporting Guidelines'. See also: NIH (see link list at top right) and MRC (see link list at top right) recommendations. Please confirm compliance.	No live vertebrates were used in this study.

E- Human Subjects

11. Identify the committee(s) approving the study protocol.	No human subjects in this study.
12. Include a statement confirming that informed consent was obtained from all subjects and that the experiments conformed to the principles set out in the WMA Declaration of Helsinki and the Department of Health and Human Services Belmont Report.	No human subjects in this study.
13. For publication of patient photos, include a statement confirming that consent to publish was obtained.	No human subjects in this study.
14. Report any restrictions on the availability (and/or on the use) of human data or samples.	No human subjects in this study.
15. Report the clinical trial registration number (at ClinicalTrials.gov or equivalent), where applicable.	No human subjects in this study.
16. For phase II and III randomized controlled trials, please refer to the CONSORT flow diagram (see link list at top right) and submit the CONSORT checklist (see link list at top right) with your submission. See author guidelines, under 'Reporting Guidelines'. Please confirm you have submitted this list.	No human subjects in this study.
17. For tumor marker prognostic studies, we recommend that you follow the REMARK reporting guidelines (see link list at top right). See author guidelines, under 'Reporting Guidelines'. Please confirm you have followed these guidelines.	No human subjects in this study.

F- Data Accessibility

18: Provide a "Data Availability" section at the end of the Materials & Methods, listing the accession codes for data generated in this study and deposited in a public database (e.g. RNA-Seq data: Gene Expression Omnibus GSE39462, Proteomics data: PRIDE PXD000208 etc.) Please refer to our author guidelines for 'Data Deposition'.<br><br>Data deposition in a public repository is mandatory for:<br>a. Protein, DNA and RNA sequences<br>b. Macromolecular structures<br>c. Crystallographic data for small molecules<br>d. Functional genomics data<br>e. Proteomics and molecular interactions	Raw and processed data from RNA-Seq is available in GSE179843 at url:<br>https://www.ncbi.nlm.nih.gov/geo/query/acc.cgi?acc=GSE179843<br>Reviewers can use token "ypqjqkeorfexhkr" to access the data. Data will be publicly available upon the acceptance of the manuscript.
19. Deposition is strongly recommended for any datasets that are central and integral to the study; please consider the journal's data policy. If no structured public repository exists for a given data type, we encourage the provision of datasets in the manuscript as a Supplementary Document (see author guidelines under 'Expanded View' or in unstructured repositories such as Dryad (see link list at top right) or Figshare (see link list at top right).	All data is deposited to GEO.
20. Access to human clinical and genomic datasets should be provided with as few restrictions as possible while respecting ethical obligations to the patients and relevant medical and legal issues. If practically possible and compatible with the individual consent agreement used in the study, such data should be deposited in one of the major public access-controlled repositories such as dbGAP (see link list at top right) or EGA (see link list at top right).	No human subjects in this study.
21. Computational models that are central and integral to a study should be shared without restrictions and provided in a machine-readable form. The relevant accession numbers or links should be provided. When possible, standardized format (SBML, CellML) should be used instead of scripts (e.g. MATLAB). Authors are strongly encouraged to follow the MIRIAM guidelines (see link list at top right) and deposit their model in a public database such as Biomodels (see link list at top right) or JWS Online (see link list at top right). If computer source code is provided with the paper, it should be deposited in a public repository or included in supplementary information.	No computational models in this study.

G- Dual use research of concern

22. Could your study fall under dual use research restrictions? Please check biosecurity documents (see link list at top right) and list of select agents and toxins (APHIS/CDC) (see link list at top right). According to our biosecurity guidelines, provide a statement only if it could.	It does not.
